# Sound Packing DNA: packing open circular DNA with low-intensity ultrasound

**DOI:** 10.1038/srep09846

**Published:** 2015-04-20

**Authors:** DongHee Park, Bong-Kwang Jung, Hyunjin Park, Hyungbeen Lee, Gyudo Lee, Jingam Park, Unchul Shin, Jong Ho Won, Yong Jun Jo, Jin Woo Chang, Sangwoo Lee, Daesung Yoon, Jongbum Seo, Chul-Woo Kim

**Affiliations:** 1 Department of Pathology, Tumor Immunity Medical Research Center, Cancer Research Institute, Seoul National University College of Medicine, Seoul, Korea; 2 Department of Parasitology and Tropical Medicine, Seoul National University College of Medicine, Korea; 3 School of Electronic Electrical Engineering, Sungkyunkwan University, Suwon, Korea; 4 Department of Biomedical Engineering, Yonsei University, Wonju, Gangwon-do, Korea; 5 School of Public Health, Harvard University, Boston, Massachusetts, U.S.A; 6 Department of Neurosurgery, Yonsei University College of Medicine, Seoul, Korea; 7 School of Biomedical Engineering, Korea University, Seoul, Korea

## Abstract

Supercoiling DNA (folding DNA into a more compact molecule) from open circular forms requires significant bending energy. The double helix is coiled into a higher order helix form; thus it occupies a smaller footprint. Compact packing of DNA is essential to improve the efficiency of gene delivery, which has broad implications in biology and pharmaceutical research. Here we show that low-intensity pulsed ultrasound can pack open circular DNA into supercoil form. Plasmid DNA subjected to 5.4 mW/cm^2^ intensity ultrasound showed significant (p-values <0.001) supercoiling compared to DNA without exposure to ultrasound. Radiation force induced from ultrasound and dragging force from the fluid are believed to be the main factors that cause supercoiling. This study provides the first evidence to show that low-intensity ultrasound can directly alter DNA topology. We anticipate our results to be a starting point for improved non-viral gene delivery.

DNA supercoiling is a process wherein a double helix DNA is coiled into a higher-order helix form^1^,^2^,^3^. DNA supercoiling is a known tertiary structural characteristic of DNA. Physiologically, supercoiling is related to the regulation of DNA expression and is tightly controlled by the combined influences of many factors, including DNA binding proteins, transcription, replication, and topoisomerase, in a biological system^4^,^5^,^6^. Physically, supercoiling induces significant torsional and bending deformation on the DNA so that the supercoiled form of the DNA is energetically unfavorable^1^,^7^. The degree of DNA supercoiling is influenced by external conditions including ionicity and temperature^8^,^9^,^10^,^11^.

A biological system might experience damage if it is exposed to ultrasound at a relatively high intensity (tens of W/cm^2^) due to the local temperature increase and/or the transient cavitation of micro-sized bubbles induced by ultrasound^12^,^13^,^14^. Very small DNAs can be nicked and denatured by high-intensity ultrasound^15^. The effects of ultrasound exposure are different at a low intensity (hundreds of mW/cm^2^), which includes the acceleration of osteoblast response in bone healing^16^,^17^. Even small DNA related structures including chromatin architecture can be transiently influenced by ultrasound^18^. These findings indicate that ultrasound has the potential to alter the topology of DNA in the submicro- to micrometer range. In this study, we focused on the direct manipulation of DNA topology using very low-intensity ultrasound.

Ultrasound is capable of inducing radiation force, which in turn can cause movement of small particles in fluid. The governing equation of radiation force is as follows.


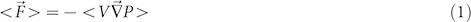
 where 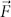 is force, 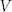 is volume, 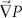
is absolute pressure gradient, and ‘<>’ denotes the time average^12^. Since force is proportional to an object’s volume, larger uncoiled linear and open circular DNA will be subject to stronger force. Considering the dragging force from the fluid, uncoiled DNA will experience bending force on its structure and this may be sufficient to cause supercoiling. We hypothesized that the proper low-intensity ultrasound sonication could physically and effectively supercoil a large population of uncoiled free DNA in fluid.

Two different experiments were designed using 4.7-kbp plasmid DNA (pDNA) to verify ultrasound induced DNA packing. First, heated pDNA was sonicated with low-intensity ultrasound. Electrophoresis was conducted with the sonicated pDNA and results were compared with those of unsonicated pDNA, with and without heating. Second, low-intensity ultrasound was applied to the purified pDNA, then the topology change of pDNA due to ultrasound was observed by atomic force microscopy (AFM), which has been successfully applied in structural studies of DNA^19^,^20^,^21^,^22^. The AFM images were further analyzed to quantify the degree of bending.

## Results

### Sonication on heated pDNA: Electrophoresis

An acoustic pressure of 126 kPa on the surface of the transducer with a 1% and 10% duty cycle and 100 Hz pulse repetition frequency was used for 30 seconds to generate the ultrasound intensity of 5.4 mW/cm^2^ and 53.6 mW/cm^2^, respectively. The electrophoresis was performed to confirm the topological change of pEGFP by ultrasound sonication. 20 μl of pEGFP was applied at each channel of agarose gel containing ethidium bromide in TAE buffer. Electrophoresis was operated as constant-voltage mode (5 V/cm) and carried out at the condition of 100 V, 95 mA, and 9.5 W for 30 min. After electrophoresis, the image of pEGFP was examined by UV-irradiation.

**Figure 1:**
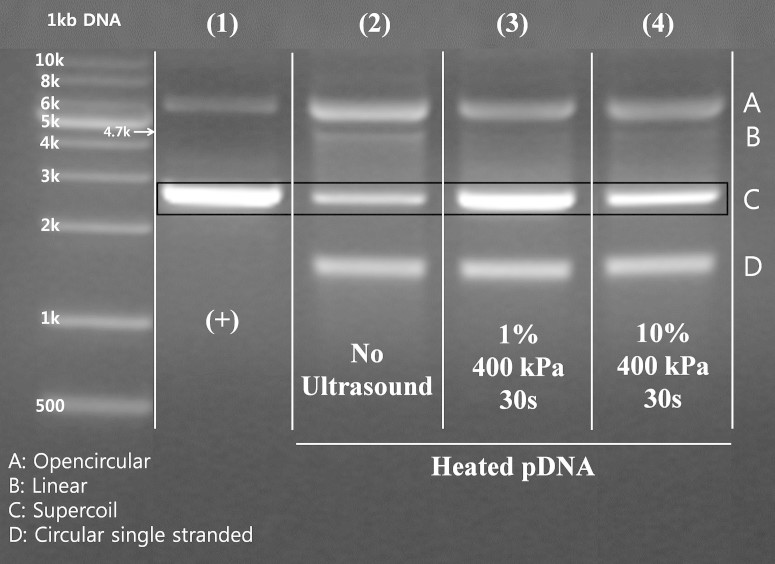
The results of electrophoresis in comparison to normal pEGFP, heated pEGFP, and heated pEGFP after ultrasound sonication. lane(1): normal pEGFP vector, lane(2): heated pEGFP vector, lane(3): heated pEGFP vector which is sonicated by ultrasound (pressure: 400 kPa, duty percentage: 1%, sonication time: 30 s), lane(4): heated pEGFP vector which is sonicated by ultrasound (pressure: 400 kPa, duty percentage: 10%, sonication time: 30 s).

The electrophoretic mobility of normal pEGFP (+), heated pEGFP (No Ultrasound), and heated and sonicated pEGFP were visualized with electrophoresis in Fig. 1. Each topological form of pEGFP is presented as open circular (row A), linear (row B), supercoiled (row C), and circular single-stranded (row D). Most pEGFPs obtained from the purification process are in the supercoiled form and heat changed the overall composition of DNA formation (see Fig. 1 column (1) and (2)). Applying heat led to significant increases in open circular, linear, and circular single-stranded forms of pEGFP (column (2)). After the sonication of low-intensity ultrasound on the heated pEGFPs, the open circular form of pEGFPs decreased significantly and the supercoiled form of pEGFPs increased significantly (column (3) & (4)). This indicated that many of the open circular pEGFPs were packed back to the supercoiled form, though the conformation of repacked pEGFP may not be identical to that of normal supercoiled pEGFP.

Interestingly, the degree of DNA packing does not appear to be proportional to the given ultrasonic energy, as observed in Fig. 1. Increasing the energy by increasing the sonication duty cycle did not result in an increased degree of DNA packing. Results indicate that the 1% duty cycle seems to be more efficient at inducing DNA packing than the 10% duty cycle condition (column (3) and (4)). Additionally, the supercoiled portion of column (3) appears located slightly lower than that of column (1) as can be seen in Fig. 1. Although minor differences of band positions in electrophoresis are generally induced due to differential heating, non-uniform electric field, and DNA concentration, the minor changes of topology could be the cause of this phenomenon. In other words, this difference in position can also be explained if most of the repacked supercoiled DNAs of column (3) are packed more tightly, compared to those in column (1). This led us to explore further details of the degree of packing of the 1% duty cycle condition by AFM imaging in the next section.

### Sonication on normal pDNA: AFM imaging

An acoustic pressure of 126 kPa on the surface of the ultrasound transducer with 1% duty cycle and 100 Hz pulse repetition frequency was used to generate the ultrasound intensity of 5.4 mW/cm^2^ for 30, 60, 120, and 300 seconds. We did not consider the 10% duty cycle, as we focused on the 1% duty cycle in the AFM experiment. All images were obtained at room temperature with a Multimode V tapping mode and commercial diving board silicon tips. The 512 × 512 pixels images were collected at a scan size of 5 μm at a scan-rate varying between 0.5 and 1 scan lines per second. For the statistical analysis, 10 AFM images each of control and sonication experiments were obtained from five sonication experiments

**Figure 2:**
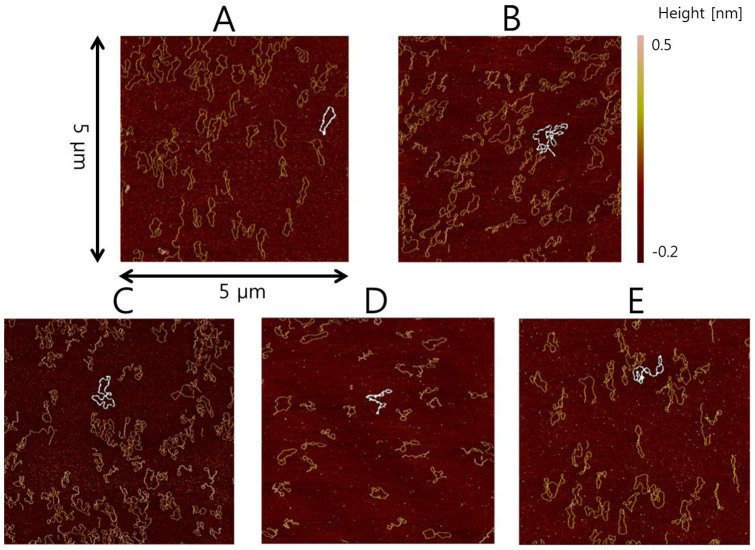
Representative samples of image-processed DNA images. A: control group with folding index value 116. B: 300-second group with folding index value 728. C: 30-second group with folding index value 244. D: 60-second group with folding index value 331. E: 120-second group with folding index value 590. White contours were the extracted contours of a given ROI using the image processing approach.

The AFM sample images of the normal pEGFP and the sonicated pEGFP are shown in Fig. 2. The sub-figures of Fig. 2 are assigned as follows. Fig. 2A is the control group, Fig. 2B is the 300-second group, Fig. 2C is the 30-second group, Fig. 2D is the 60-second group, and Fig. 2E is the 120-second group. As can be seen in Fig. 2A, some of the pEGFPs are supercoiled, while others are more relaxed to an extent that they appear open circular. On the other hand, most of pEGFP in Fig. 2B seem tightly condensed. Comparing Figs. 2A and 2B, the degree of supercoiling seems quite different between the two sub-figures. Figure 2A is the control case where no sonication was applied and Fig. 2B is the 30-second sonication case. Considering that most of purified pEFGPs are already in supercoiled form, these results indicate that low-intensity ultrasound packs the supercoiled pEGFPs into a tighter form. This result seems to agree with the downward shift of the supercoil band in Fig. 1. The visible differences among Fig. 2B, C, D, and E are less significant than those between Fig 2A and B. However, the longer exposure time at the 1% duty cycle appears to cause more supercoiling of pEGFPs or even entanglement among supercoiled pEGFPs.

Degree of folding, designated as folding index, was measured using curvature values of the contours of the DNA. Table 1 reports the folding index for all five groups. Consistent with the manual visual assessment, the folding index increased as we increased the sonication time. The differences between groups were all significant (corrected p < 0.05). Additionally, the mean value of the folding index increases in a proportional manner as the sonication time increases. Energy increased by increasing the duty cycle 10-fold reduced the number of supercoiled pEGFPs in the electrophoresis experiment. On the other hand, energy increased by increasing sonication time at a fixed duty cycle caused denser supercoiling of pEGFPs in the AFM imaging. These results indicate that duty cycle is more important than the energy of sonication in the given experiment for supercoiling pEGFPs.

## Discussion

Ultrasound can affect biological systems through periodic volume oscillation, heat deposition, cavitation of bubbles, and radiation force^23^,^24^. Among these mechanisms, heat effects were ignored in the current study, as results from temperature measurements with a thermocouple demonstrated that only a slight increase in temperature, 0.3°C (error range ± 0.1°C), was incurred during over 30 seconds of sonication at the 10% duty cycle; temperature change was not detectable for over 30 seconds sonication at the 1% duty cycle. Even though undetectable bubbles might exist in the fluid, violent transient cavitation could be excluded at the low amplitude (126 kPa) and mechanical index (0.12) used in our experiments. If bubble oscillation activity exists, then this will provide additional force to move DNA in fluid similar to the radiation force^12^. Periodic volume oscillation may not be guaranteed for freely floating DNA. On the other hand, radiation force and the induced movements of DNA seem to be the most likely mechanism to exert bending force on DNA.

Electrophoresis results alone do not provide quantitative numbers of change in formation, but the general trend of formation change could be confirmed. As indicated in Fig. 1, the supercoiling effect from the 10% duty cycle sonication is significantly lower than that from the 1% duty cycle sonication, even though the energy used was ten times higher. This is most likely due to the short 1 mm depth of fluid in the experiment. Larger relaxed open circular DNAs will experience stronger radiation force. Hence, the larger DNAs will be pushed down further during sonication. If the duration of sonication is too long compared to the DNA traveling distance, then we can expect most larger DNA to be pushed down to the bottom of the well. Once DNA is placed close to the bottom, additional friction force from the bottom surface of the well is expected. Bending of relaxed DNA requires movement in fluid; thus, the higher duty cycle of ultrasound sonication could be less effective for DNA packing in the small confined space used in our experiment. On the other hand, longer exposure to an efficient duty cycle (i.e., 1%) led to exposure time-proportionate bending of DNAs, as shown in Fig. 2 and Table 1. These two rather contradictory observations might confirm the role of radiation force in collective packing of DNAs.

**Figure 3:**
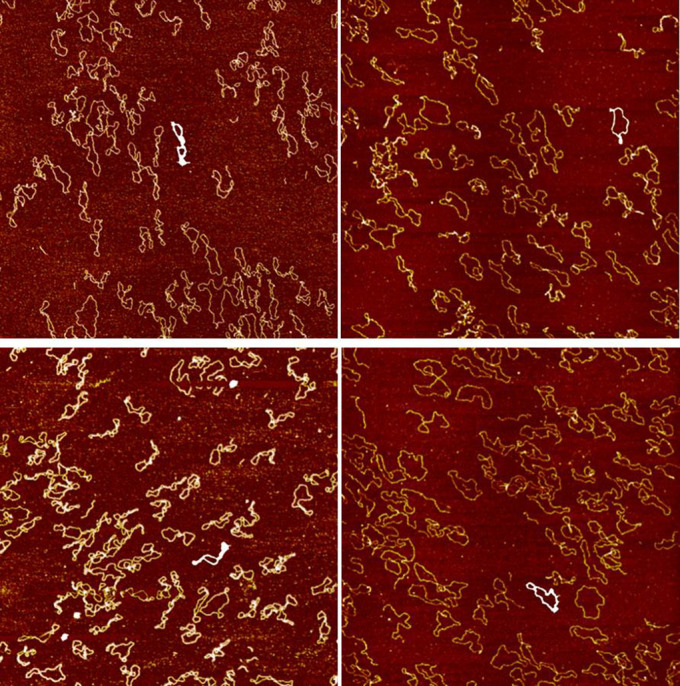
Representative sample of image processed DNA images for the follow up experiment. TOP LEFT: control group (at one hour) with folding index value 135. TOP RIGHT: control group (at one week) with folding index value 175. BOTTOM LEFT: 30-second group (at one hour) with folding index value 206. TOP RIGHT: 30-second group (at one week) with folding index value 121. White contours were the extracted contours of a given ROI using the image processing approach.

As indicated in the introduction, the supercoiled format of DNA is energetically unfavorable, so that the DNA packed by ultrasound can uncoil as time passes. Hence, we performed a follow-up experiment to explore whether sonication-induced folding could be reversed (i.e., unfolds or becomes less folded). The AFM images of the control and the 30-second group were taken at one hour and one week after the experiment, as can be seen in Fig. 3. The 30-second group was chosen since it was sufficiently effective for DNA packing. Only two images were taken for each group, at a given time point. We applied the same image processing procedures to quantify the degree of folding. We measured the folding index for 12 structures in a given group at each time point. The results were given in Table 2. P-values were not reported in the Table below since we did not accumulate enough samples for a fair comparison for the follow up experiment. For the control group, the folding index did not change significantly (130 (one hour) −> 150 (one week)) considering the SD value. For the 30-second group, the folding index reduced significantly (203 (one hour) −> 154(one week)) considering the SD value. We observed that the degree of folding decreased as time passed; thus, the process of sonication-induced folding was reversible to some extent, based on limited samples from the 30-second group. Further experiments are required to quantitatively measure the time constant for reversing the packing effect induced by ultrasound sonication.

DNA topology has received a great deal of attention in non-viral gene transfer, as DNA volume, which is determined by the DNA topology, is a critical factor in gene transfection efficiency^25^,^26^,^27^ The volume of a given DNA may be compacted up to four orders of magnitude by supercoiling^4^,^28^. Accordingly, efficiency of gene transfection can be significantly improved using smaller volume compact DNA (i.e., supercoiled DNA)^29^,^30^,^31^. DNA can be compacted or condensed in cationic-rich environments and further used for gene therapy^10^,^32^. While chemical condensation of DNA has been used most commonly, we explored DNA packing by physical forces induced with ultrasound in this study^33^,^34^,^35^.

## Methods

### Plasmid DNA

The 4.7-kbp pEGFP-C1 (Roche Applied Science, Switzerland) was amplified in *Escherichia coli* and purified using the Endo-free plasmid mega-kit (QIAGEN Mega kit CA, USA). The purity of pEGFP was evaluated by UV spectroscopy (260/E280 nm ratio) and the concentration of pEGFP was measured using NanoDrop ND-1000 UV-Vis Spectrophotometer (NanoDrop Technologies, Wilmington, DE, USA). The measured concentration was approximately 500 ng/μl. Two types of pEGFP were used in accordance with the method of analysis. For electrophoresis experiments, normal pEGFP was heated at 94°C for 180 sec and then gradually cooled at room temperature for about 30 minutes. For AFM imaging experiments, normal pEGFP without heating was used.

### Ultrasound sonication condition

**Figure 4:**
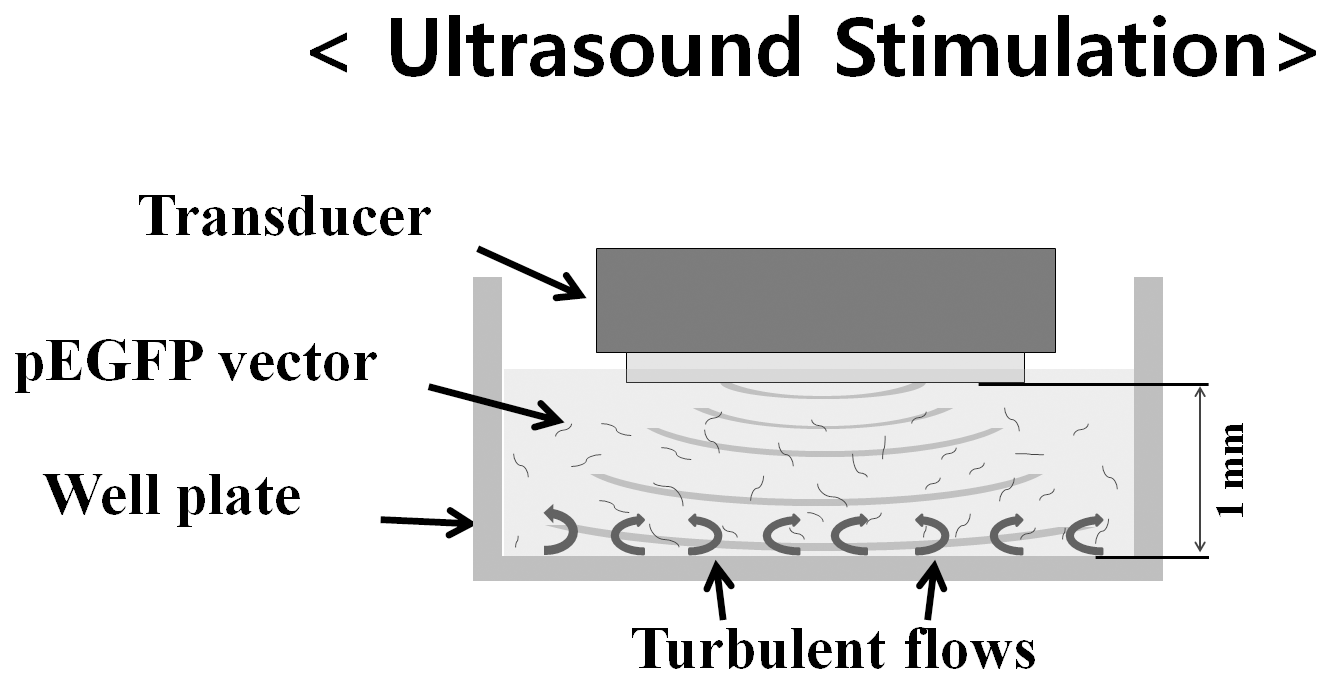
Experimental set up for ultrasound sonication to the pEGFP. Floating pEGFPs are free to move in the fluid by radiation force and turbulent-like microstreaming induced by ultrasound.

A single-element transducer with a 1/2-inch diameter aperture was used in the experiment at 1.12 MHz. The ultrasound transducer was positioned approximately 1 mm above the 12-well plate bottom, as can be seen in Fig. 4. In this near field area, the ultrasound field is spatially fast fluctuating, but the fluctuation amplitude is small^36^. Therefore, the exerted radiation force pattern will be in a spatially small scale in various directions accordingly. The pEGFPs pushed in a confined space by the radiation force are subject to complex turbulent-like circulation in fluid, which provides dragging force. These forces cause large relaxed pEGFPs to bend and supercoil.

For electrophoresis analysis, an acoustic pressure of 126 kPa on the surface of the transducer with a 1% and 10% duty cycle and 100 Hz pulse repetition frequency was used for 30 seconds to generate the ultrasound intensity of 5.4 mW/cm^2^ and 53.6 mW/cm^2^, respectively. Five hundred microliters of pEGFP solution, which was composed of the heated pEGFP and distilled water in the ratio of 1:50, was used in each well.

For AFM imaging experiments, an acoustic pressure of 126 kPa on the surface of the transducer with 1% duty cycle and 100 Hz pulse repetition frequency was used to generate the ultrasound intensity of 5.4 mW/cm^2^ for 30, 60, 120, and 300 seconds. One microliter of 500 ng/μl normal pEGFP was diluted in 1000 μl of AFM buffer solution (4 mM HEPES (4-(2-hydroxyethyl)-1-piperazinemethanesulphonic acid, pH 7.4), 10 mM NaCl, 2 mM MgCl2). Again, 500 μl of the prepared solution was used in each well.

### Agarose gel electrophoresis experiment

Electrophoresis was performed to confirm the topological change of pEGFP by ultrasound sonication. Twenty microliters of pEGFP was applied at each channel of 0.8% agarose gel containing agarose powder (8 mg/ml) with ethidium bromide (0.5 μg/ml) in TAE buffer (40 mM Tris base, 20 mM acetic acid and 1 mM EDTA, pH 8.0). Electrophoresis was performed using constant-voltage mode (5 V/cm) at 100 V, 95 mA, and 9.5 W for 30 minutes. After electrophoresis, the image of pEGFP was examined by UV-irradiation.

### Atomic force microscope experiment

The pEGFP solution (20 μl, 500 ng/ml) was dropped on freshly cleaved mica and incubated for one minute. The surface was then rinsed with 100 μl of pure water and gently blown dry with nitrogen to avoid aggregation of DNA. All atomic force microscopy (AFM) images were obtained in air at room temperature with a Multimode V (Veeco, CA, USA), operating in tapping mode. Commercial diving board silicon tips (TESP, Bruker, CA, USA) were used. The 512 × 512-pixel images were collected with a scan size of 5 μm at a scan-rate varying between 0.5 and 1 scan lines per second. For the statistical analysis, ten AFM images each of the control and sonication experiments were obtained from five sonication experiments.

### AFM image analysis procedures

The AFM images of pEGFPs were quantitatively analyzed by the degree of bending based on image processing approaches. Degree of folding can be measured by the amount of local curvature of the contours of the DNA supercoil^37^. Color images were converted to grayscale images summing red, green, and blue channels. A region of interest (ROI) enclosing one DNA structure was defined. All subsequent operations were performed on the ROI. ROI was low-pass-filtered using Gaussian kernel to remove noise. The contours of the DNA supercoil had brighter intensities than the background; thus, a threshold operation was performed to initially locate the contours. The initial extracted contours were further refined using image erosion and opening operation. The refined contours were parameterized using cubic interpolating splines. Local curvature was computed from second-order derivatives of the spline function. A sample mean value of the extracted contour was adopted. The process was repeated for supercoil structures, mostly in the center of the images, for all five groups of comparison. We assessed all contours for each AFM image. Contours that could not be properly processed were excluded using the following criterion; 1) partially viewable contours (those around the edge of the image), 2) contours failed to complete close loop, 3) contours heavily affected by background noise, and 4) contours which cannot be separated by ROI specification since they are either too close or occupy the same location. We considered 136 (control), 244 (30-second), 69 (60-second), 88 (120-second), and 217 (300-second) structures for image processing. The structures came from 7 (control), 6 (30-second), 4 (60-second), 3 (120-second), and 6 (300-second) images available for analysis. All the images were taken at the same scale; thus, the mean curvature values can be compared. The previous approach was implemented using MATLAB. We designated the curvature information as folding index, which is the primary parameter to quantify degree of folding in DNA shape. Figure 2 shows representative samples of image-processed DNA images of all sonication groups.

## Author contributions

J.S. and C.W.K. designed, planned and oversaw the project. D.P. and B.K.J. performed the experiments and analyzed the results. H. Park and J. Seo wrote the manuscript. G.L., H.L., J.W.C., S.L. and D.Y. interpreted the results and gave feedback on the project to the senior authors, J.S. and C.W.K., J.P., U.S., J.H.W. and Y.J.J. built experimental equipments and setups. All authors provided editorial comments and approved the final version of the manuscript.

## Supplementary Material

Supplementary Information Supplementary information

Supplementary Information Supplementary Information

## Figures and Tables

**Table 1:** Folding indices for various exposure times of sonication

	Control		30 sec sonication		60 sec sonication		120 sec sonication		300 sec sonication	
Folding index mean (SD)	181.3 (75.8)		262.63 (75.7)		311.7 (77.6)		494.1 (122.4)		640.7 (209.3)	
corrected p-value*		p-value <0.001		p-value <0.001		p-value <0.001		p-value <0.001		

*All p-values were significantly smaller than 0.001.

**Table 2:** Folding indices for the follow up experiments

	Control one hour	Control one week	30 sec sonication one hour	30 sec sonication one week
Folding index mean (SD)	130 (79)	150 (39)	203 (28)	154 (37)
