# Corrigendum: Sound Packing DNA: packing open circular DNA with low-intensity ultrasound

**DOI:** 10.1038/srep14588

**Published:** 2015-10-05

**Authors:** DongHee Park, Bong-Kwang Jung, Hyunjin Park, Hyungbeen Lee, Gyudo Lee, Jingam Park, Unchul Shin, Jong Ho Won, Yong Jun Jo, Jin Woo Chang, Sangwoo Lee, Daesung Yoon, Jongbum Seo, Chul-Woo Kim

Scientific Reports 5: Article number: 984610.1038/srep09846; published online: 04202015; updated: 10052015

This Article contains an error in the affiliation of Daesung Yoon. The correct affiliation is listed below:

Department of Bio-convergence Engineering, Korea University, Seoul, Korea

