# Supplementary material for: Sound Packing DNA: packing open circular DNA with low-intensity ultrasound
Source: Sci Rep. 2015 Apr 20;5:9846. doi: 10.1038/srep09846 (PMC4402968; doi:10.1038/srep09846)
Supplement: Supplementary Information [file srep09846-s1.doc]

Subject areas:

BIOMEDICAL ENGINEERING

dnA TOPOLOGY

Correspondence and requests for materials should be addressed to J.S. (jongbums@yonsei.ac.kr) and C.W.K. (cwkim@snu.ac.kr)

Sound Packing DNA: packing open circular DNA with low-intensity ultrasound

DongHee Park^1,6^, Bong-Kwang Jung^2,6^, Hyunjin Park^3,6^, Hyungbeen Lee^4^, Gyudo Lee^4^, Jingam Park^4^, Unchul Shin^4^, Jong Ho Won^4^, Yong Jun Jo^4^, Jin Woo Chang^5^, Sangwoo Lee^4^, Daesung Yoon^4^, Chul-Woo Kim^1*^, and Jongbum Seo^4*^

^1^Department of Pathology, Tumor Immunity Medical Research Center, Cancer Research Institute, Seoul National University College of Medicine, Seoul, Korea

^2^Department of Parasitology and Tropical Medicine, Seoul National University College of Medicine, Korea

^3^School of Electronic Electrical Engineering, Sungkyunkwan University, Suwon, Korea

^4^Department of Biomedical Engineering, Yonsei University, Wonju, Gangwon-do, Korea

^5^Department of Neurosurgery, Yonsei University College of Medicine, Seoul, Korea

^6^ These authors contributed equally to this work

*Corresponding author: Jongbum Seo, Department of Biomedical Engineering, Yonsei University, 304 Medical Industry Techno Tower, Wonju, Gangwon, *220-710,* Korea, Tel: +82-(0)33-760-2961; Fax: +82-(0)33-765-5483; E-mail: jongbums@yonsei.ac.kr

*Corresponding author: Chul Woo Kim, Tumor Immunity Medical Research Center & Cancer Research Institute, Department of Pathology, Seoul National University College of Medicine, 101 Daehak-no, Jongno-gu, Seoul 110-799, Korea, Tel:, +82-2-3668-7944; Fax: +82-(0)2-3676-7944; E-mail: cwkim@snu.ac.kr

**Supplement**

Image processing procedures are summarized in a flowchart below along with sample results of each sub-step (Supp Fig.1 and Supp Fig. 2). Sub-steps of the procedure are listed below.

1. Convert raw data in to grayscale image
2. Apply ROI mask to isolate one intended structure
3. Apply smoothing (Gaussian filter) to the masked data
4. Threshold the image to get a binary image
5. Erode and de-noise binary image to obtain the contour of the structure
6. Parameterize the contour using cubic interpolating splines
7. Assign second order derivative of the spline as the value of curvature


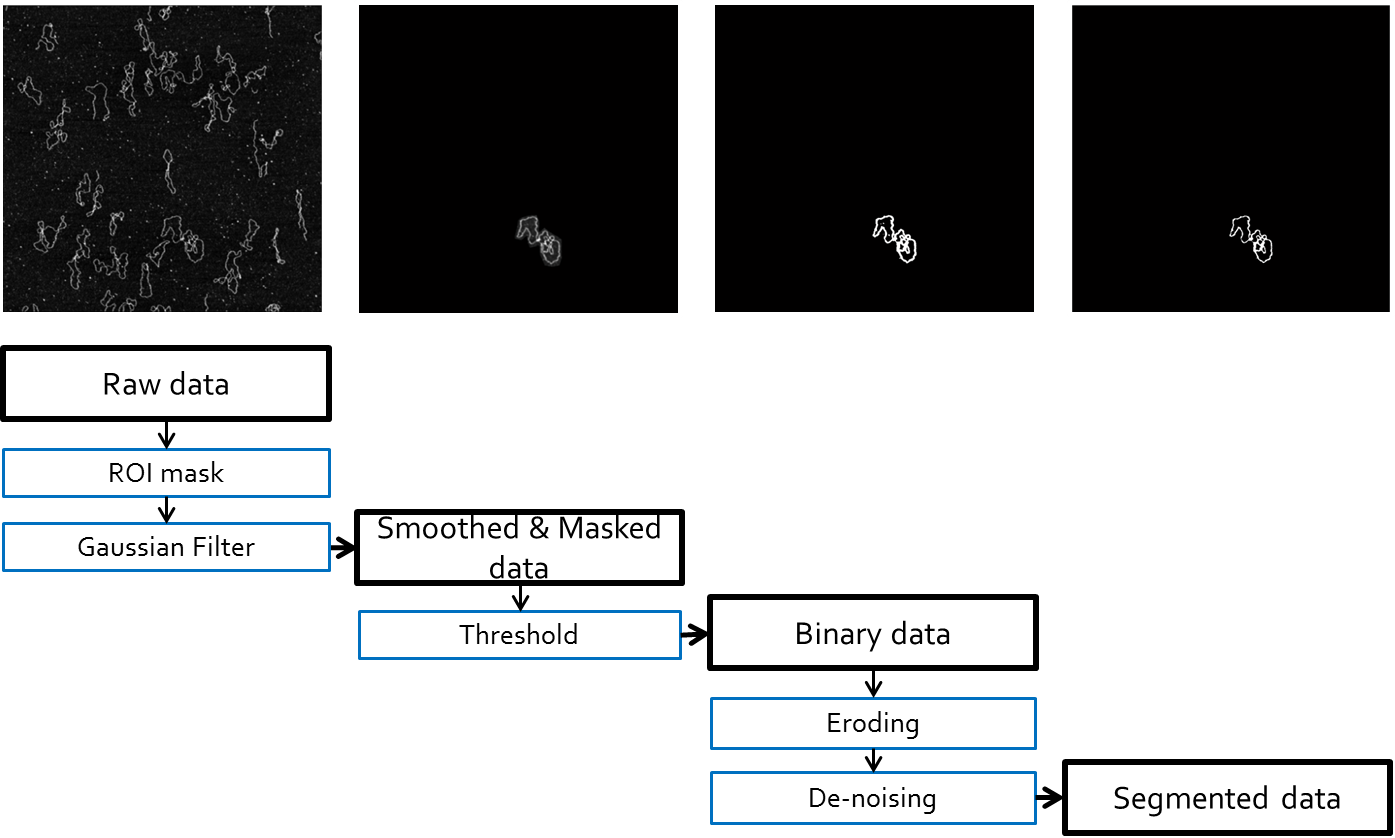


Supp Fig 1. Flowchart of the image processing procedures


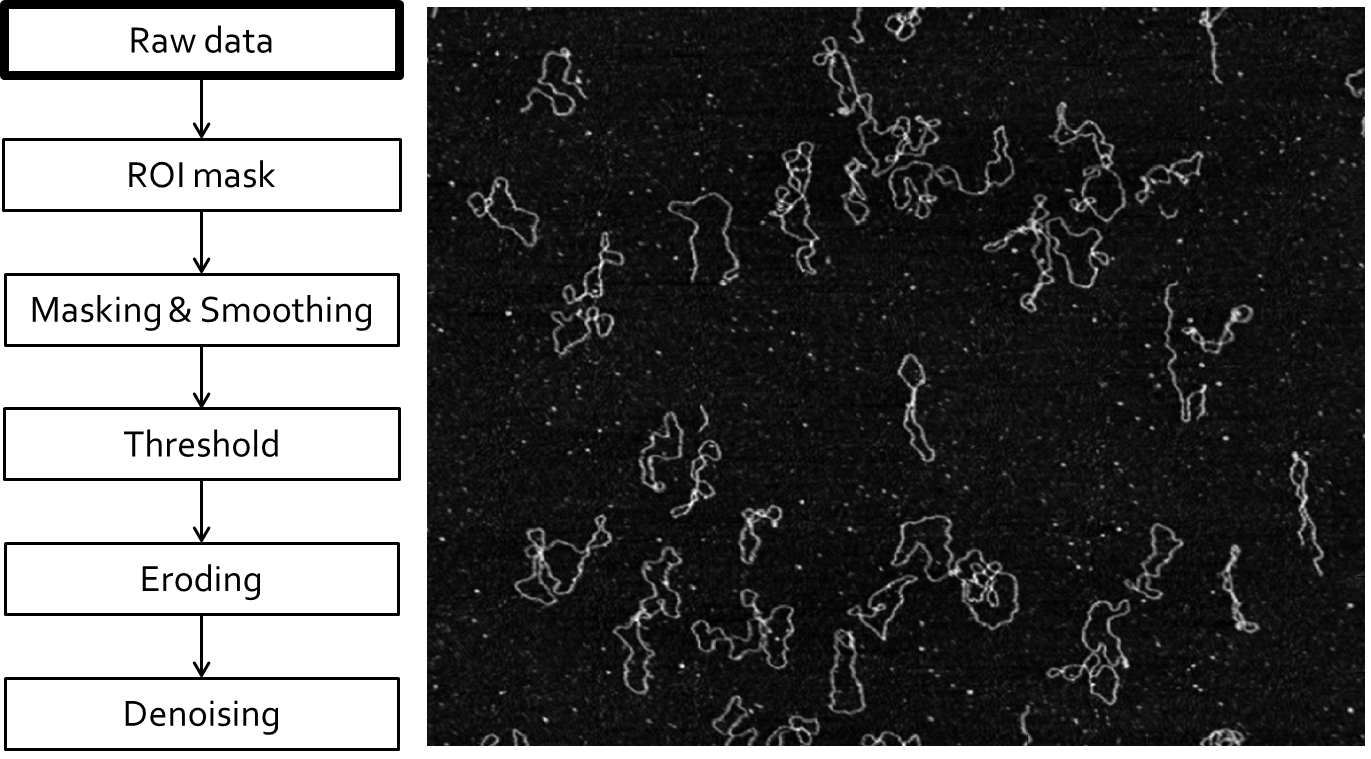


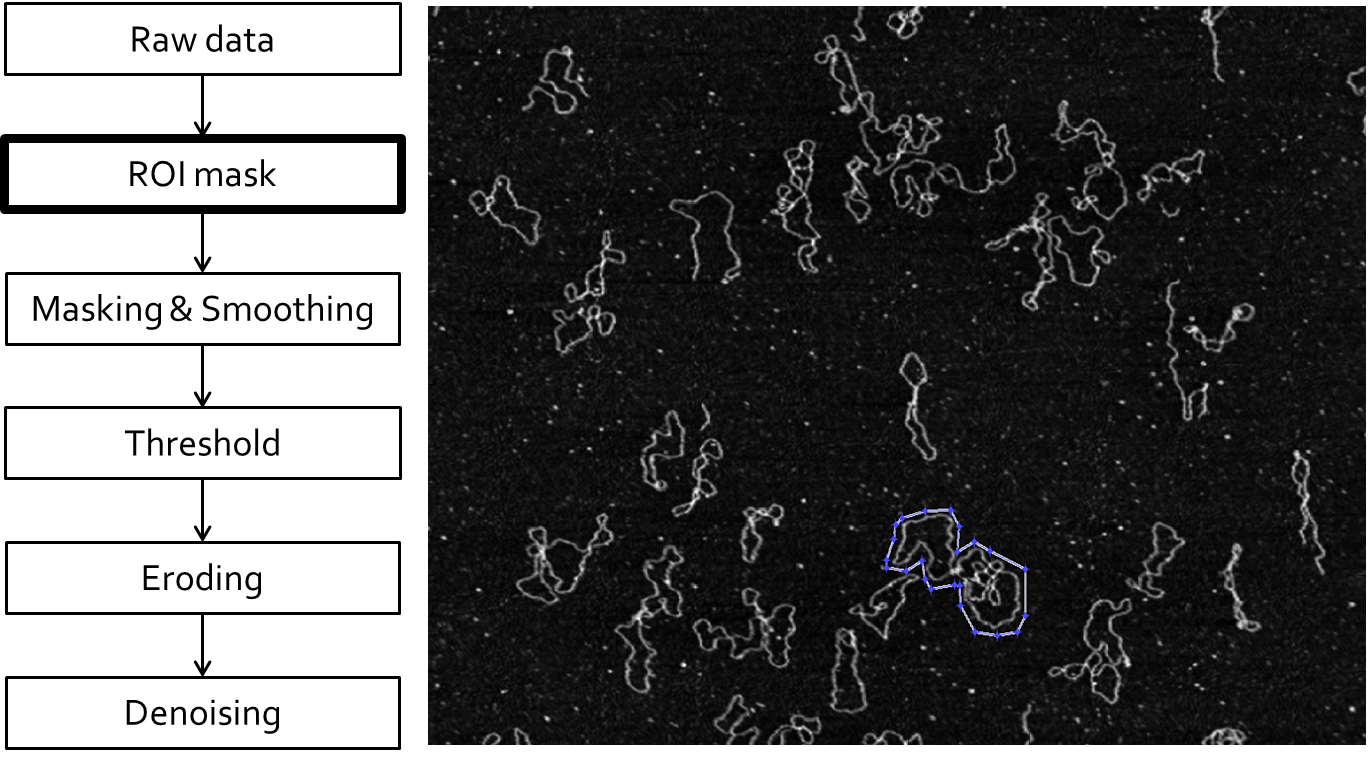


Supp Fig 2. Sample results of each sub-step of the procedures


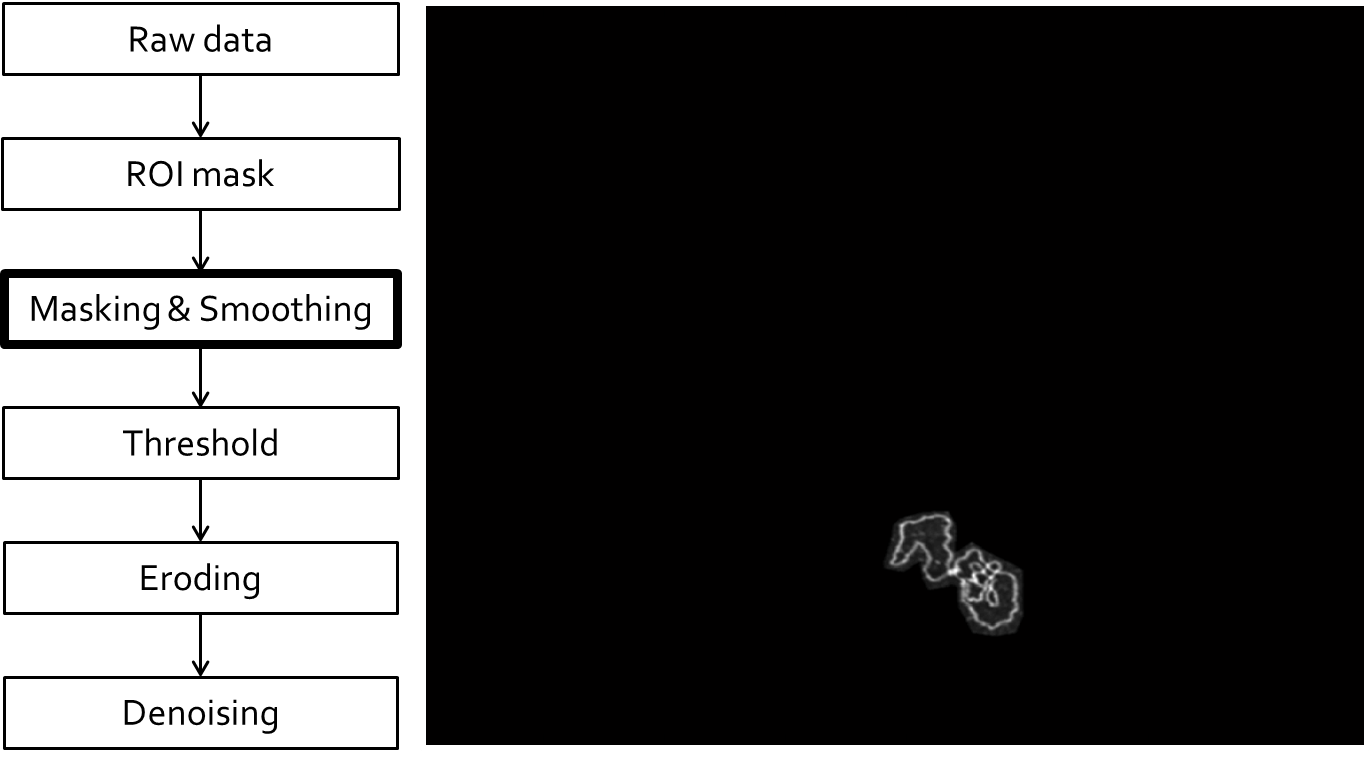

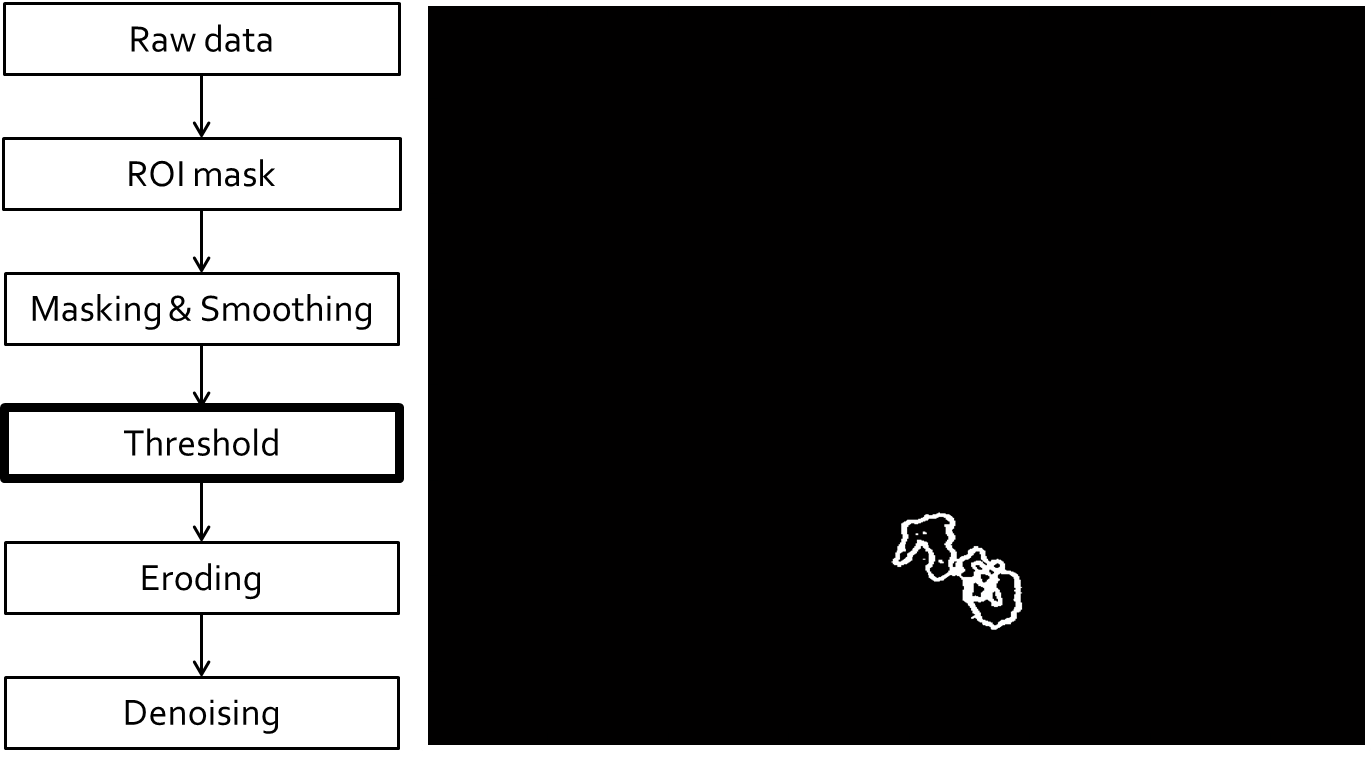


Supp Fig 2. Sample results of each sub-step of the procedures (continued)


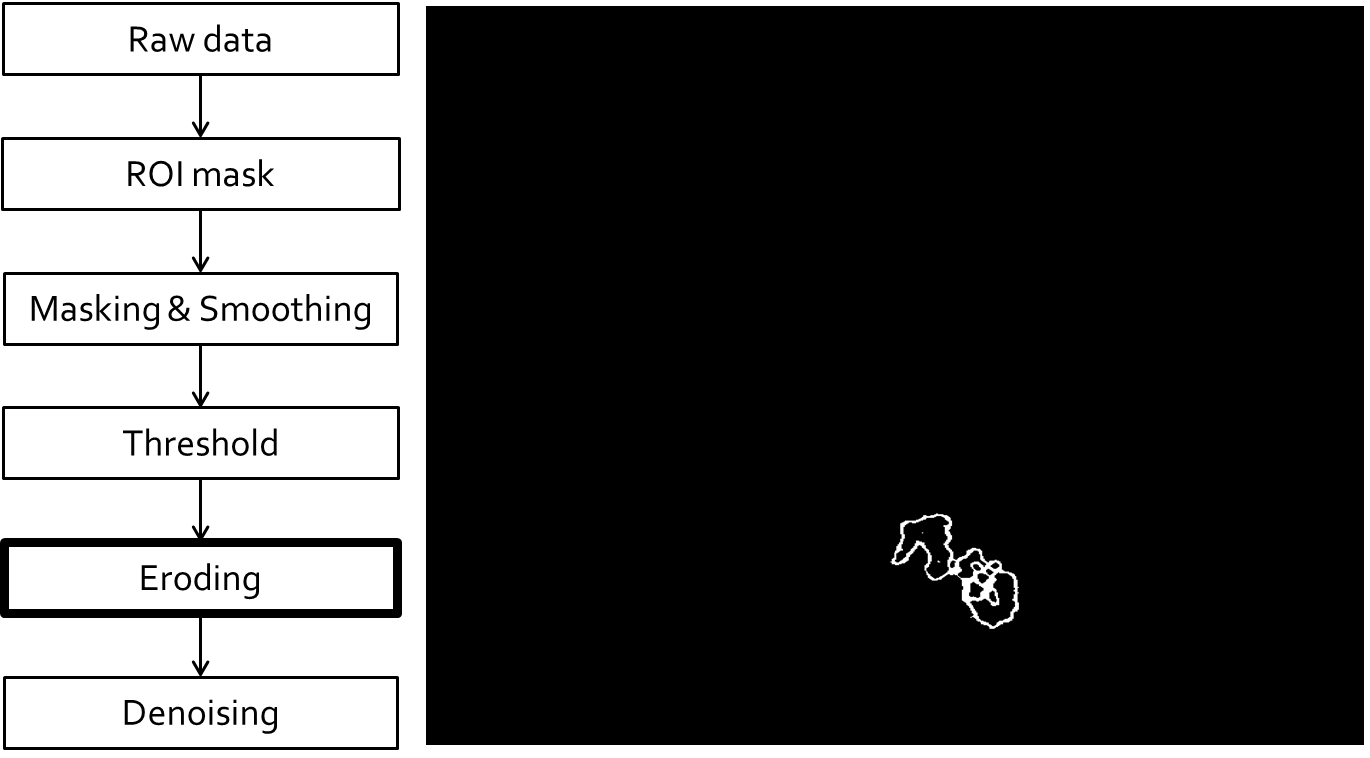


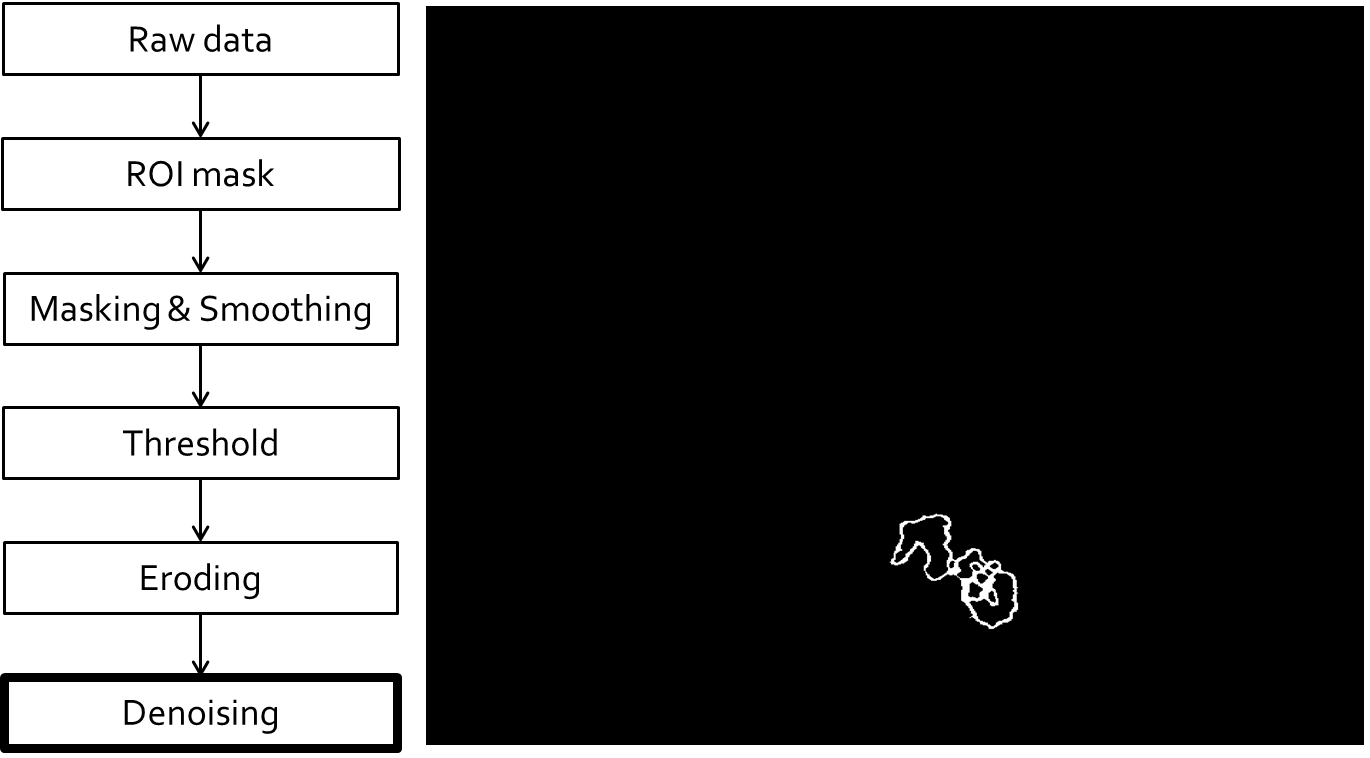


Supp Fig 2. Sample results of each sub-step of the procedures (continued)

**Supplementary Experiment**

As can be seen in Fig. 1, the result from electrophoresis experiment using heated pDNA shows a significant change in DNA conformation by low intensity ultrasound. Additionally, the supercoil band of heated-then-sonicated appears to downshift slightly compared to that of heated pDNA. In order to verify this downshift, two sets of supplementary experiments were conducted using both heated pDNA and normal pDNA. Although the formation change to supercoil band by ultrasound sonication may not be clear in normal DNA, downward shift phenomenon of the supercoil band can be observed.

Methods

The 4.7-kbp pEGFP-C1 (Roche Applied Science, Switzerland) was used in experiment of downward shift phenomenon. In case of heating, normal pEGFP was heated at 94˚C for 180 sec and then gradually cooled at room temperature for about 30 minutes. Acoustic pressure of 126 kPa on the surface of the transducer with a 1% duty cycle and 100 Hz pulse repetition frequency was used for 30 seconds to generate the ultrasound intensity of 5.4 mW/cm^2^. Five hundred microliters of pEGFP solution, which was distilled water in the ratio of 1:50, was used in each well. The ultrasound transducer was positioned approximately 1 mm above the 12-well plate bottom. After sonication, twenty microliters of sonication/non-sonicated pEGFP was applied at each channel of 0.8% agarose gel containing agarose powder (8 mg/ml) with ethidium bromide (0.5 μg/ml) in TAE buffer (40 mM Tris base, 20 mM acetic acid and 1 mM EDTA, pH 8.0). Electrophoresis was performed using constant-voltage mode (5V/cm) at 100 V, 95 mA, and 9.5 W for 30 minutes. We conducted identical experiments multiple times. Especially, the column position of each condition was alternately set to avoid the possibility of non-uniform electric field effect in electrophoresis.

Results


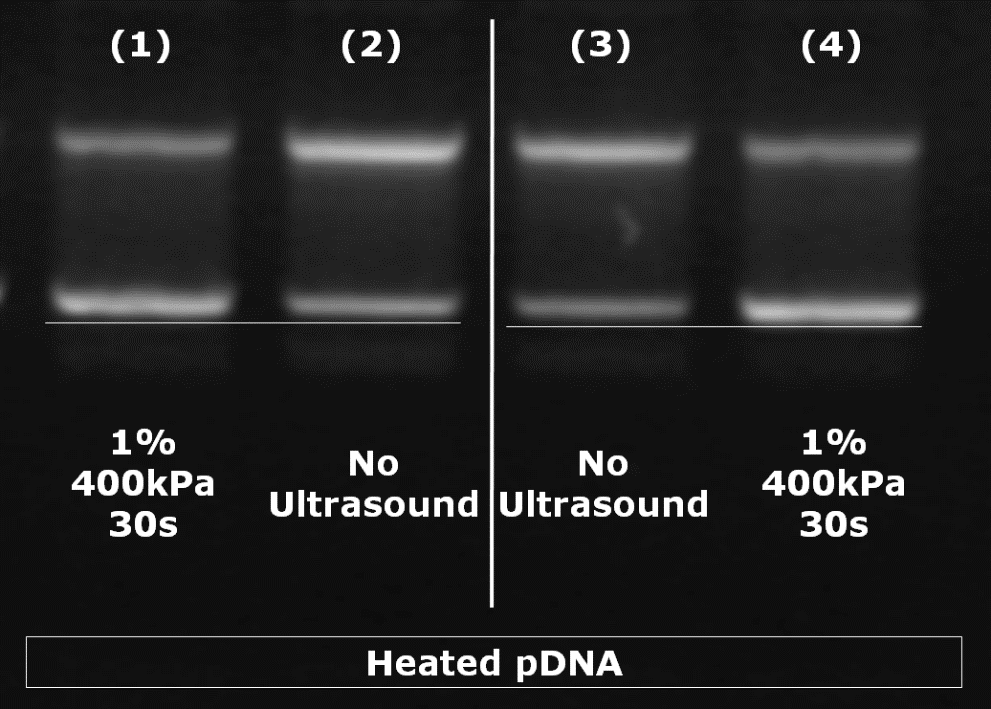


Supplementary Figure 3. The result of electrophoresis using sonicated/non-sonicated heated pEGFP on downward shift phenomenon of the supercoil band. lane(1): sonicated heated pEGFP, lane(2) and lane(3): heated pEGFP, lane(4): sonicated heated pEGFP.


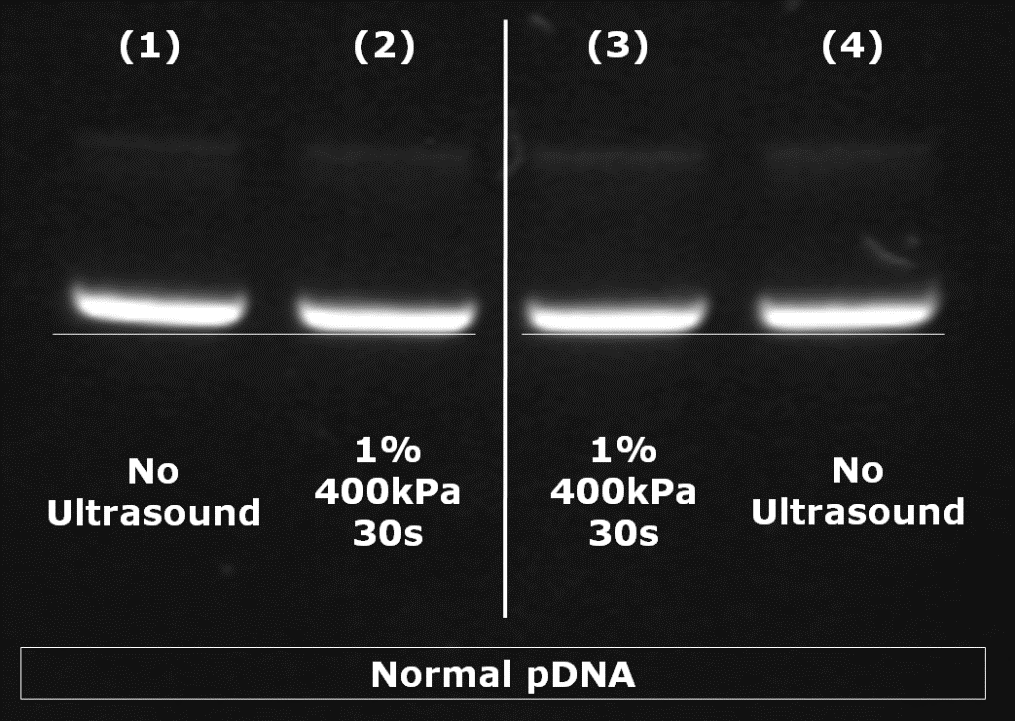


Supplementary Figure 4. The result of electrophoresis using sonication/non-sonicated pEGFP on downward shift phenomenon of the supercoil band. lane(1): normal pEGFP, lane(2) and lane(3): sonicated normal pEGFP, lane(4): normal pEGFP.

The representative results of electrophoresis on downward shift phenomenon of the supercoil band are shown in Supplementary Fig. 3 and Fig. 4. The downward shift phenomenon of the supercoil band appears to occur similarly in electrophoresis using normal pEGFP as well as heated pEFGP as can be seen in supplementary Fig. 4. Although there are slight differences among individual cases, most of results show a similar trend regardless of column positions. Based on the repeated experiment results, a slight downshift of supercoil band by low intensity ultrasound can be confirmed both in normal pEGFP and heated pEGFP.

Discussion

When the plasmid DNA (pDNA) is isolated, we can observe 2 or 3, or even 4 bands on the electrophoresis. This phenomenon is caused by the degree of purification on pDNA. pDNA can generally exist in three conformations: supercoiled, open-circular (oc), and linear. In general, the pDNA, which is purified by Endotoxin-Free Giga Prep Kit, has a supercoiled form mostly^1,2,3^. When the pDNA is purified, most impurities are filtered, but a small amount of impurities (especially, opencircular isoform) can be included in purified pDNA. Latulippe and Zydney^4^ have effectively separated each formations of plasmid DNA by electrophoresis using enzymatic digestion. Cruz et al^5^ also presented each isoforms of plasmid DNA. According to their results, this linear form is placed between supercoiled form and opencircular form.

We can confirm the isoform of the plasmid DNA used in our experiment through the figures below. The size of pDNA used in electrophoresis experiment is 4.7kbp. We confirmed the formation of pDNA by 1kb DNA ladder markers. The size of linear formation included in heated pDNA (lane 2) is verified with 1kb DNA ladder markers. In addition, as explained above, a large amount of supercoiled form and little amount of the open-circular form is included in normal pDNA. The open-circular form is also included in heated pDNA. The open-circular form in heated pDNA is positioned at parallel line with open-circular form in normal pDNA. The pDNA can be denatured by disruption of hydrogen bonds by lysis or extra-stimulation. In this case, the circular single stranded pDNA can be formed as smaller size than supercoiled form^2,6,7^. In addition, the circular single stranded form (line D), which is included in heated DNA, was not changed by ultrasound sonication. It means that the smaller sizes form such as fragments than supercoiled form may not return to supercoiled form again by ultrasound sonication. In other words, the object for DNA packing in current ultrasound sonication condition could be limited to linear and open-circular form.


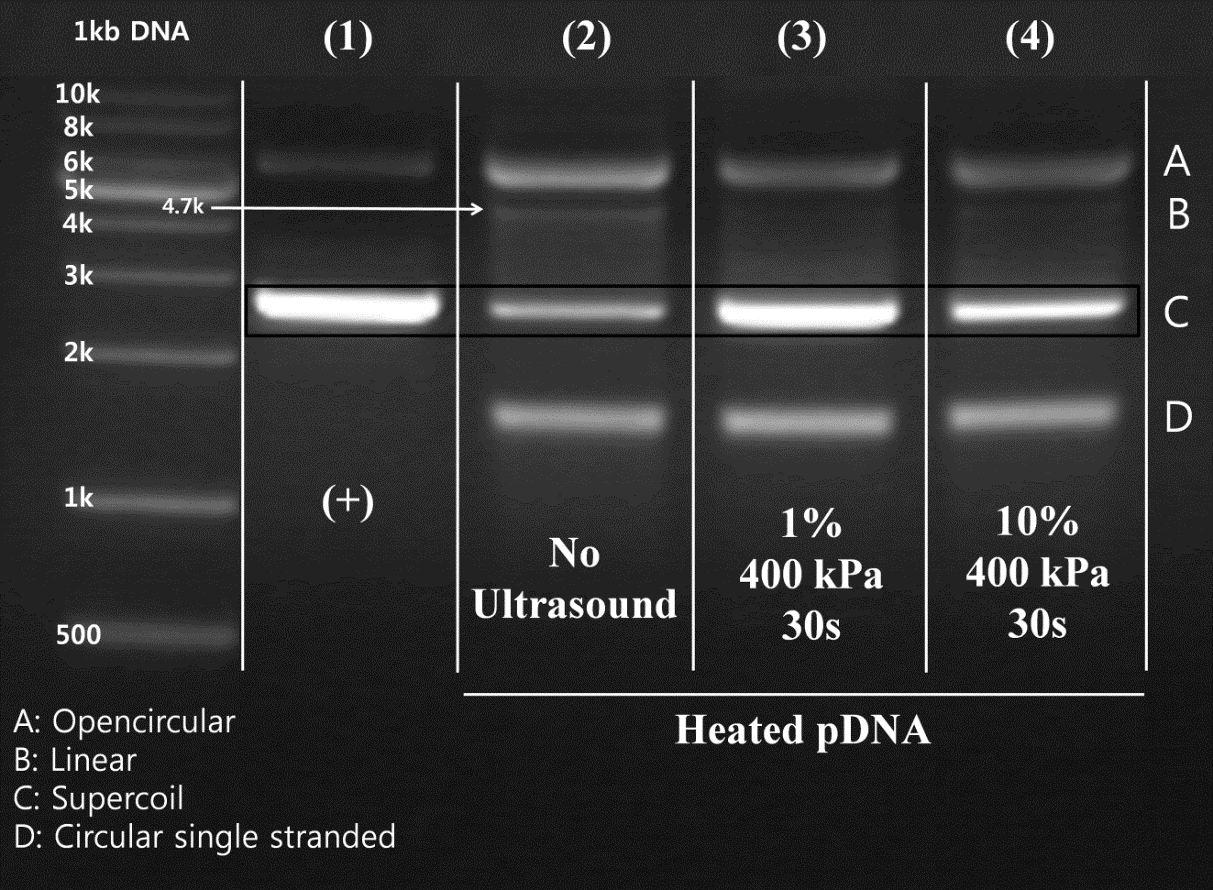


Supplementary Figure 5. The results of electrophoresis in comparison to normal pEGFP, heated pEGFP, and heated pEGFP after ultrasound sonication. lane(1): normal pEGFP vector, lane(2): heated pEGFP vector, lane(3): heated pEGFP vector which is sonicated by ultrasound (pressure:400kPa, duty percentage: 1%, sonication time: 30s), lane(4): heated pEGFP vector which is sonicated by ultrasound (pressure:400kPa, duty percentage: 10%, sonication time: 30s)

In addition, we confirmed size of pDNA used in experiment using restriction enzyme. As seen in Supplementary Figure 6, we can see the open-circular, linear, and supercoiled form in heated pDNA (first lane). The linear form is positioned on 4.7kb ladder marker line and open-circular form is on the line of linear form. In second lane, only linear form is located at 4.7k line. The plasmid is cut open with a restriction enzyme (cut at EcoRI site).


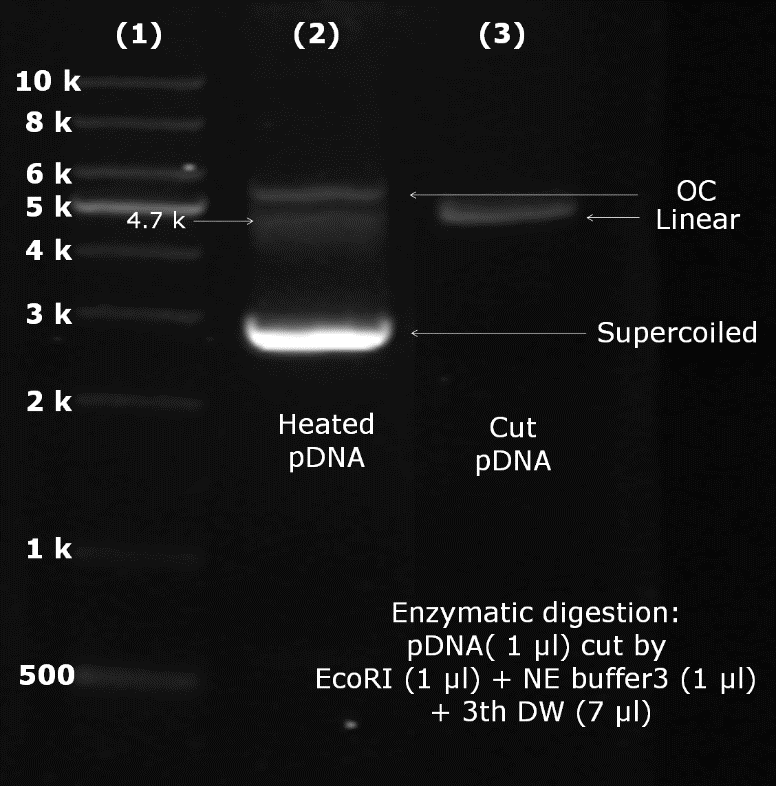


Supplementary Figure 6. Agarose gel electrophoresis image of 4.7 kbp heated DNA isoforms. lane(1): 1kb DNA ladder marker, lane(2): heated pEGFP vector, lane(3): linear isoform prepared by enzymatic digestion.

**References**

1 Katoch, O., Kaushik, S., Kumar, M. S., Agrawala, P. K. & Misra, K. Radioprotective property of an aqueous extract from valeriana wallichii. Journal of pharmacy & bioallied sciences 4, 327-332, doi:10.4103/0975-7406.103272 (2012).

2 Goldhaber-Gordon, I., Williams, T. L. & Baker, T. A. DNA recognition sites activate MuA transposase to perform transposition of non-Mu DNA. Journal of Biological Chemistry 277, 7694-7702 (2002).

3 Borujeni, E. E. & Zydney, A. L. Separation of plasmid DNA isoforms using centrifugal ultrafiltration. BioTechniques 53, 49-56 (2012).

4 Latulippe, D. R. & Zydney, A. L. Separation of plasmid DNA isoforms by highly converging flow through small membrane pores. Journal of colloid and interface science 357, 548-553 (2011).

5 Cruz, C., Sousa, A., Sousa, F. & Queiroz, J. A. Affinity analysis between immobilized l-arginine and plasmid isoforms provided by surface plasmon resonance. Analytical Methods 5, 1682-1686, doi:10.1039/C3AY40213D (2013).

6 Sehorn, M. G., Sigurdsson, S., Bussen, W., Unger, V. M. & Sung, P. Human meiotic recombinase Dmc1 promotes ATP-dependent homologous DNA strand exchange. Nature 429, 433-437 (2004).

7 Baumann, P., Benson, F. E. & West, S. C. Human Rad51 Protein Promotes ATP-Dependent Homologous Pairing and Strand Transfer Reactions In Vitro. Cell 87, 757-766, doi:http://dx.doi.org/10.1016/S0092-8674(00)81394-X (1996).
