# Supplementary figures and images for: Sound Packing DNA: packing open circular DNA with low-intensity ultrasound
Source: Sci Rep. 2015 Apr 20;5:9846. doi: 10.1038/srep09846 (PMC4402968; doi:10.1038/srep09846)

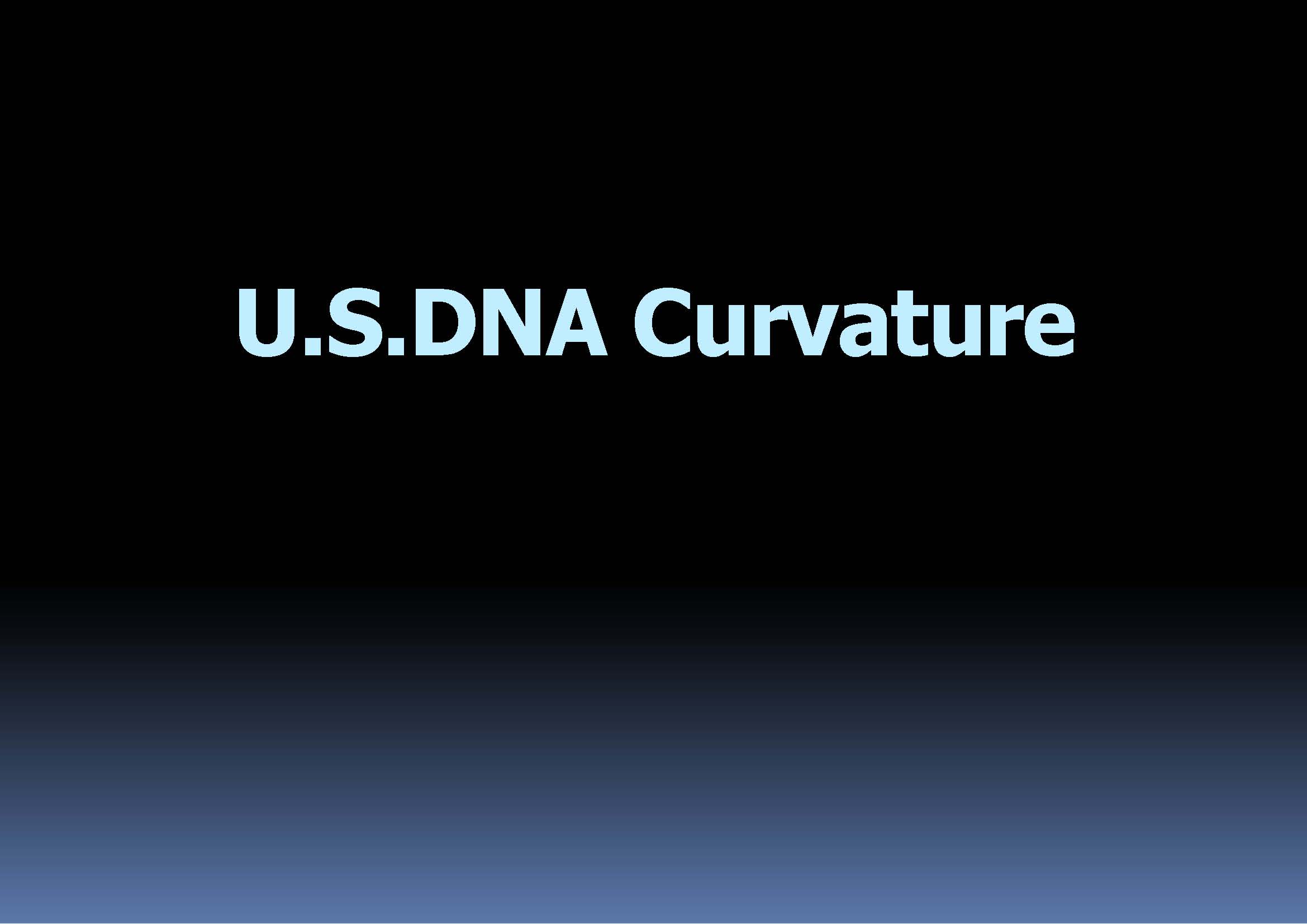

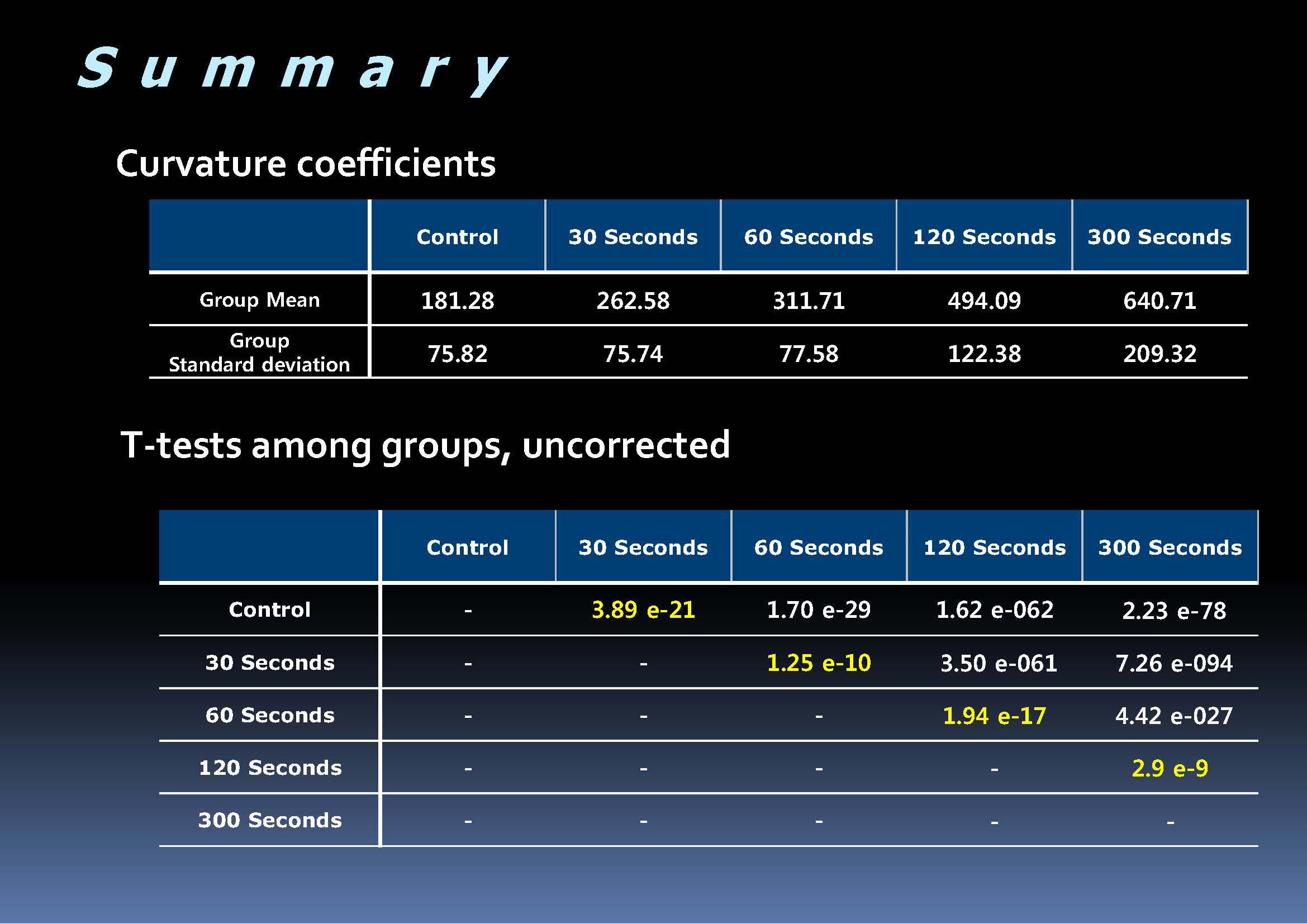

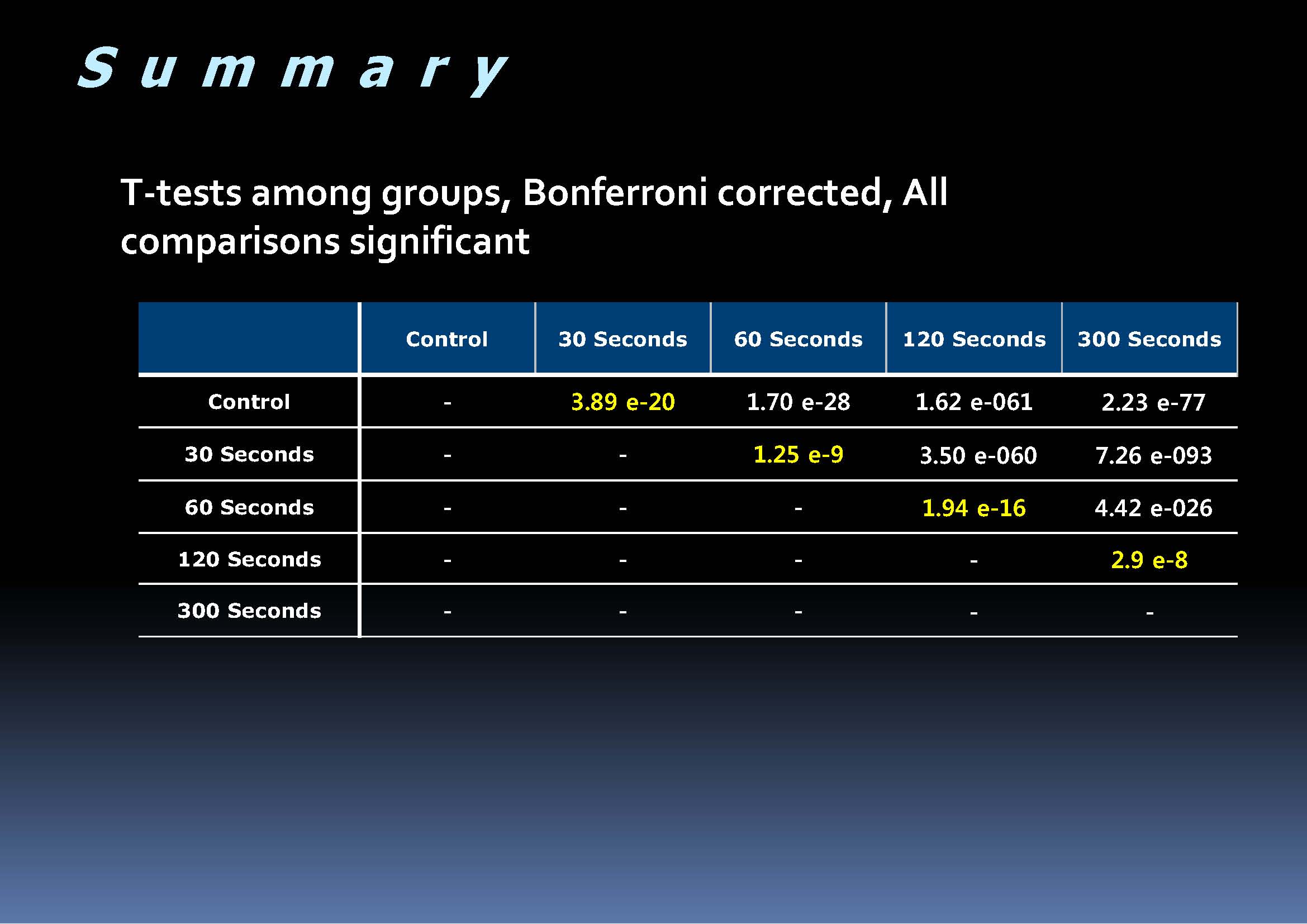

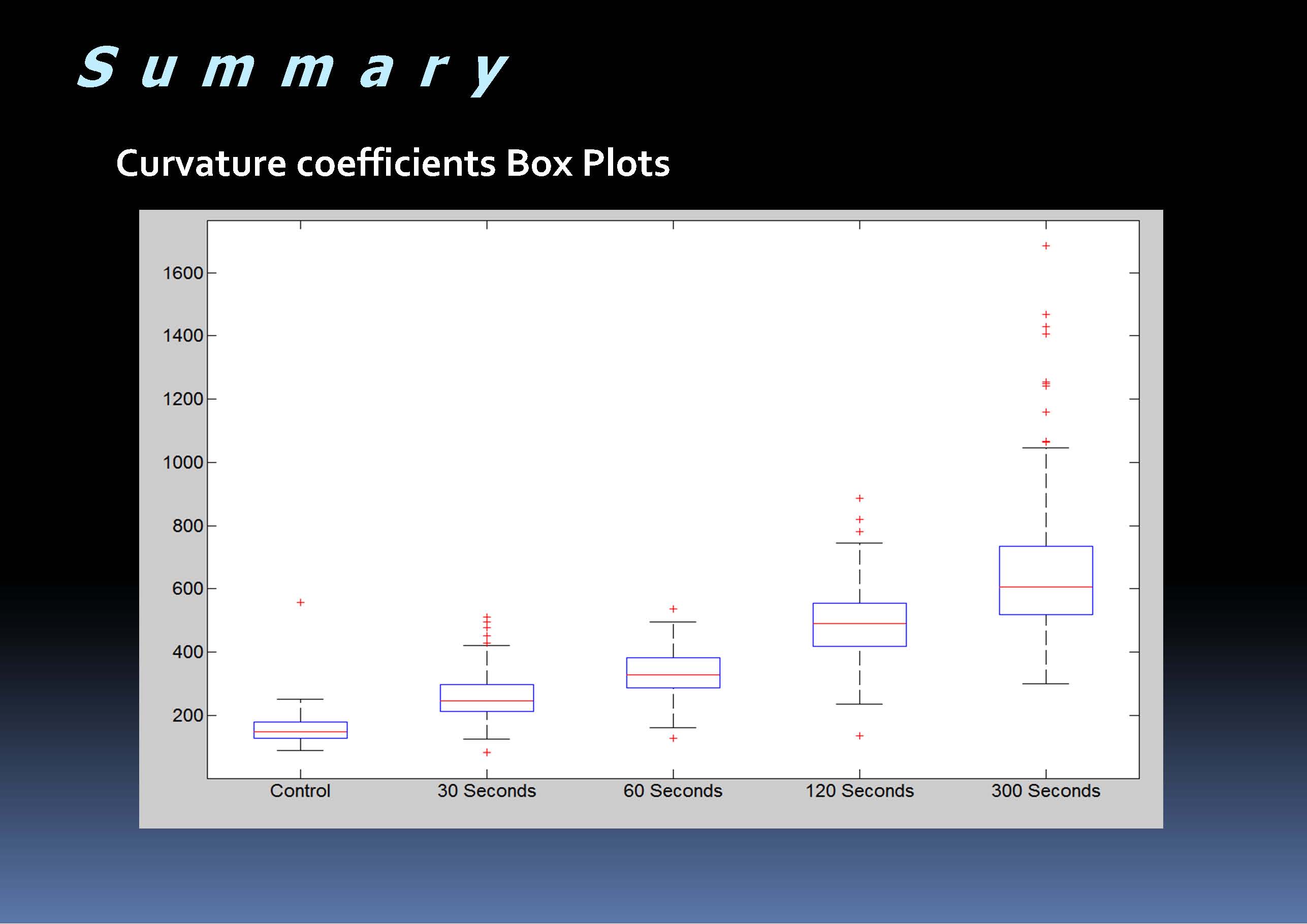

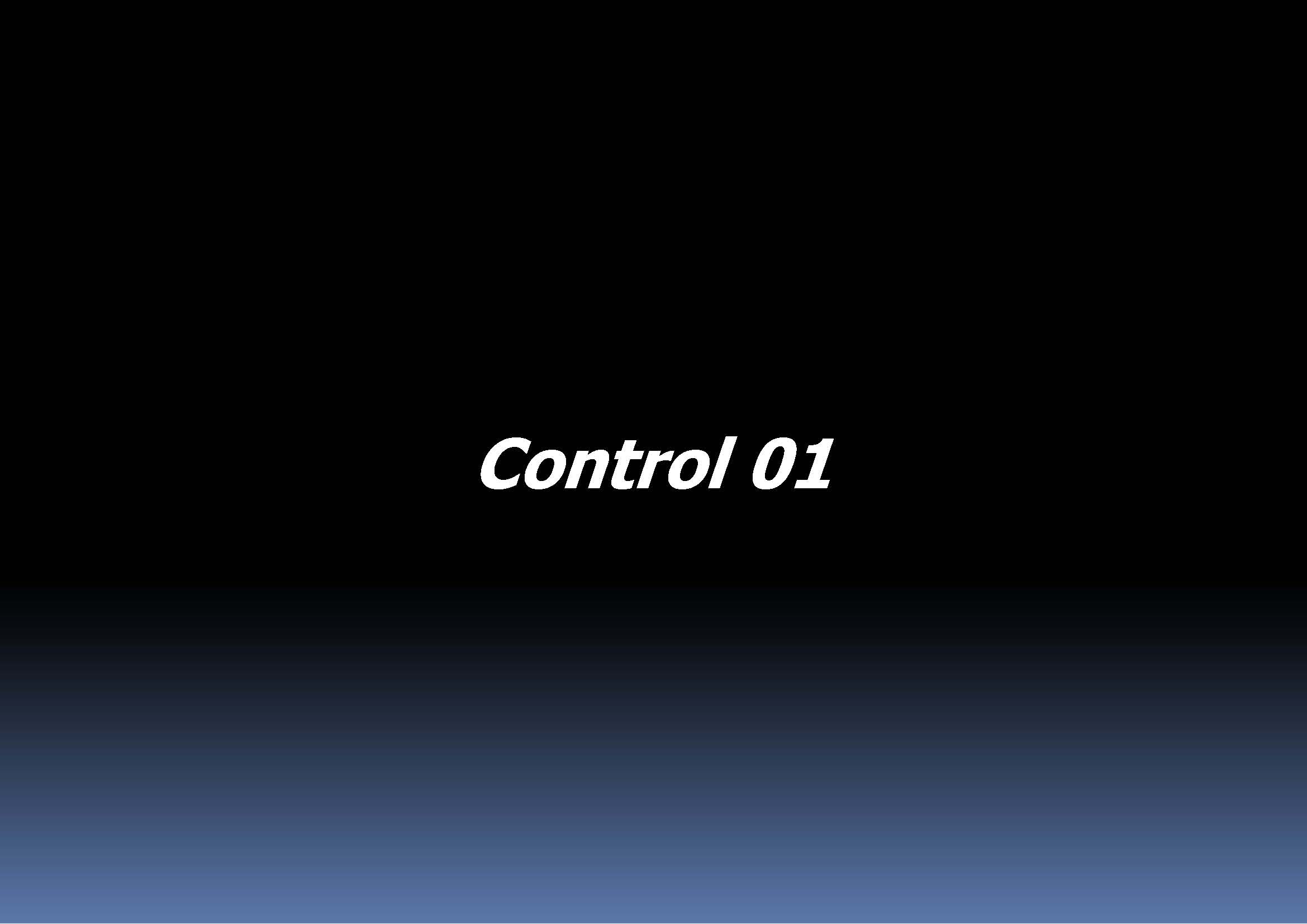

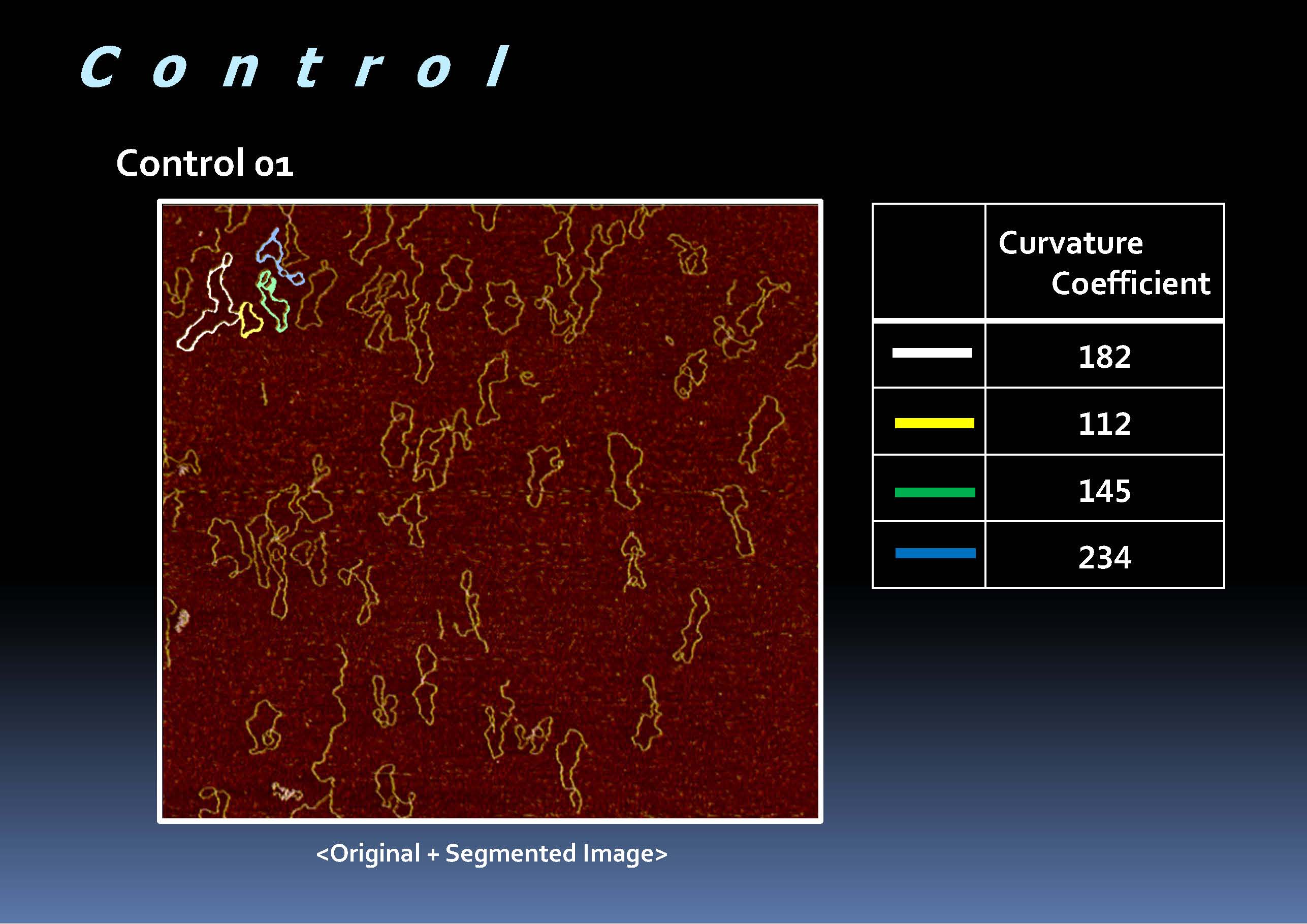

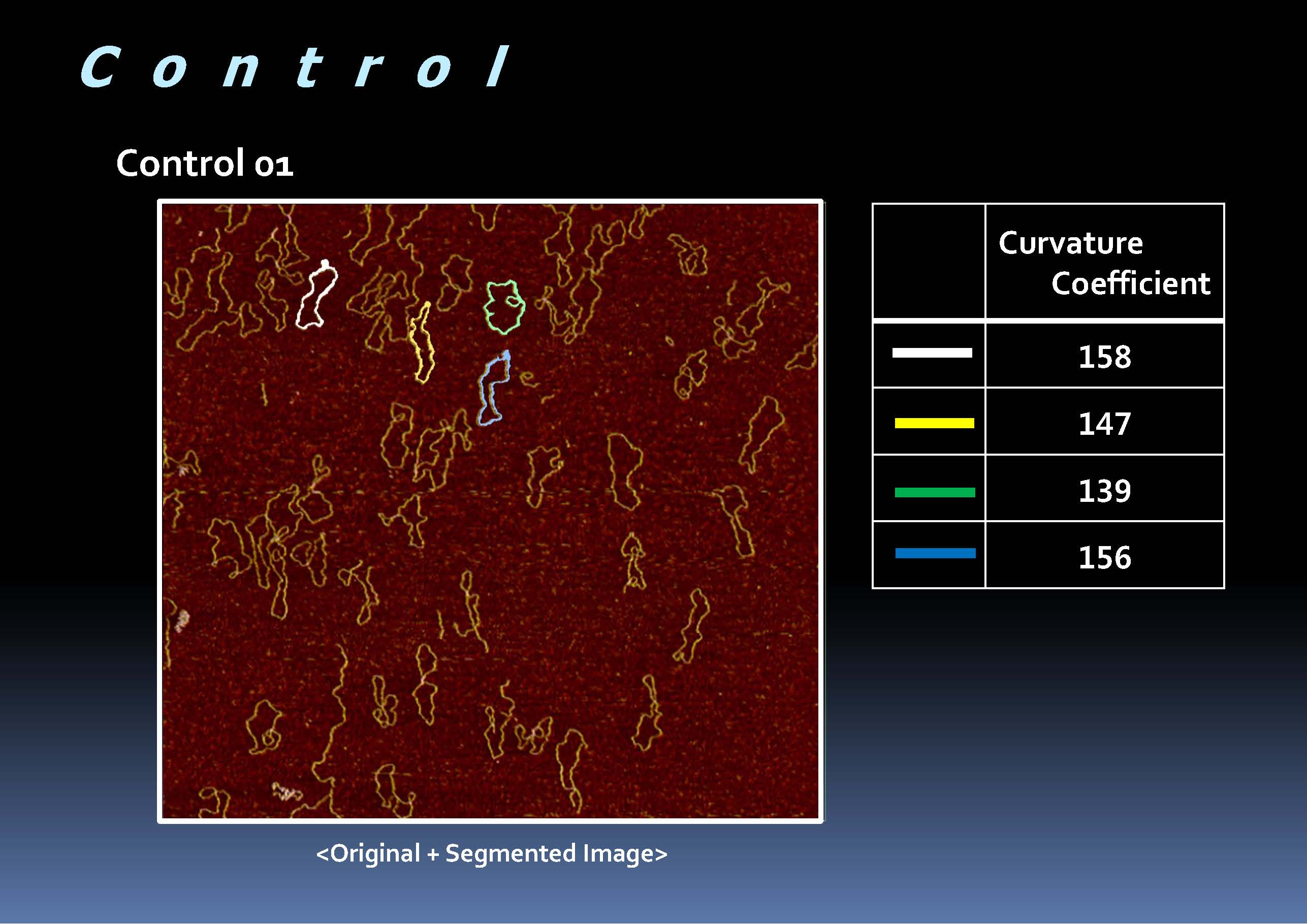

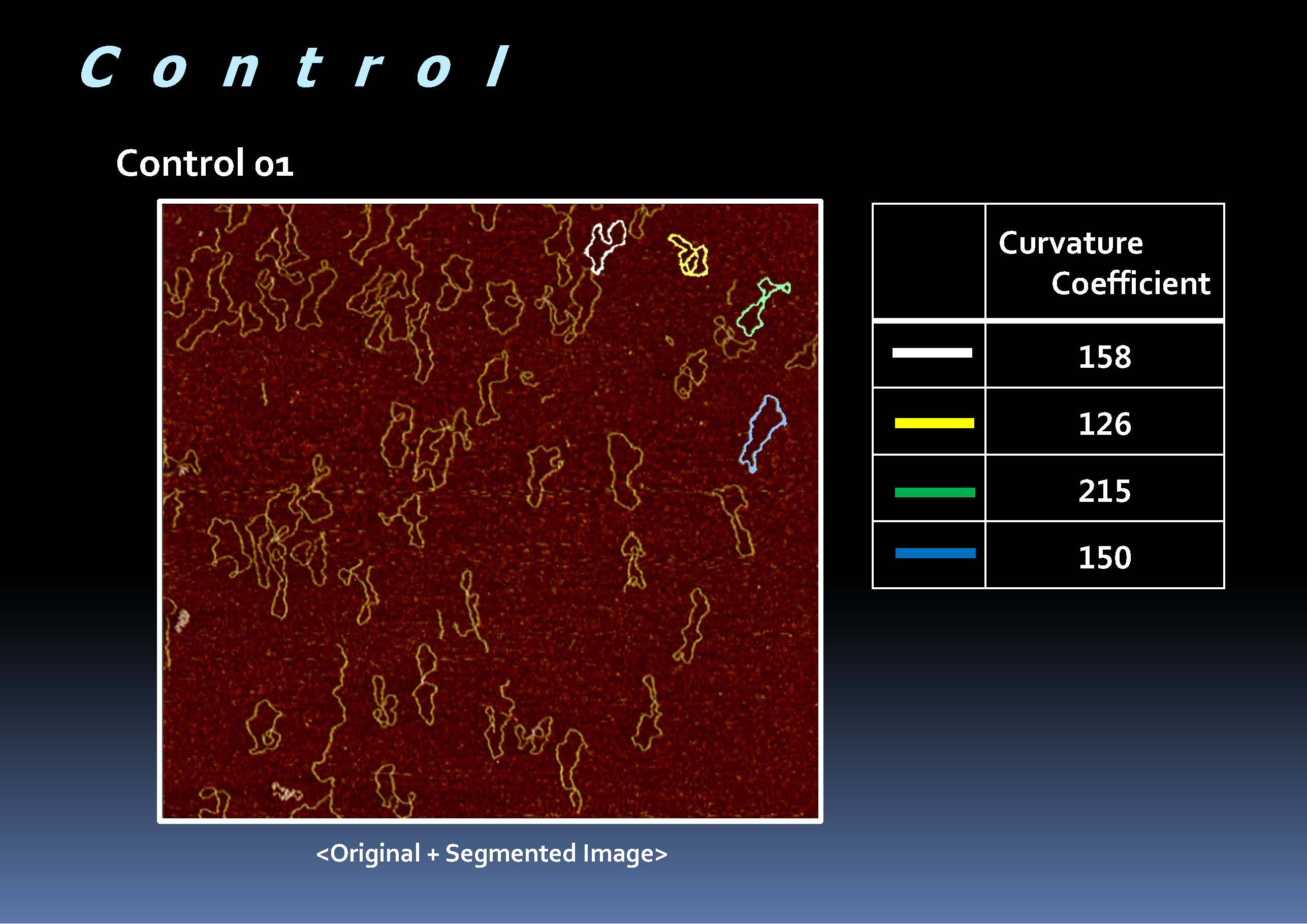

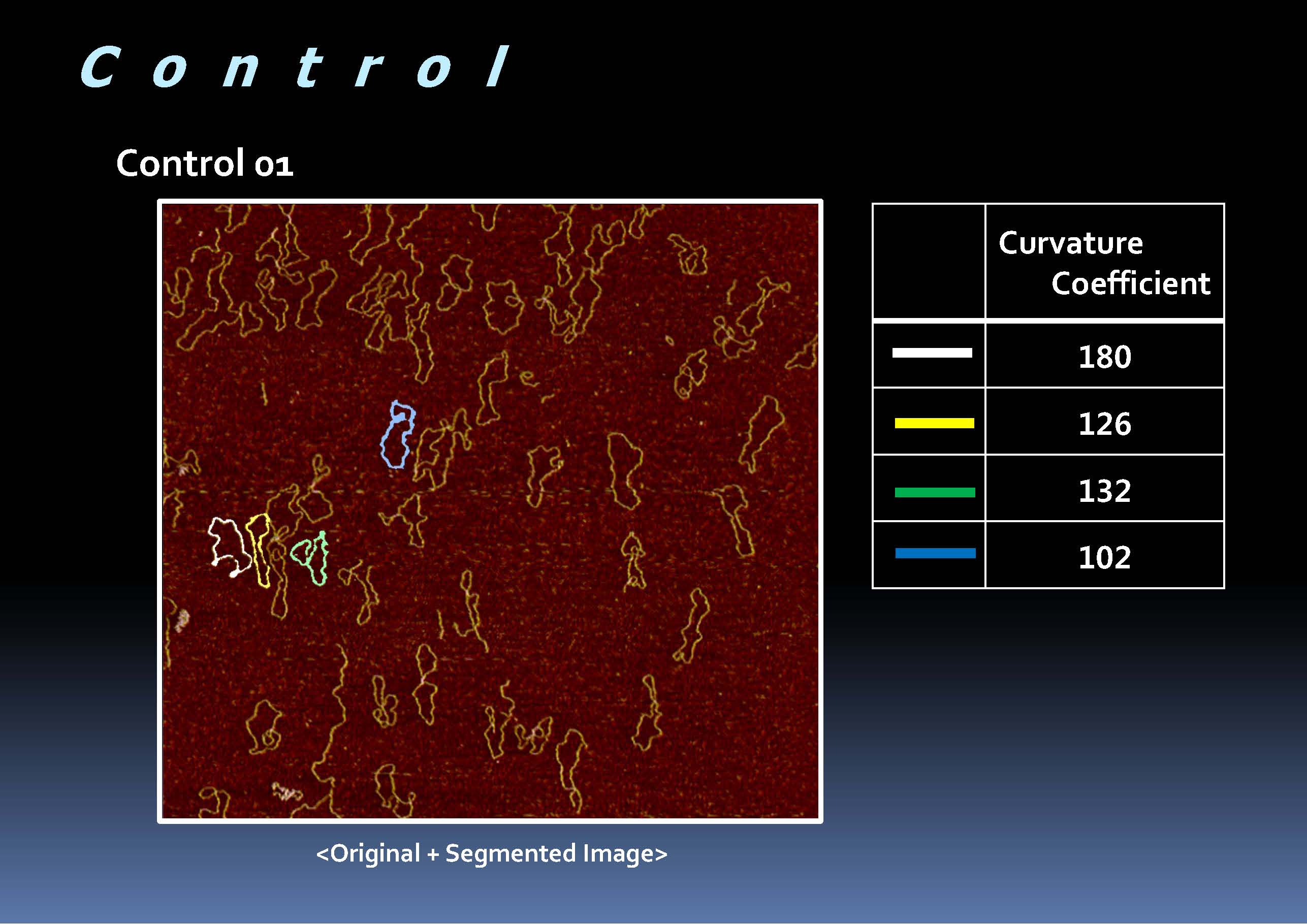

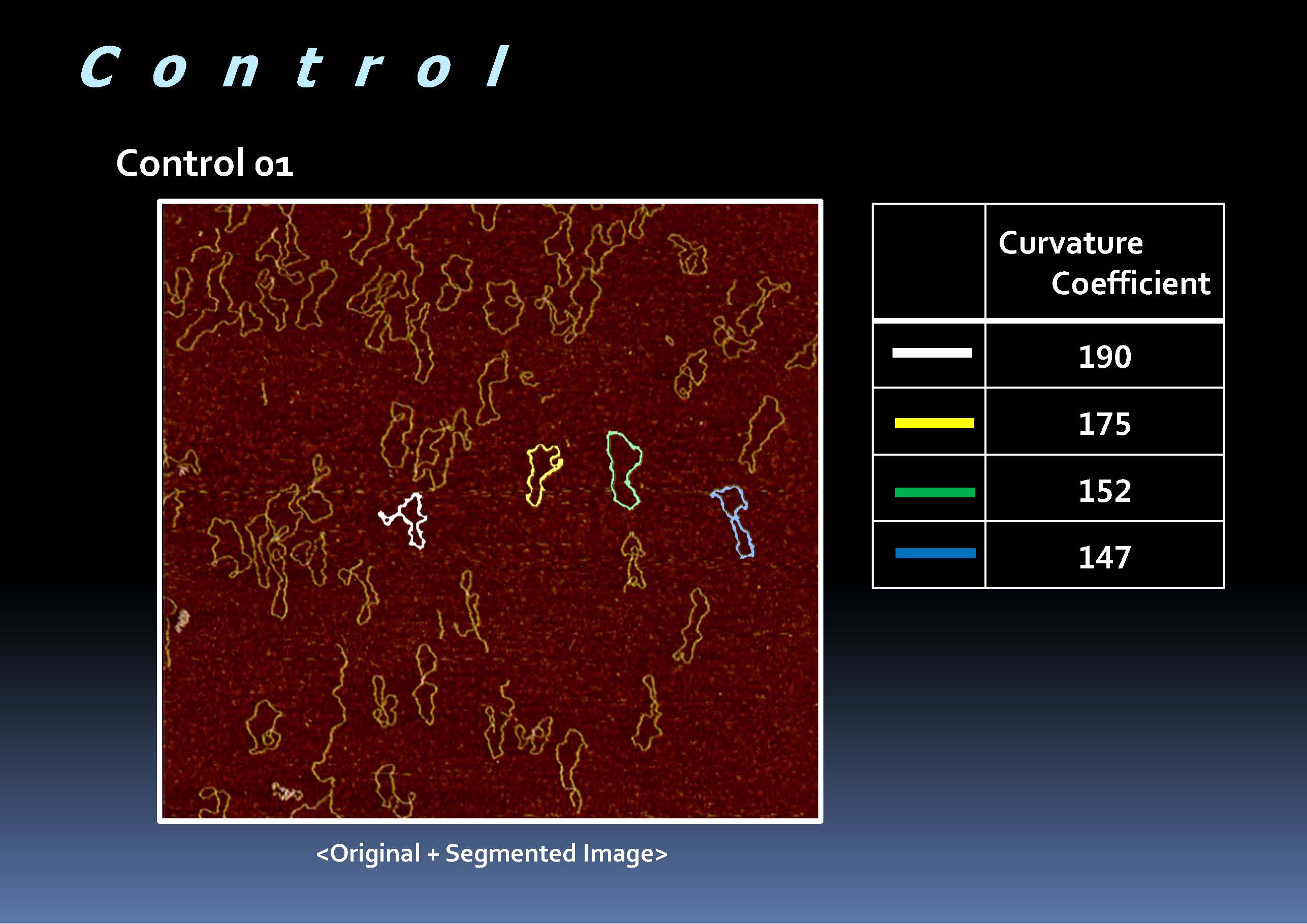

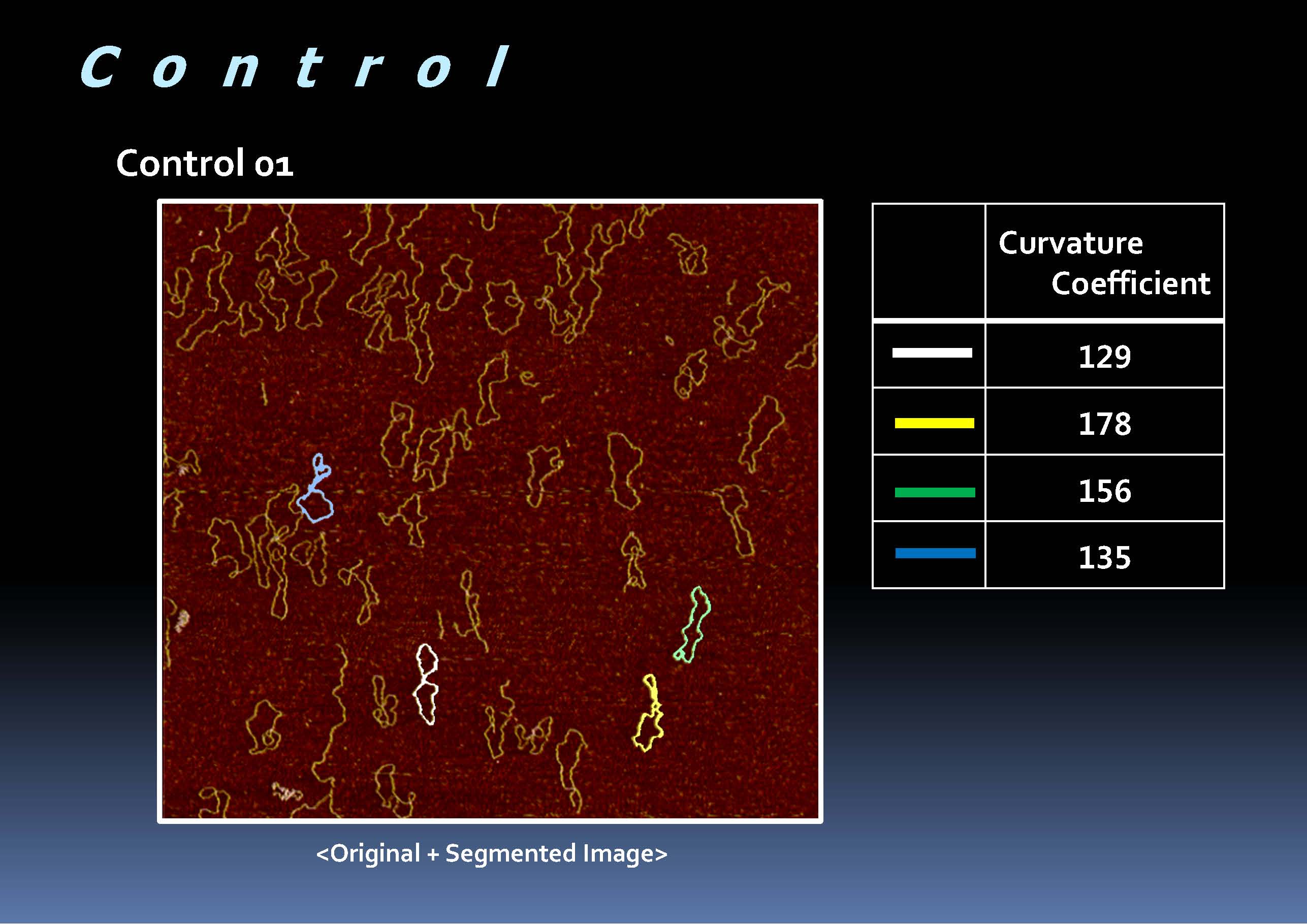

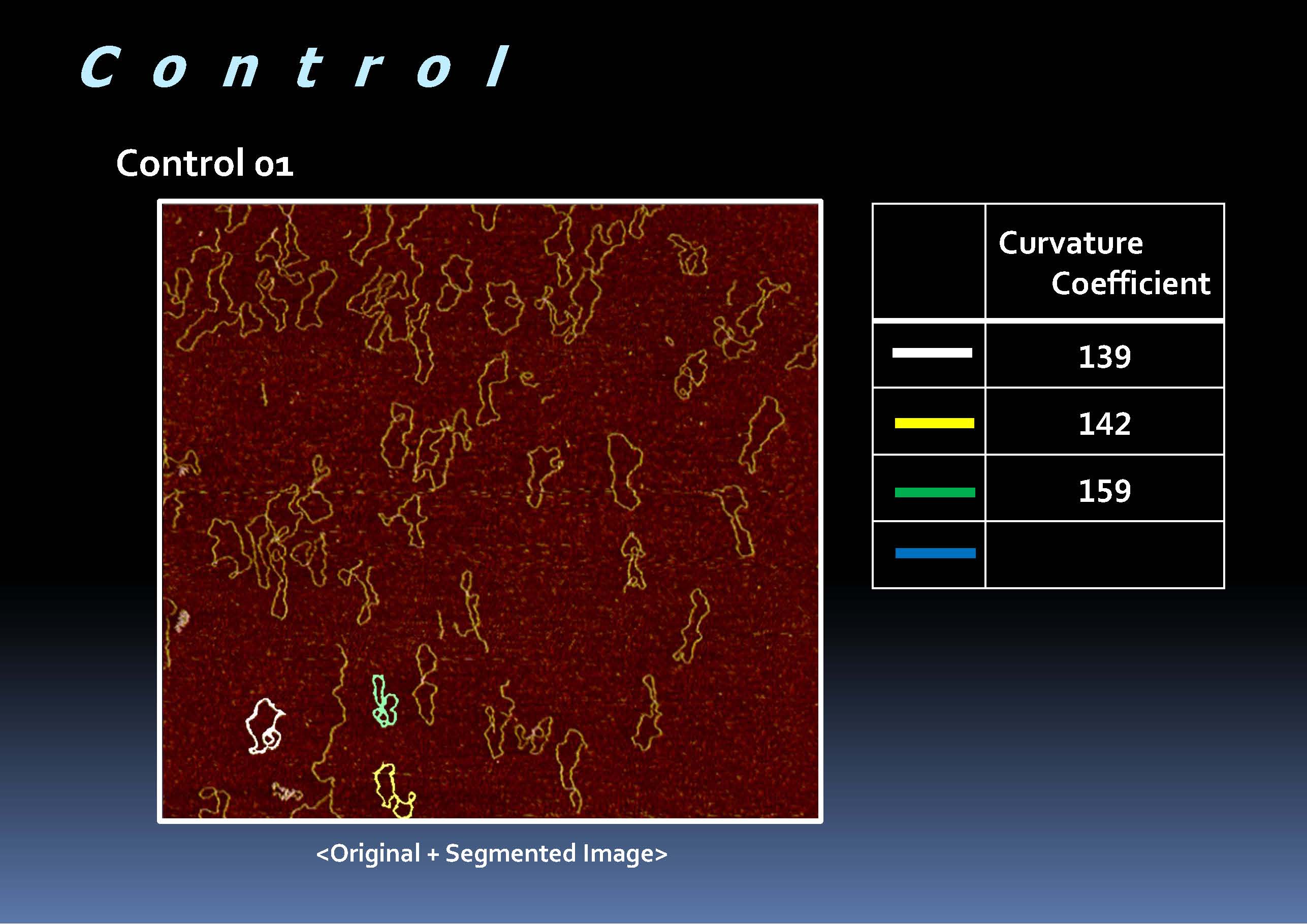

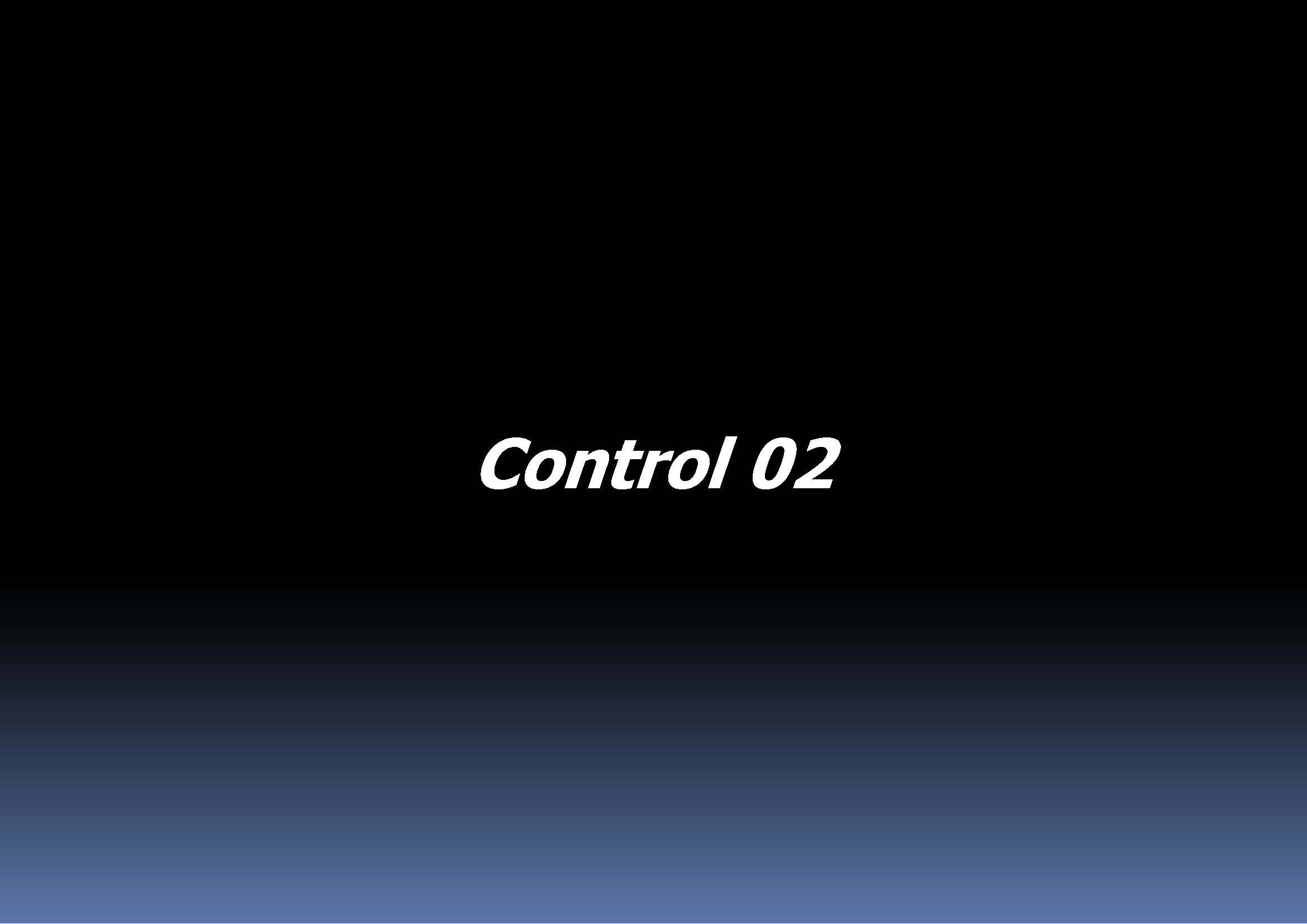

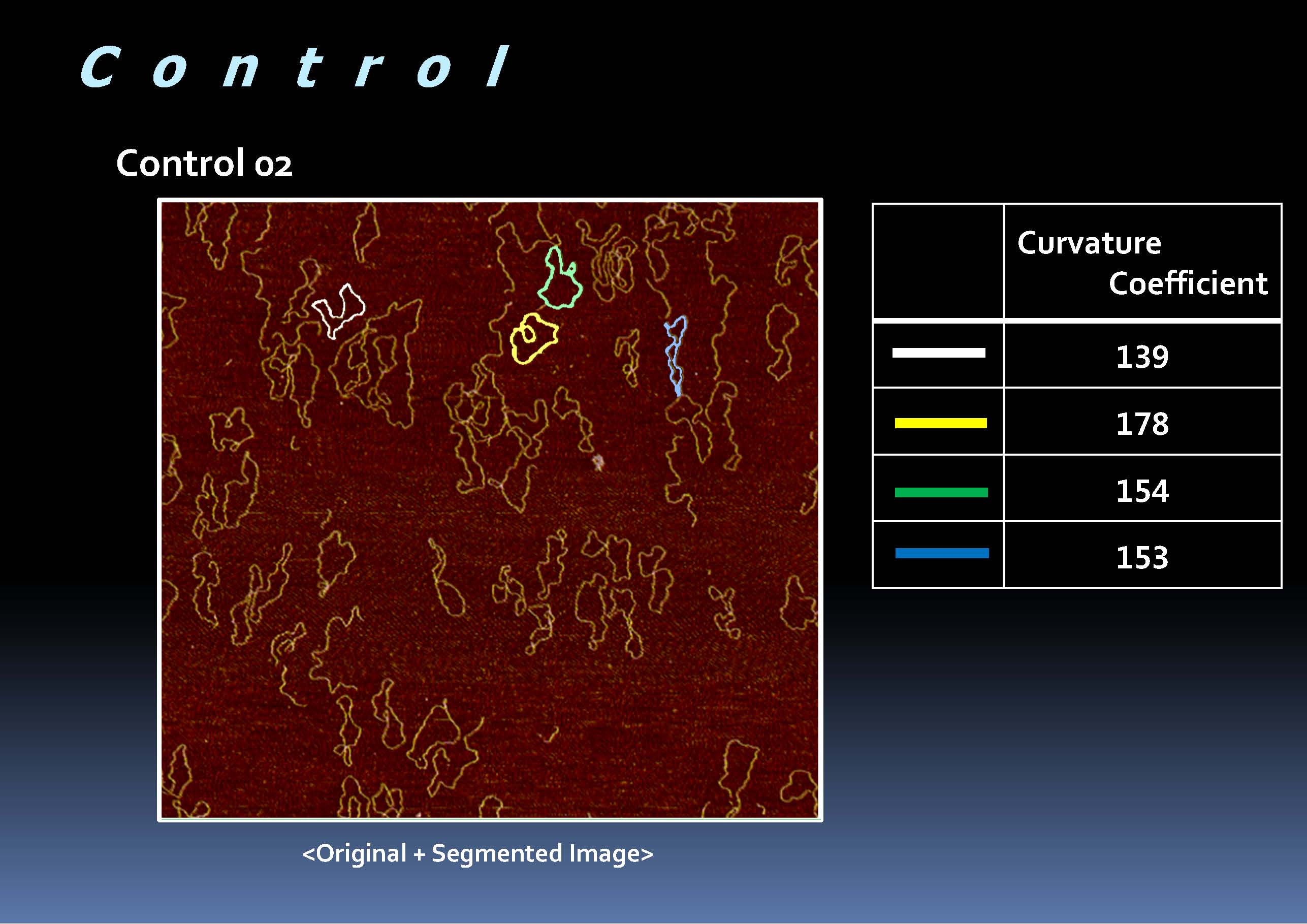

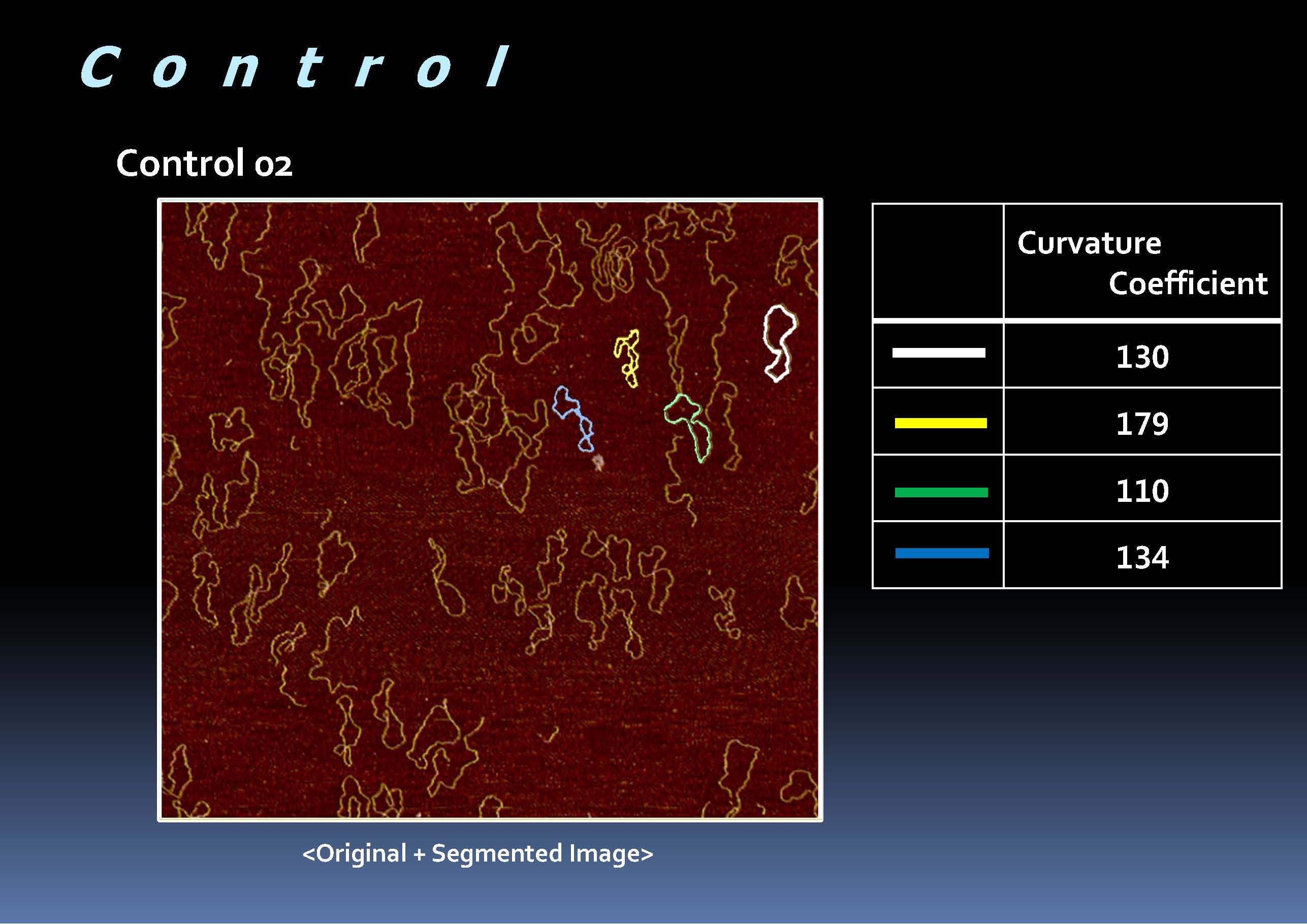

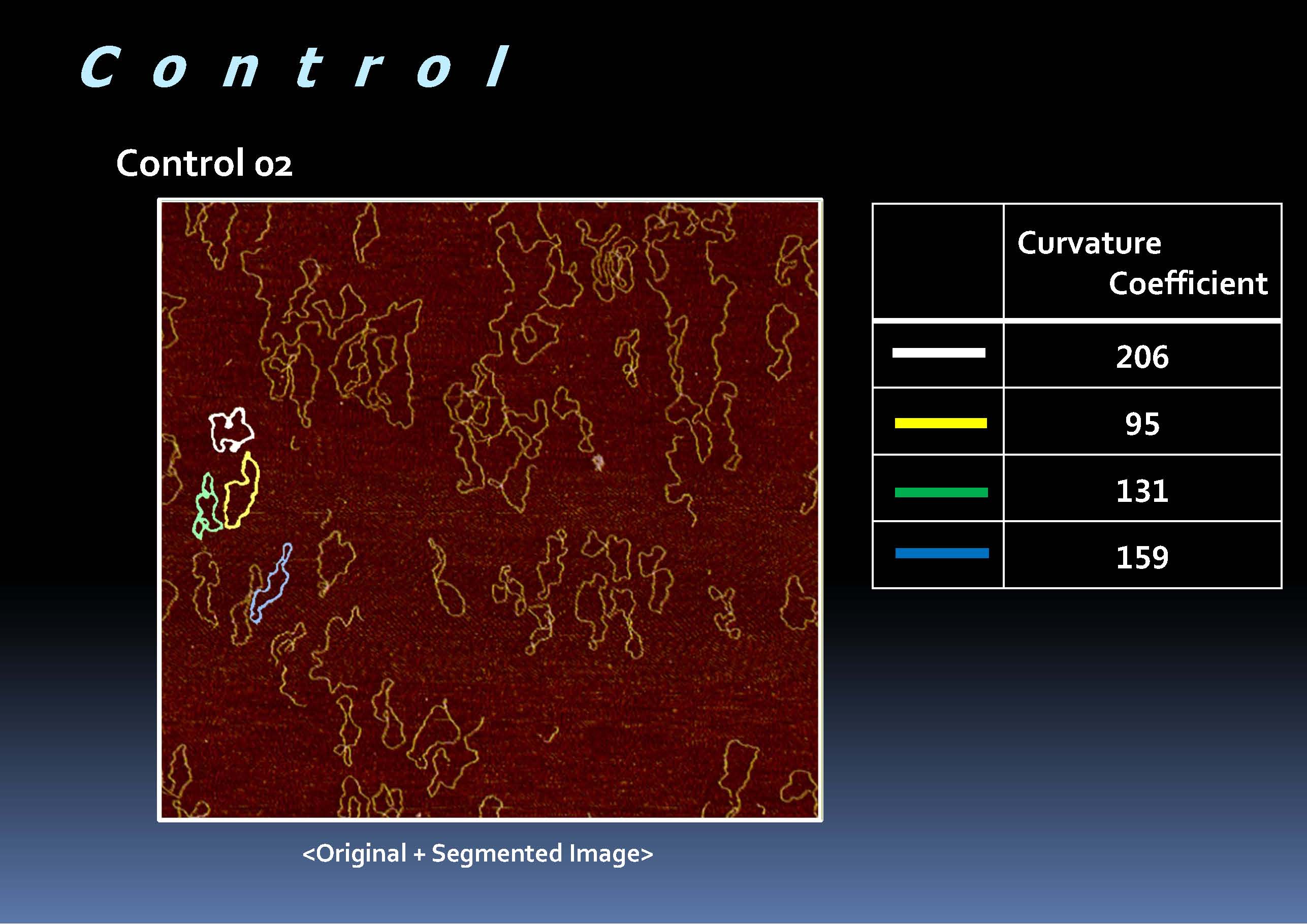

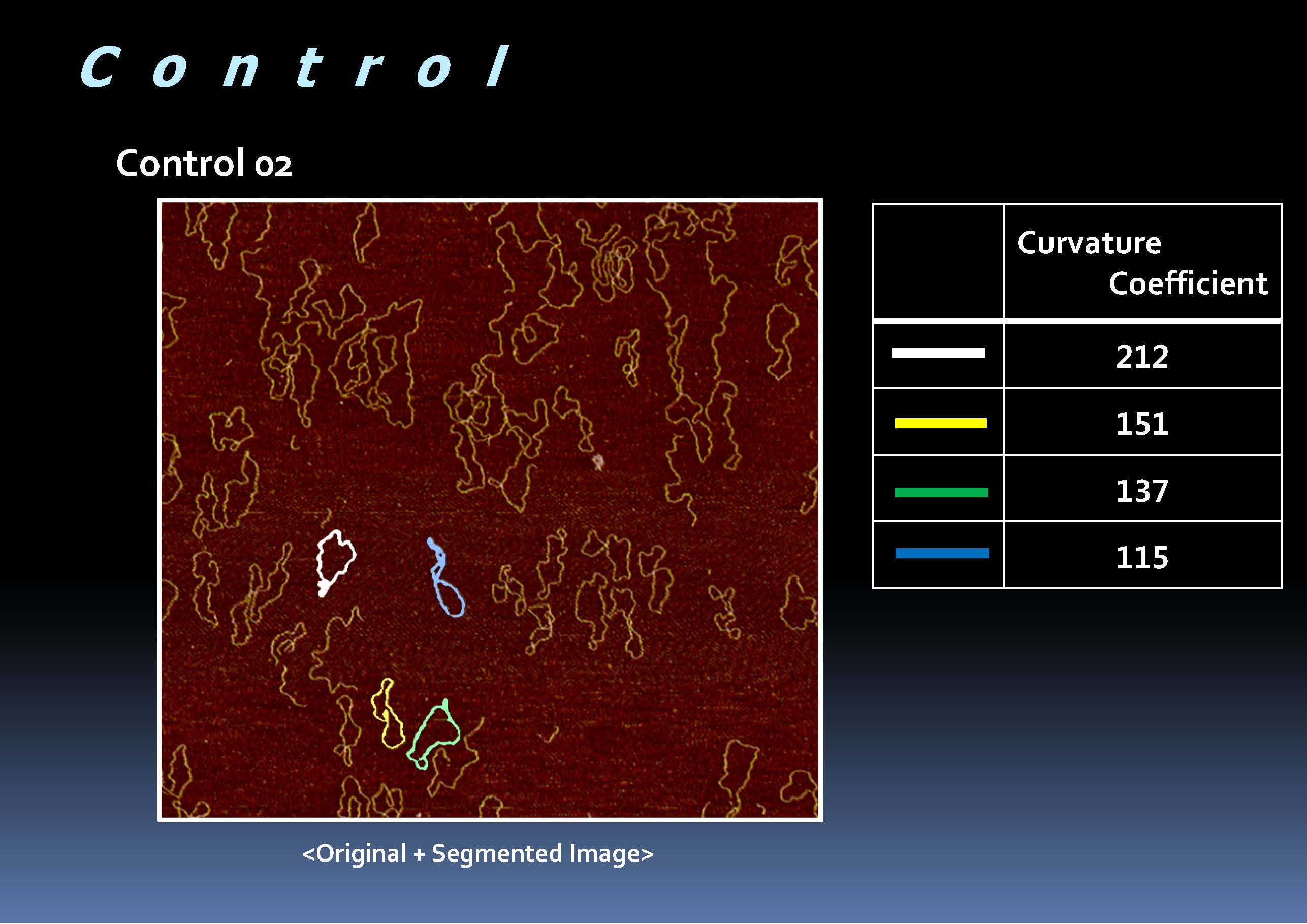

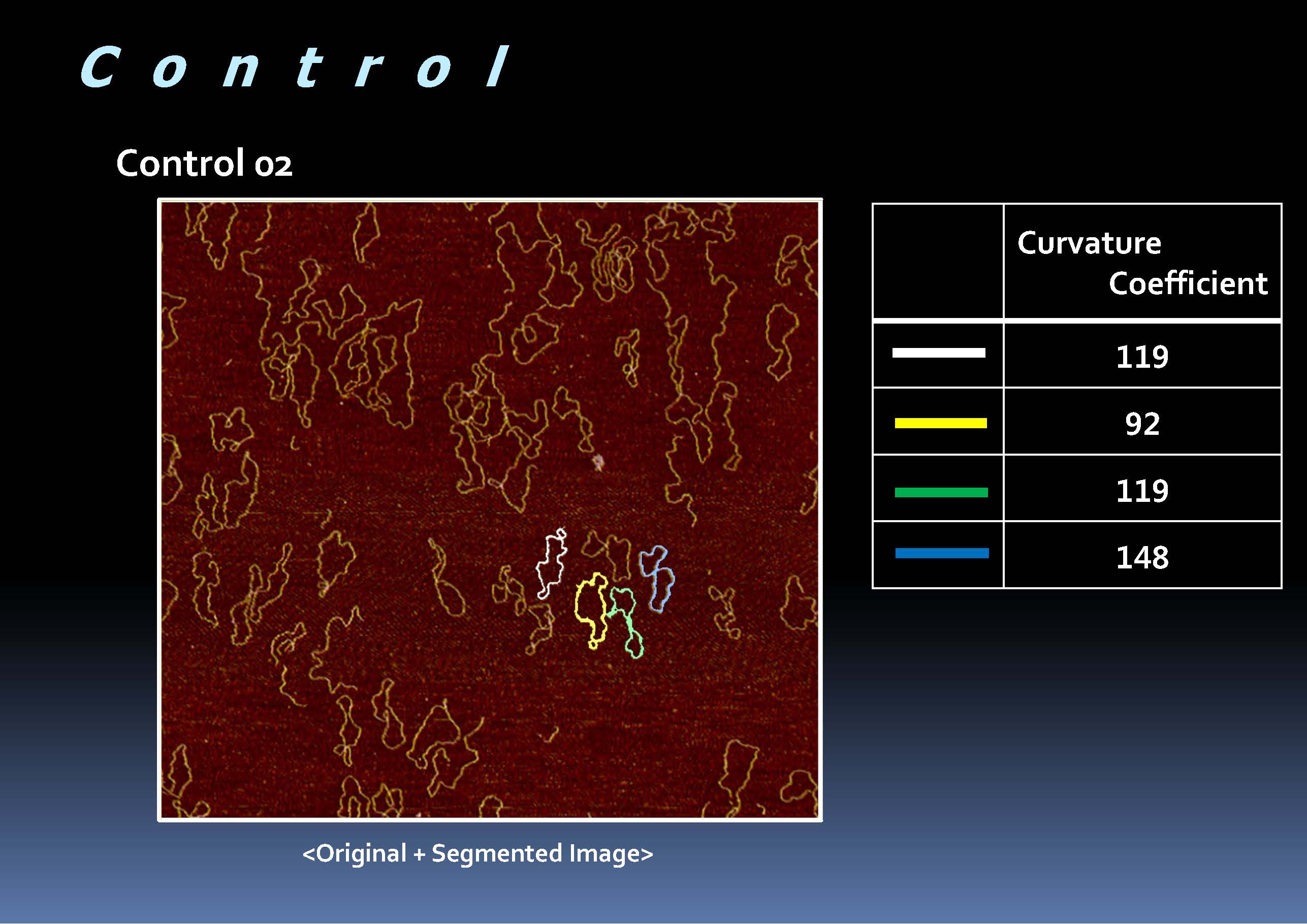

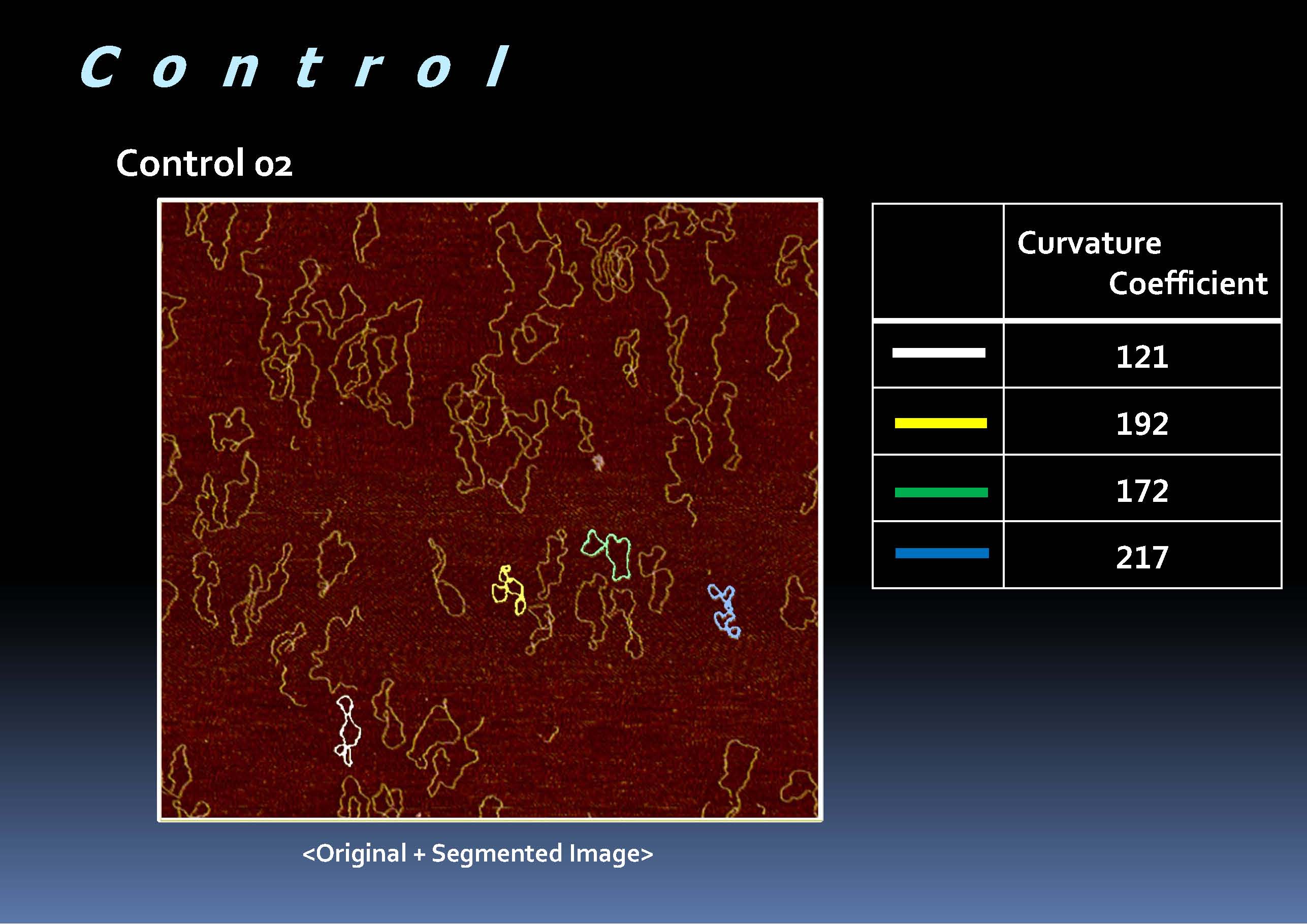

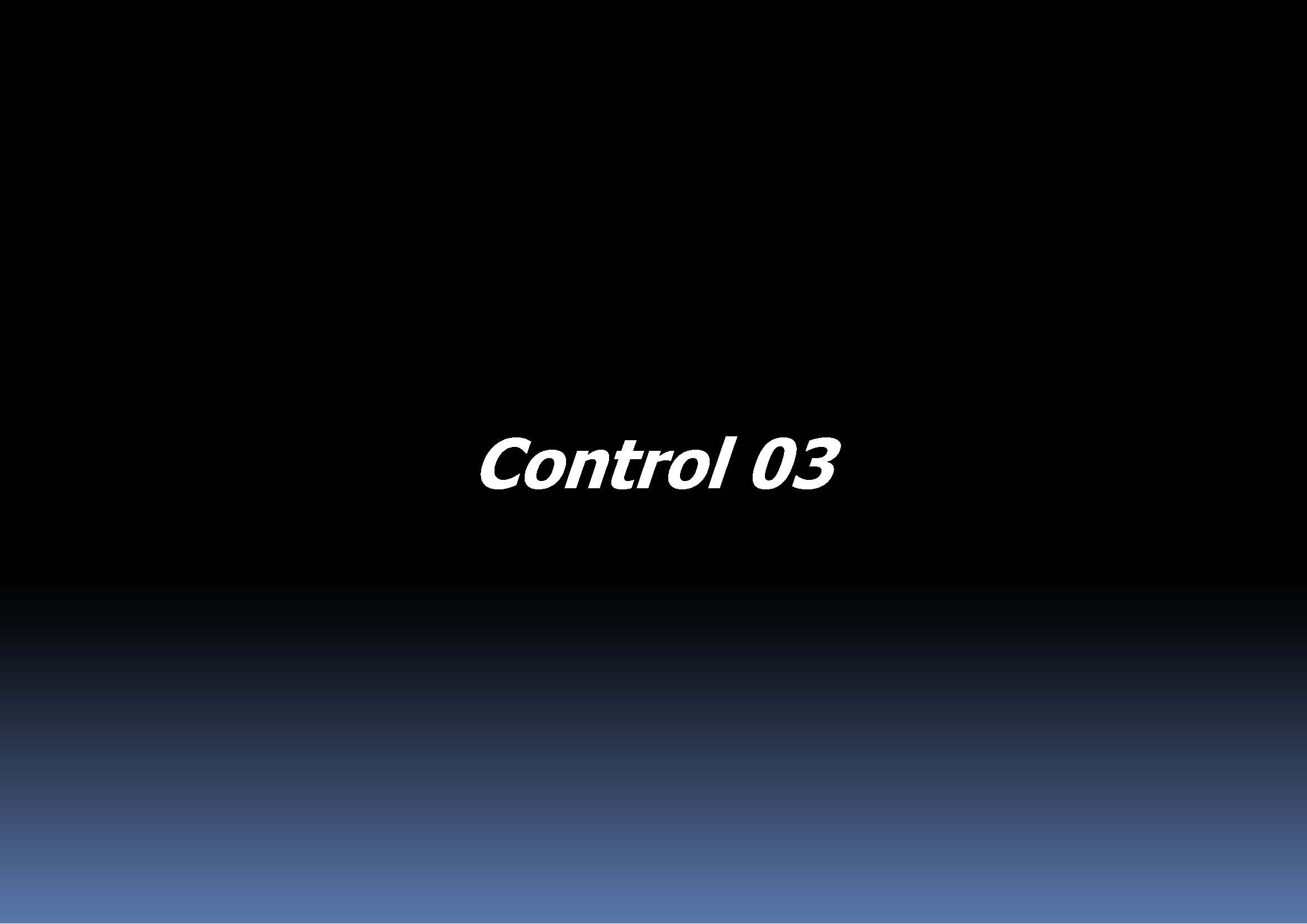


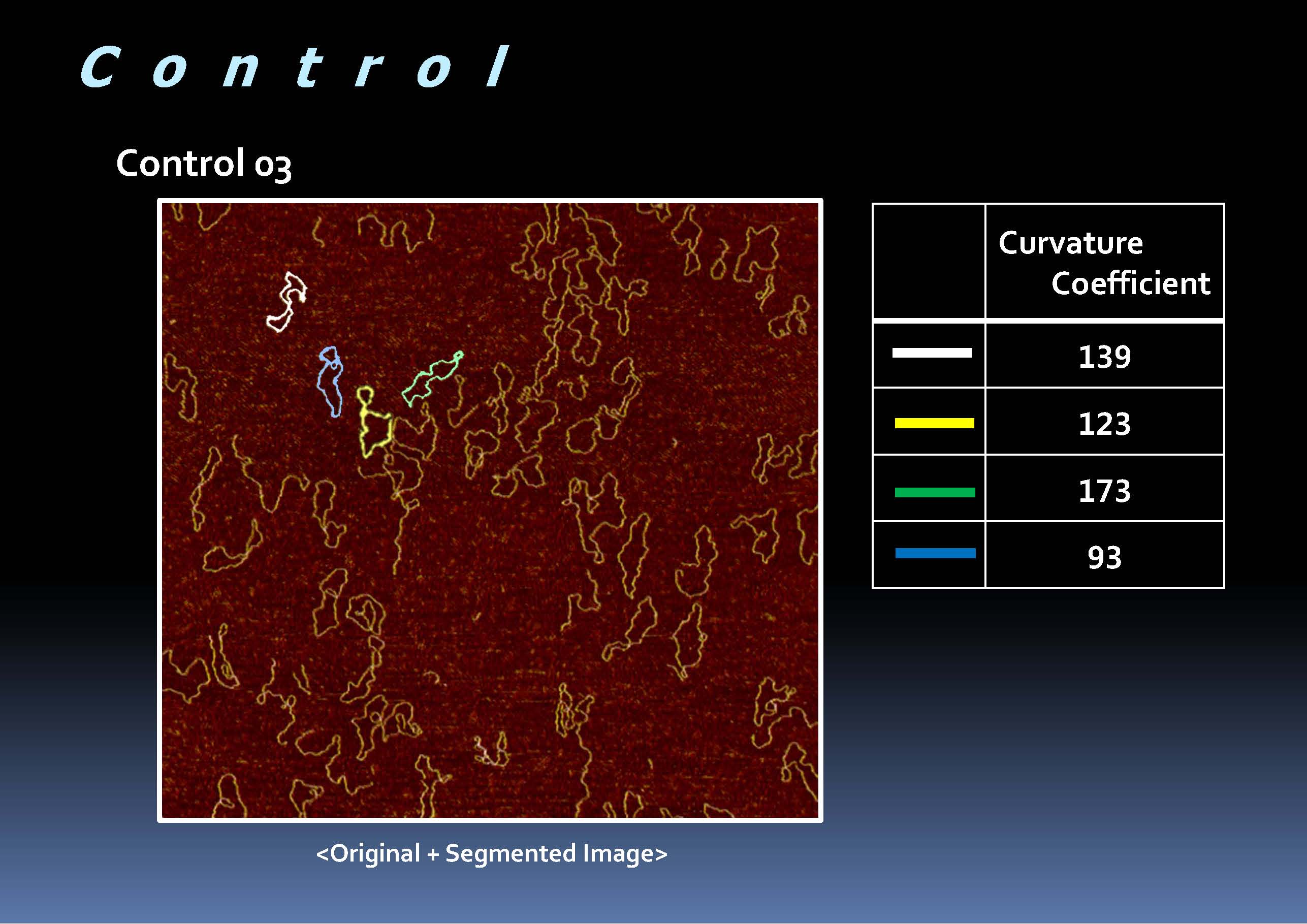

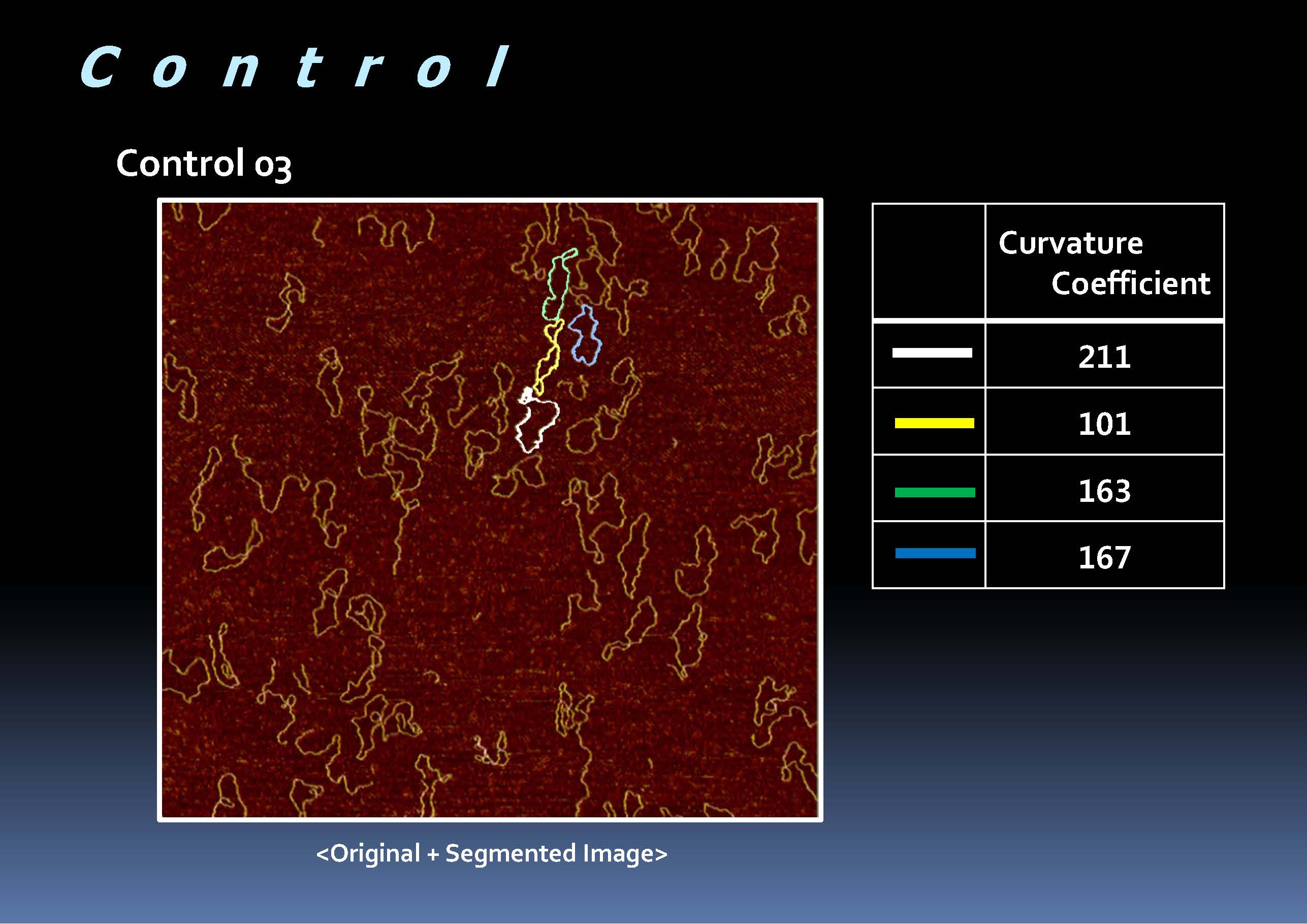

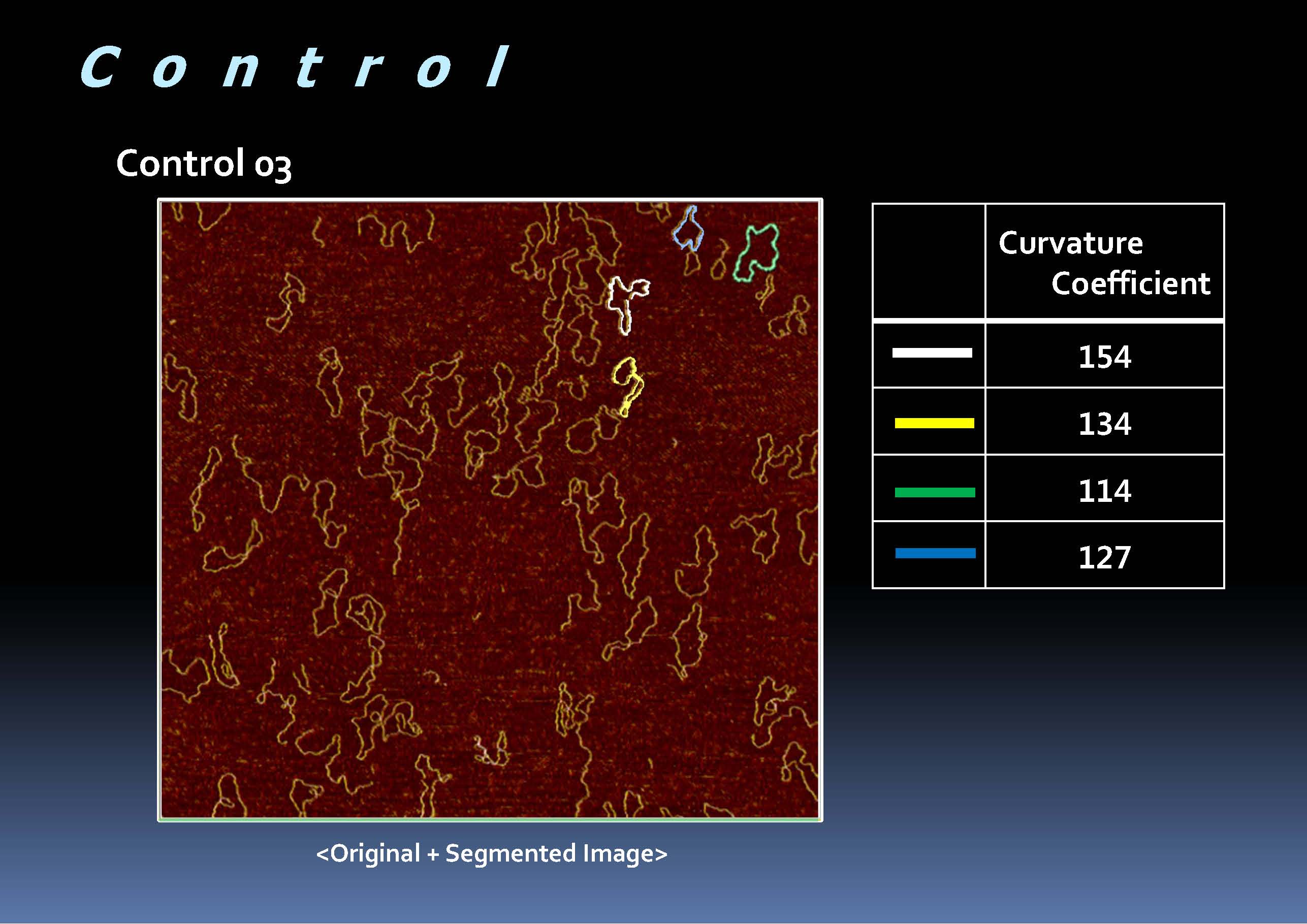

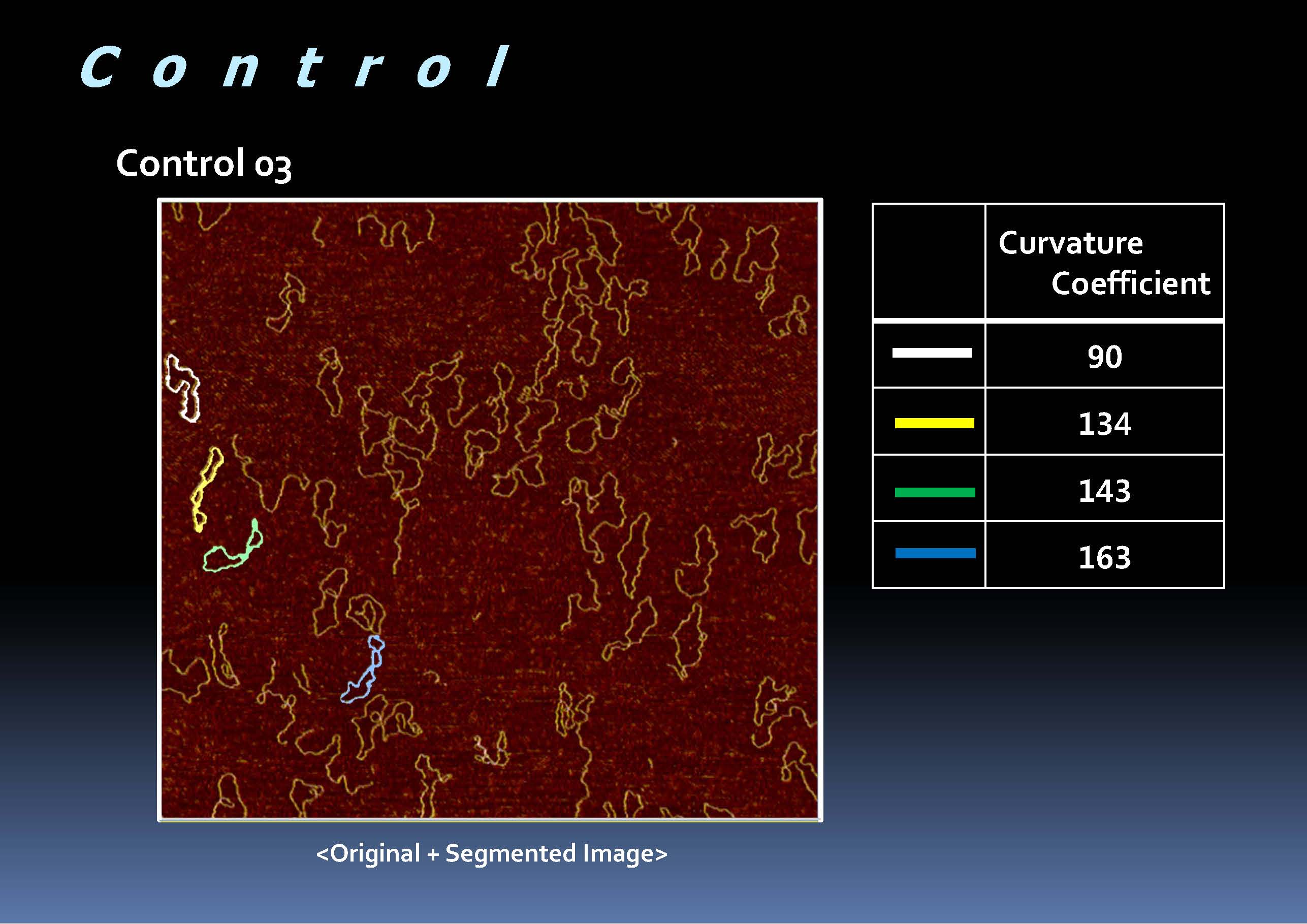

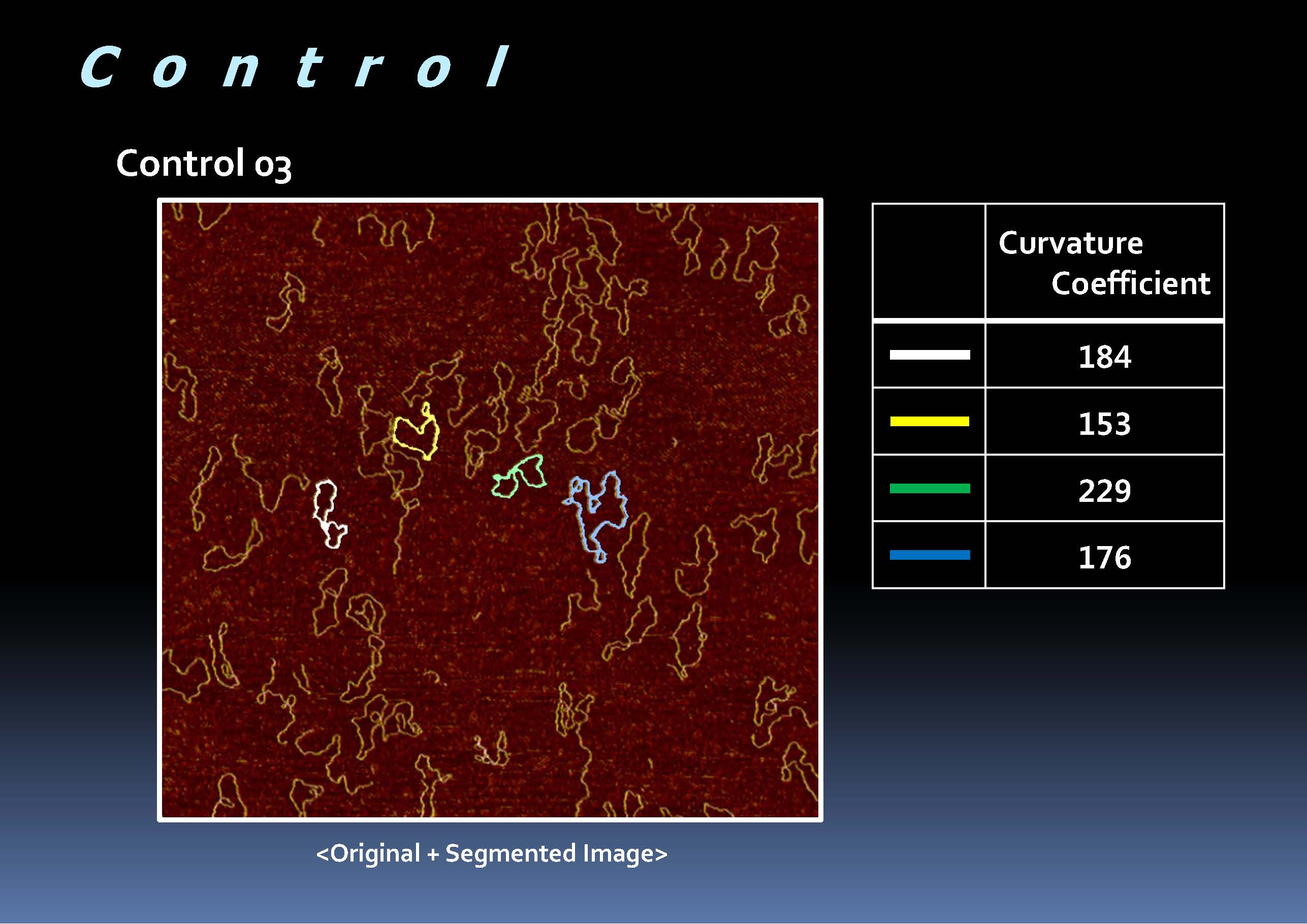

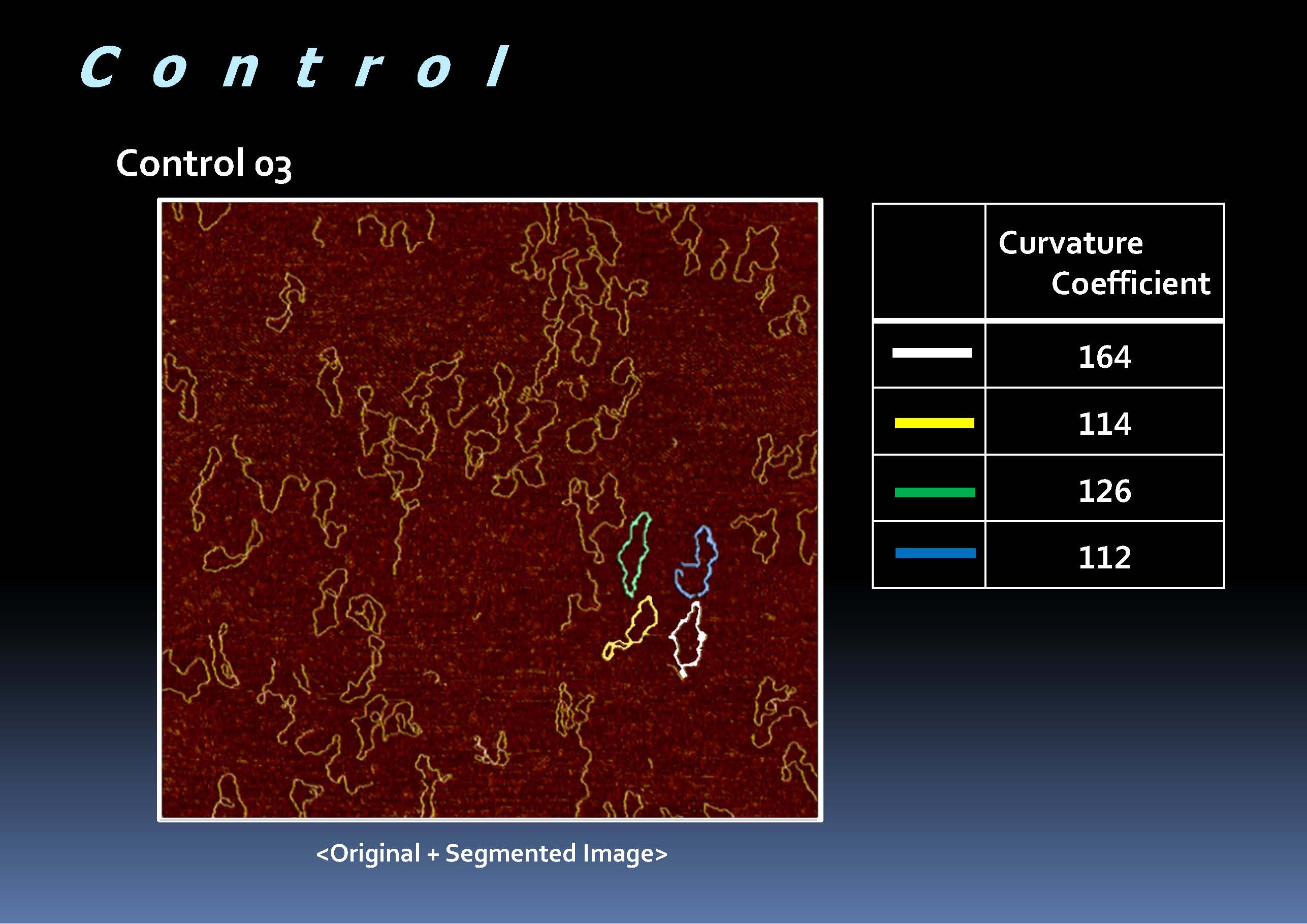

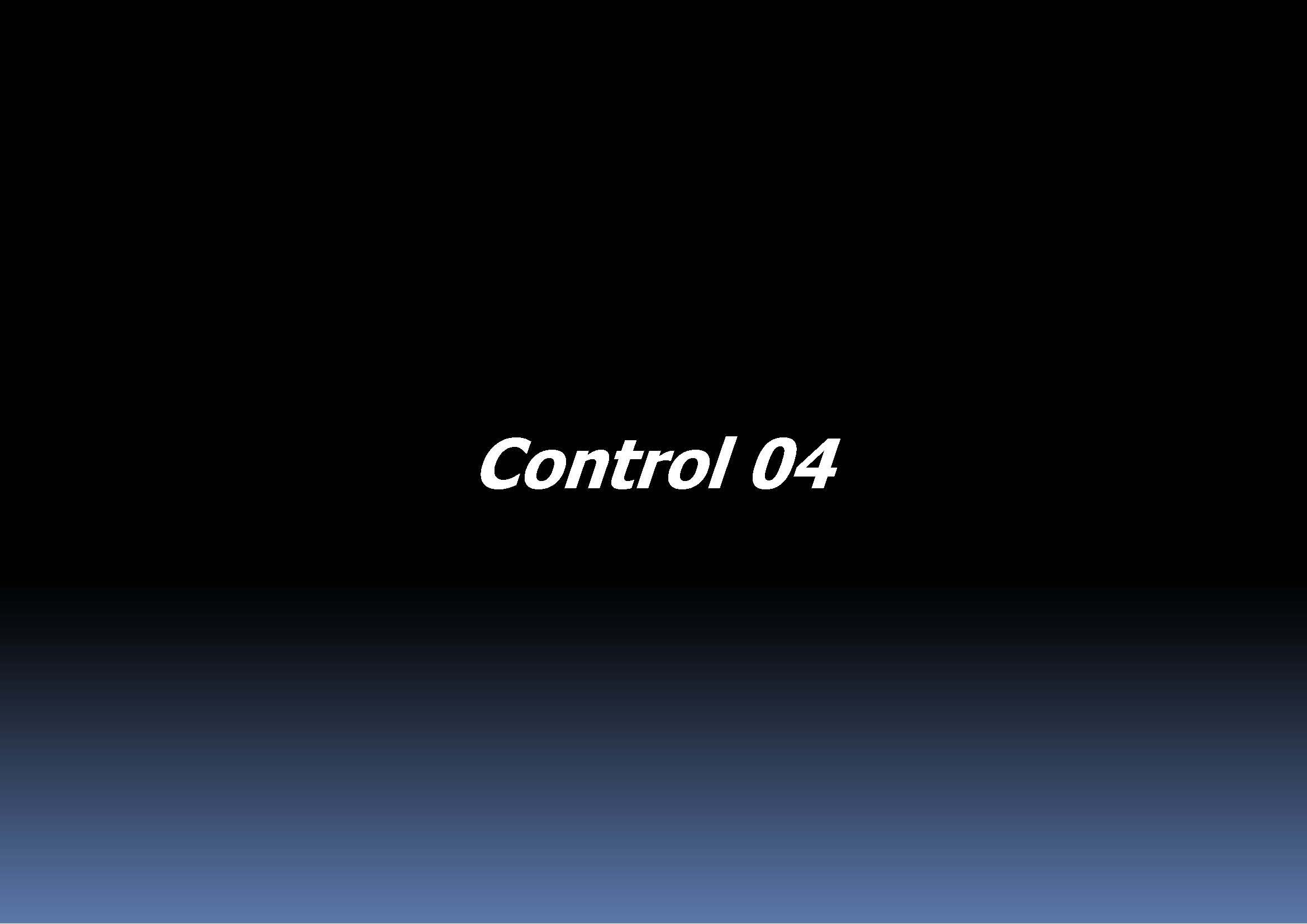

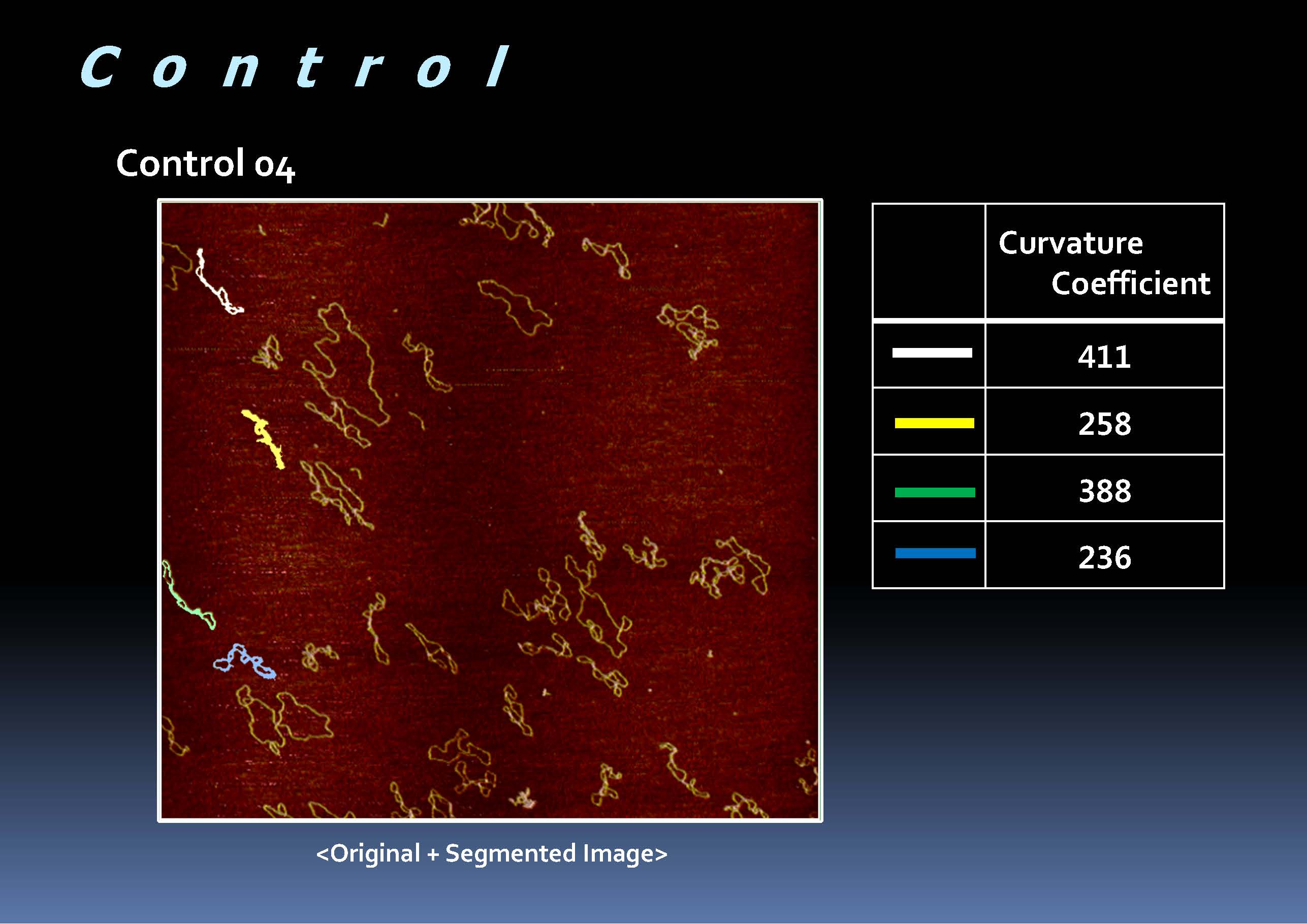

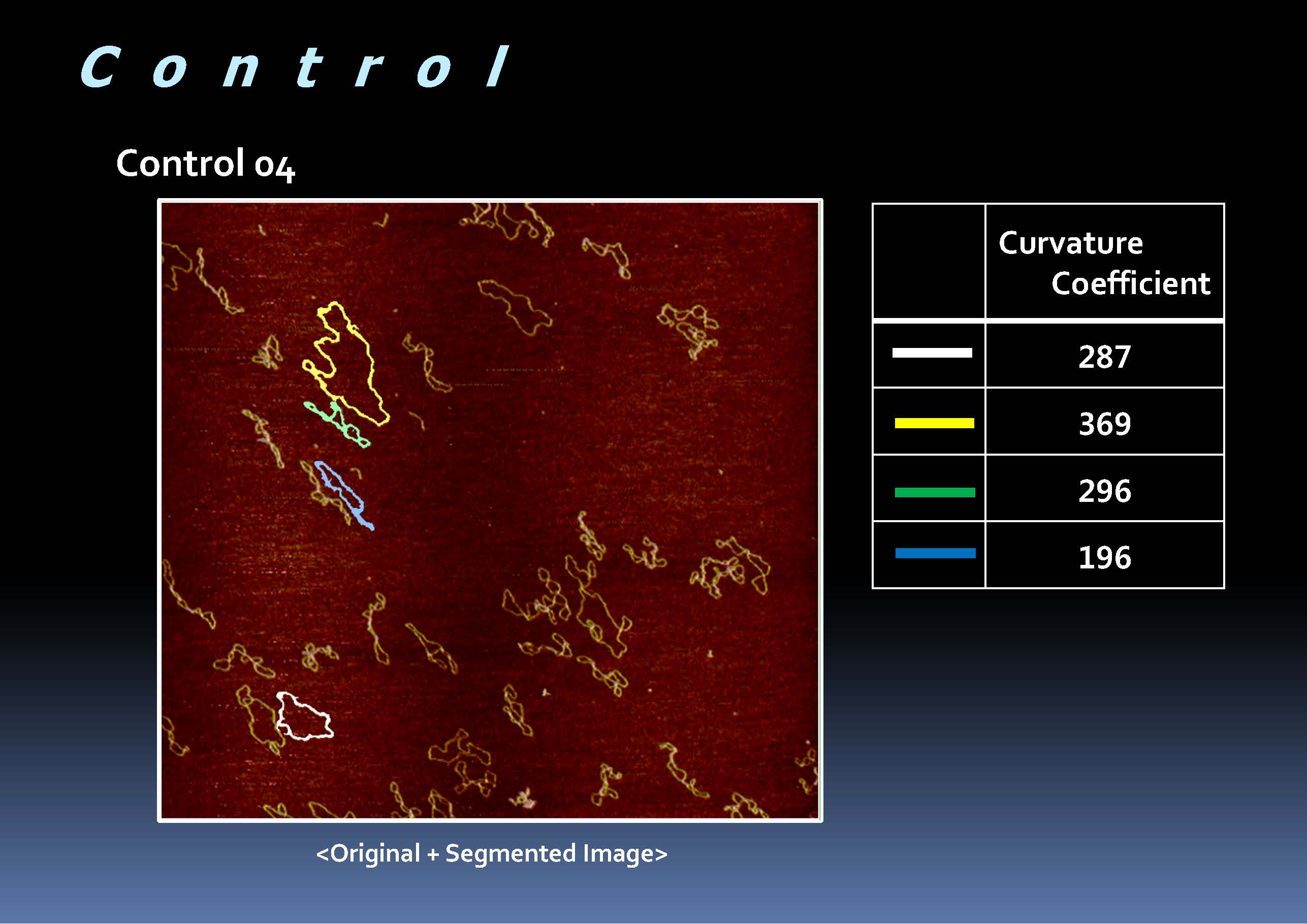

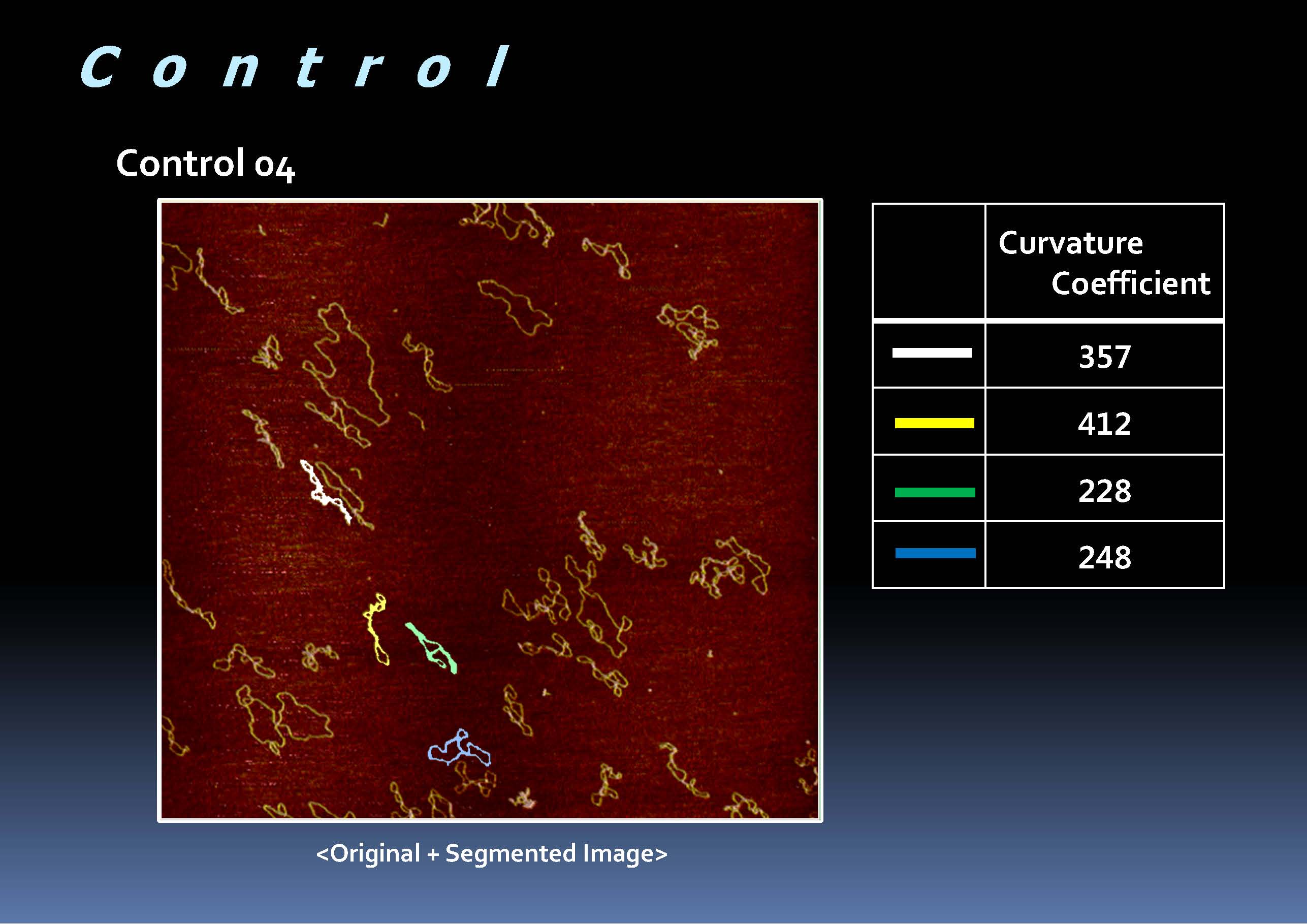


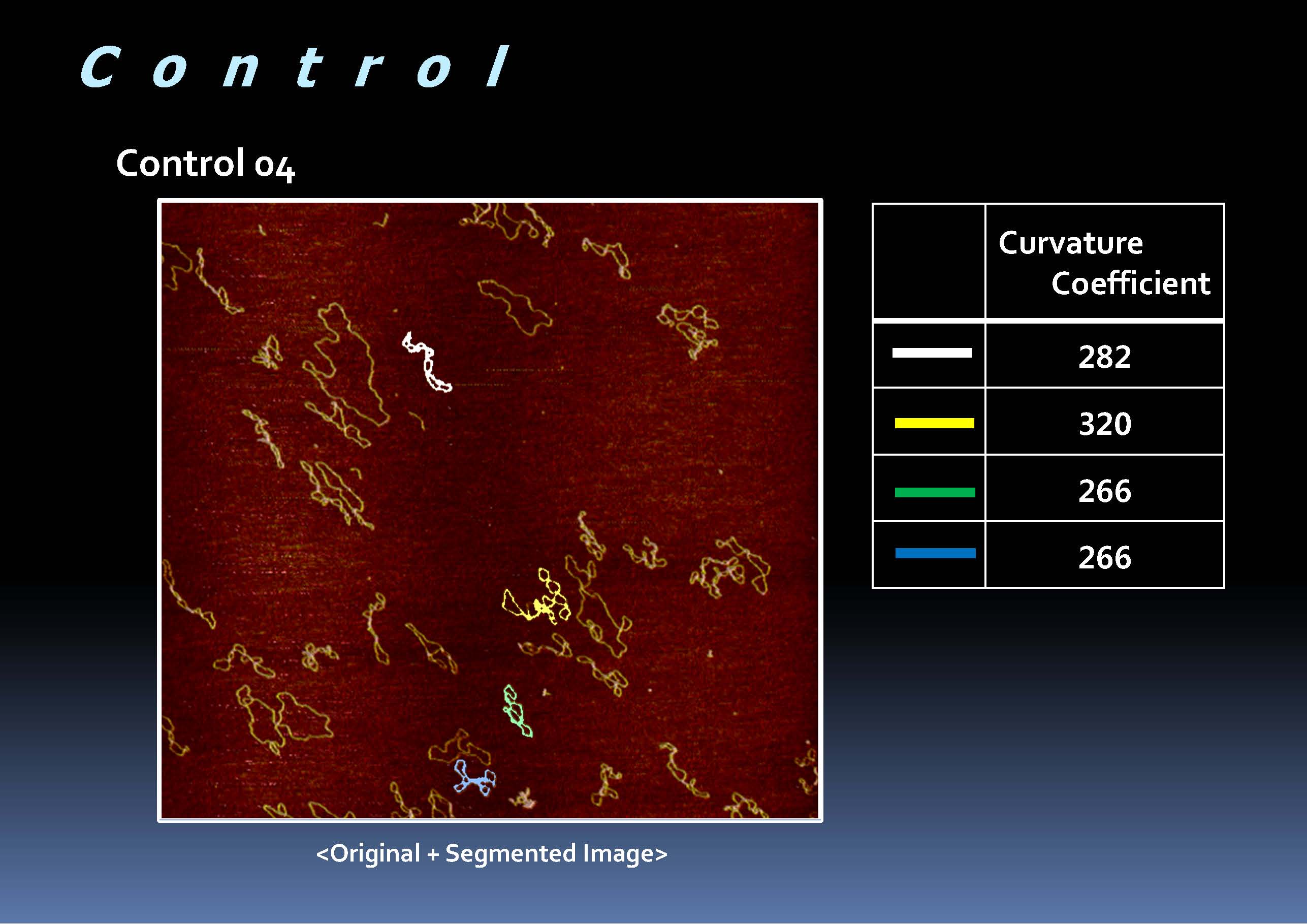

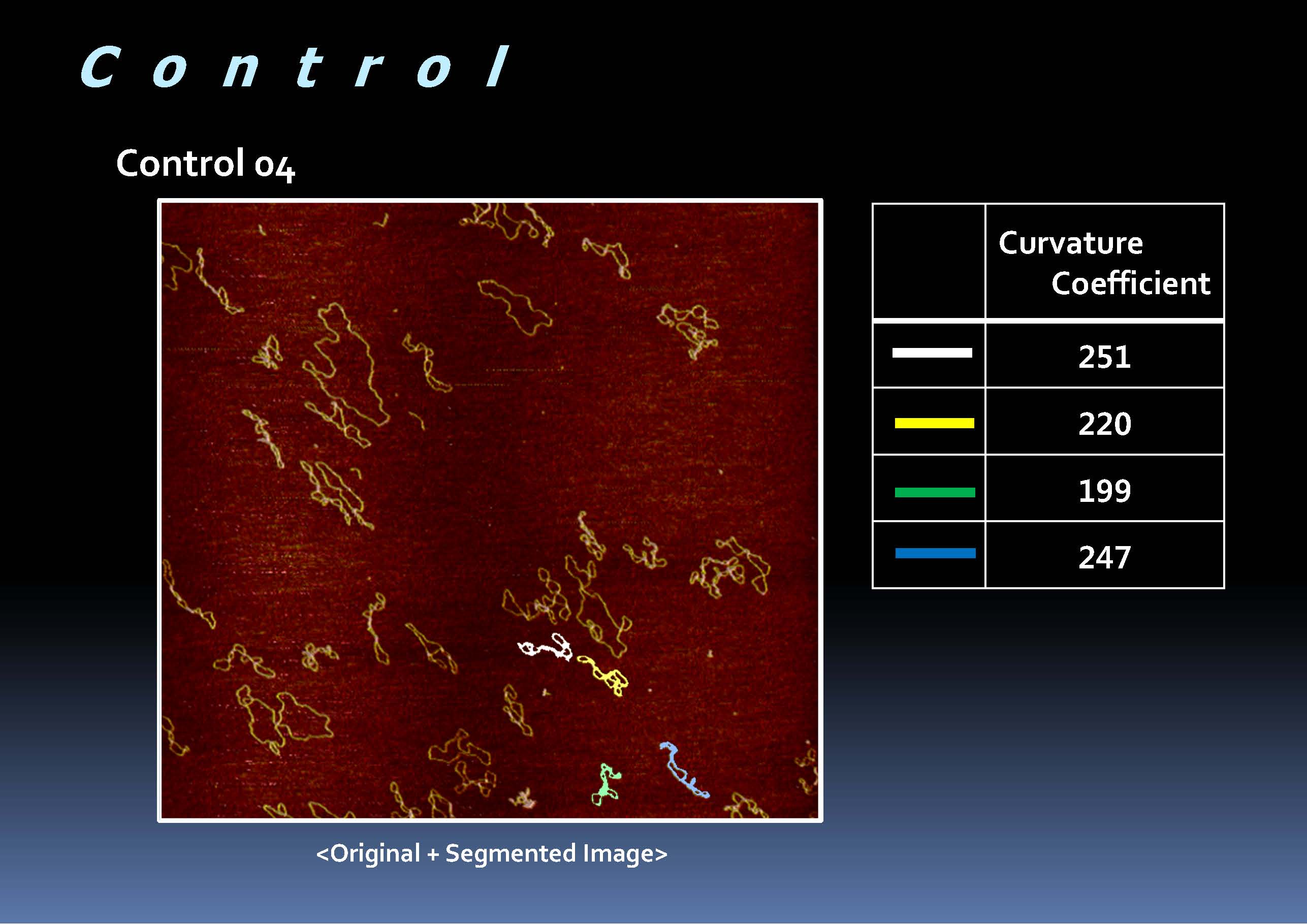

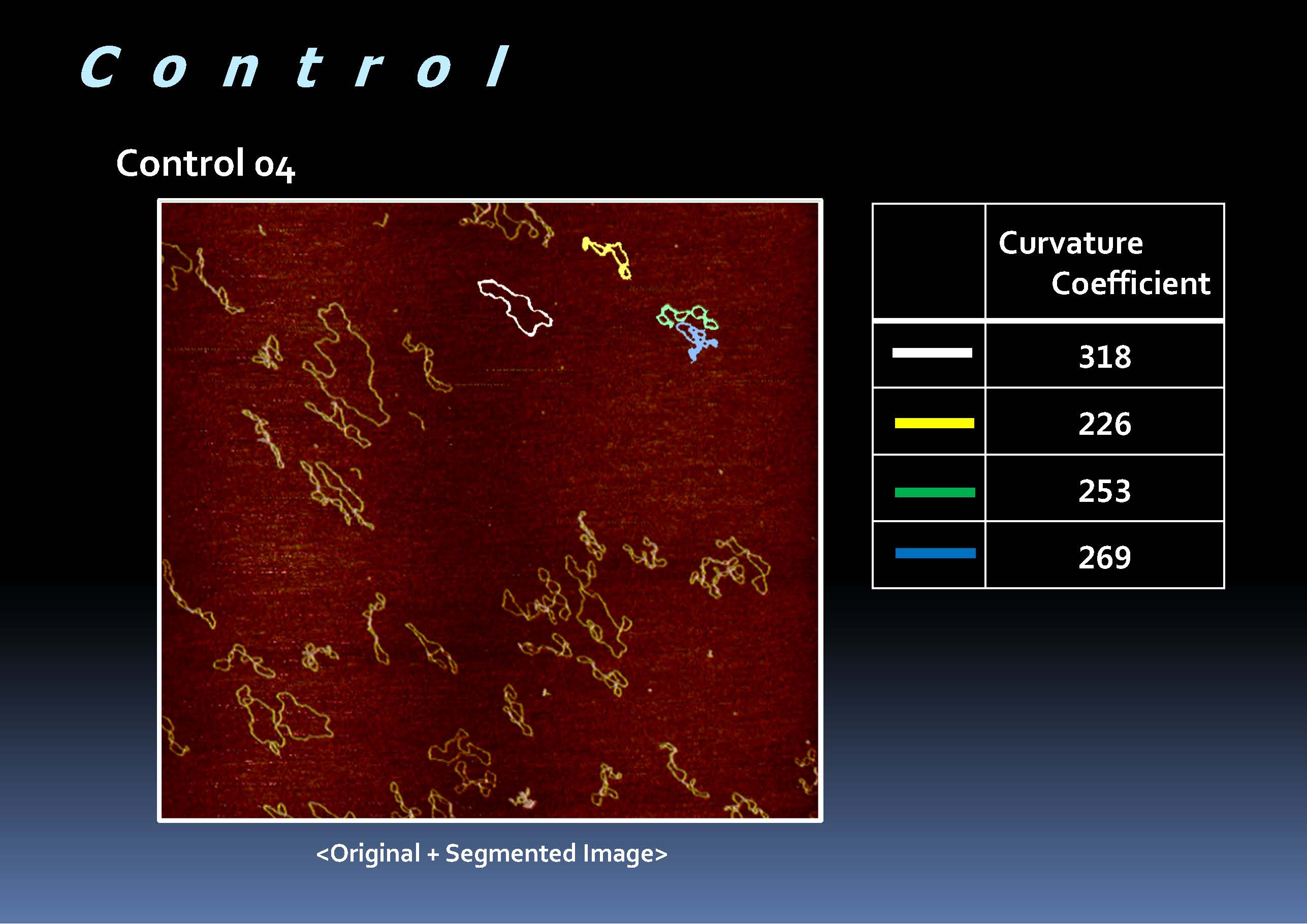

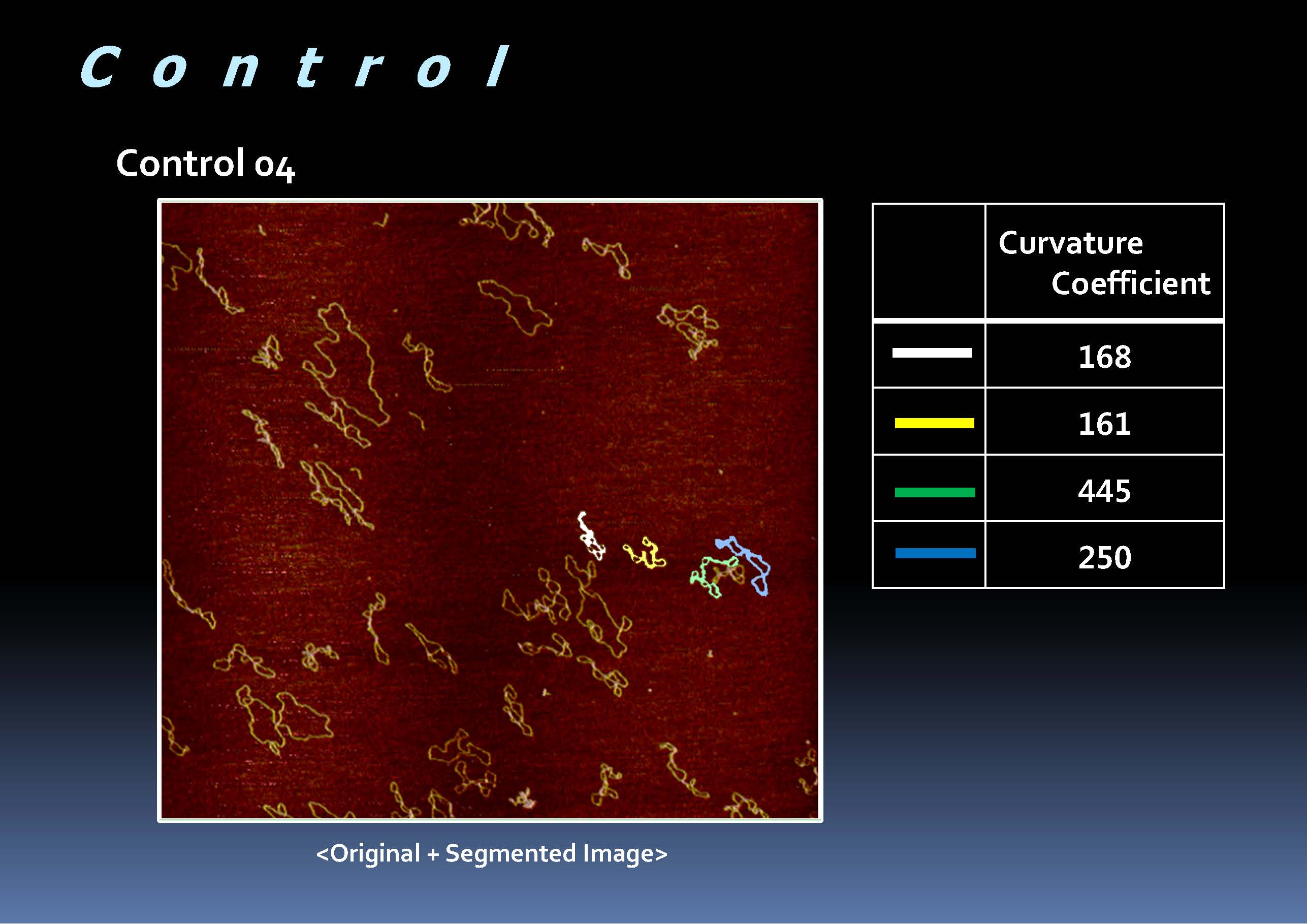

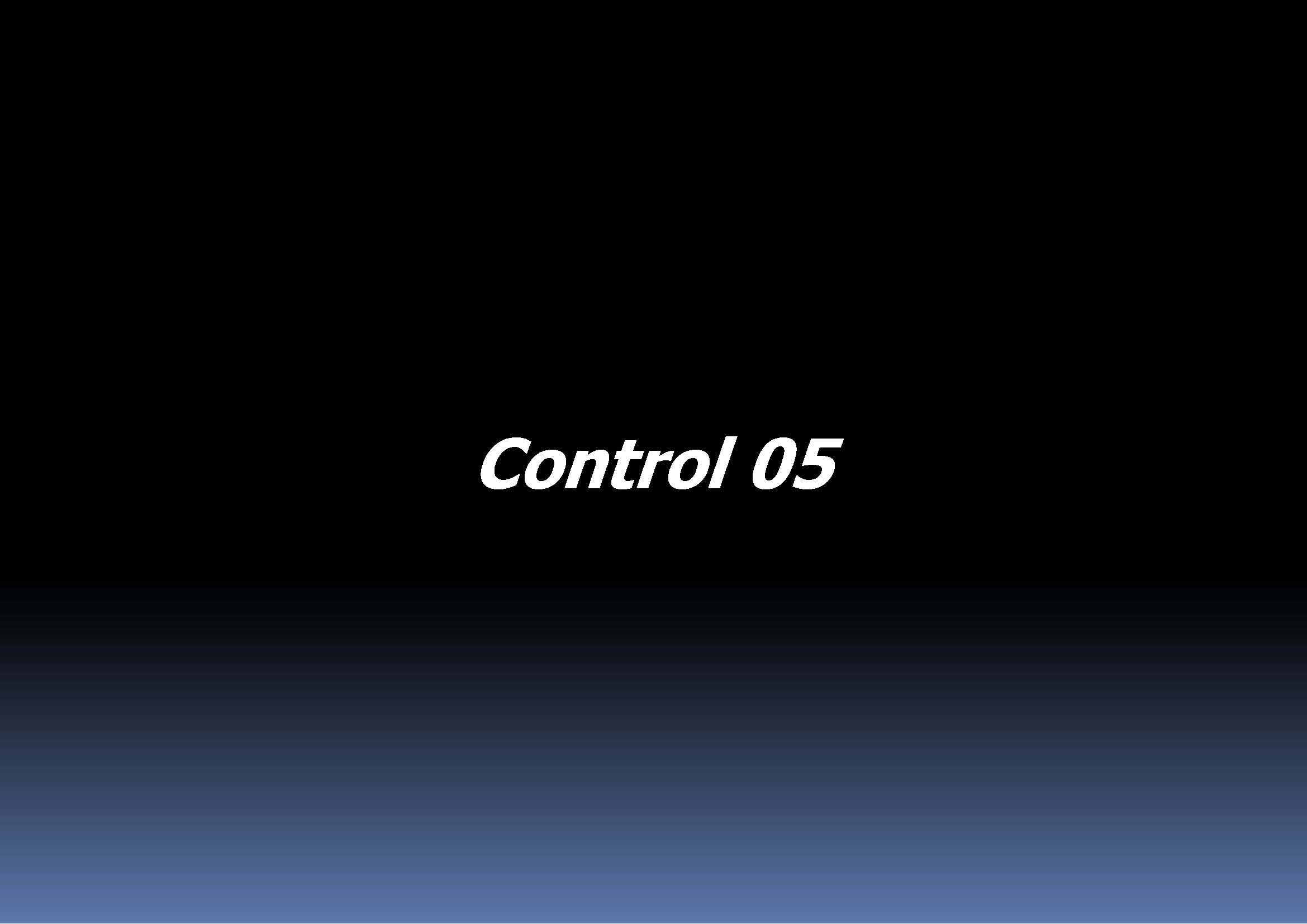

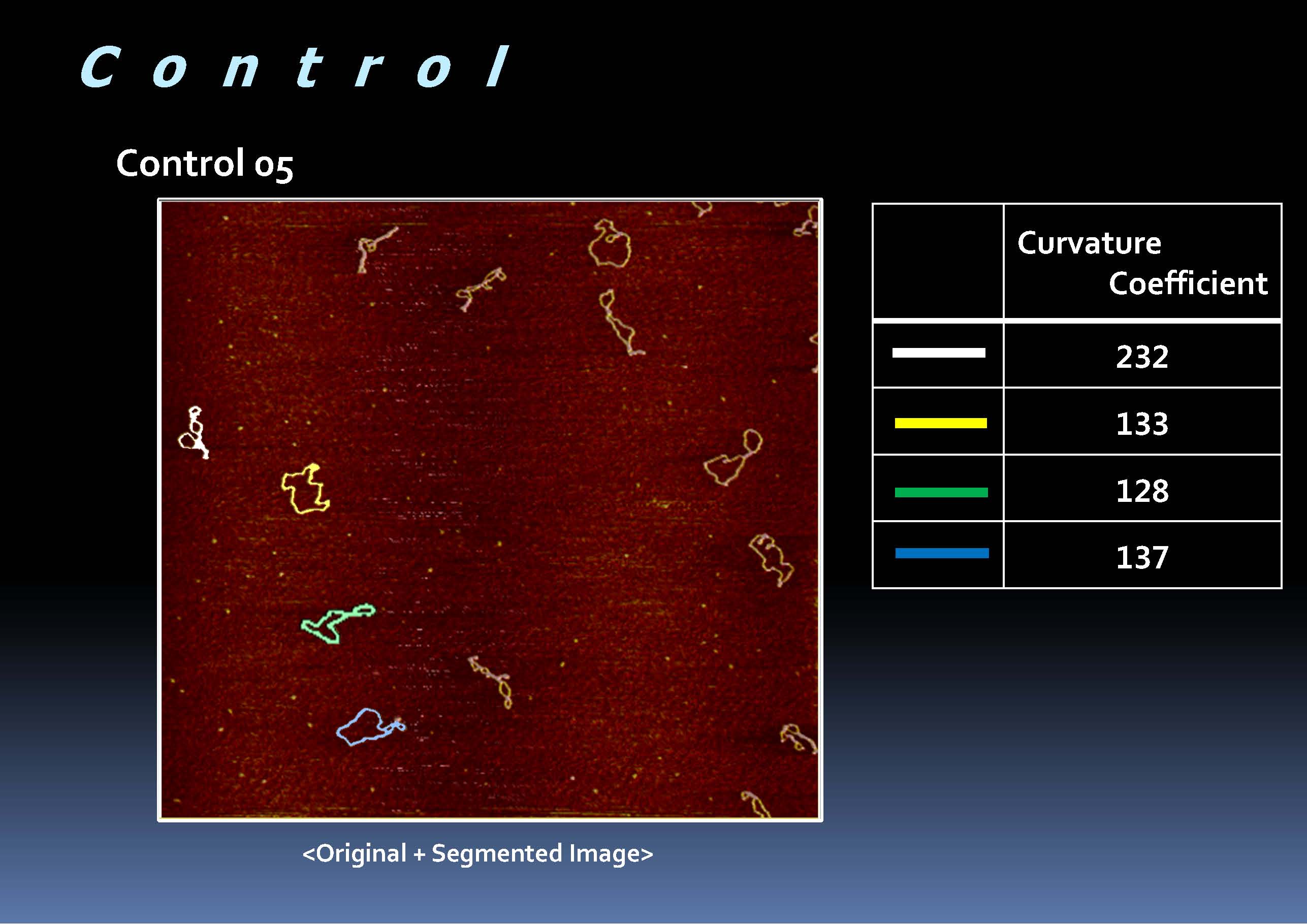

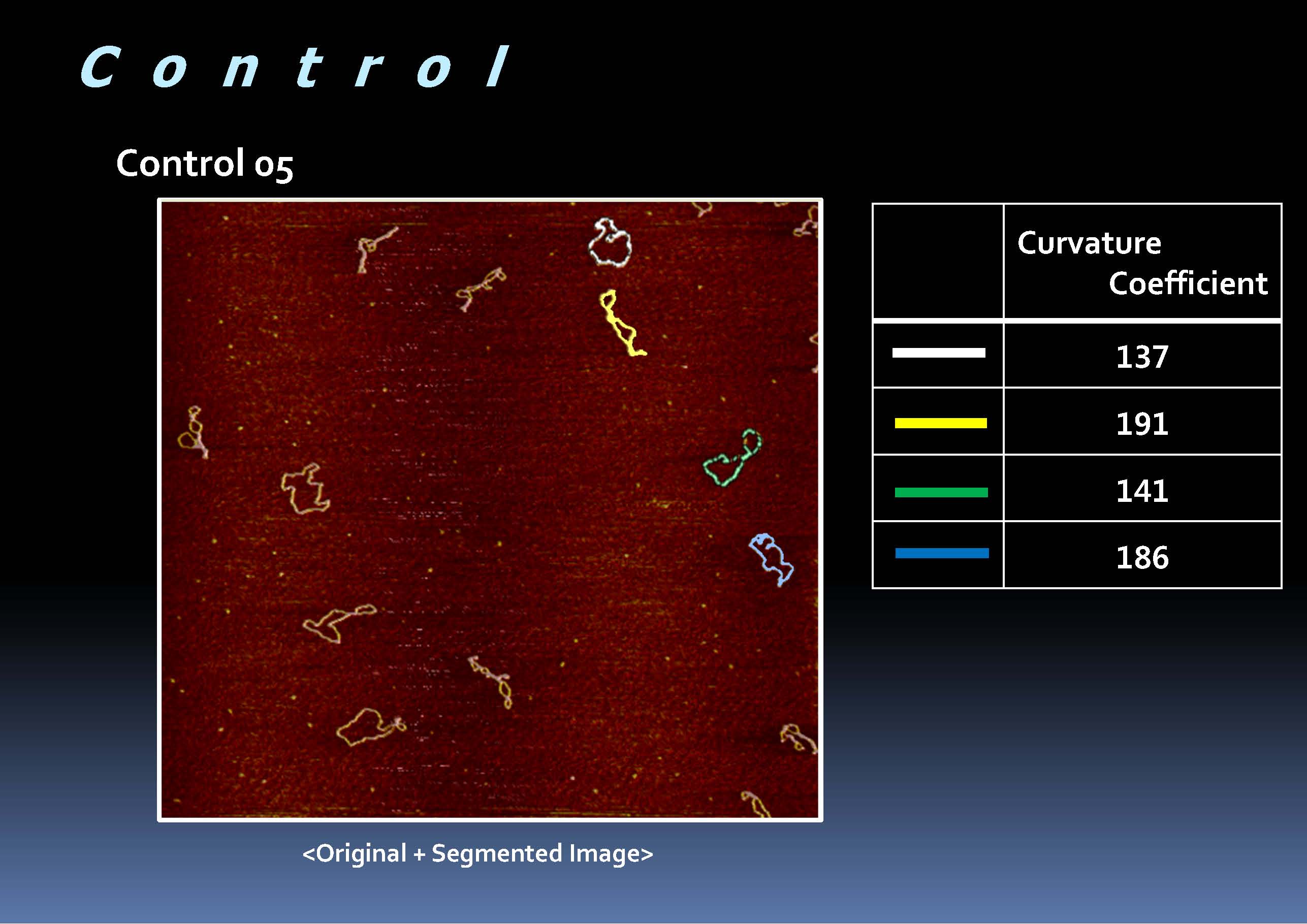

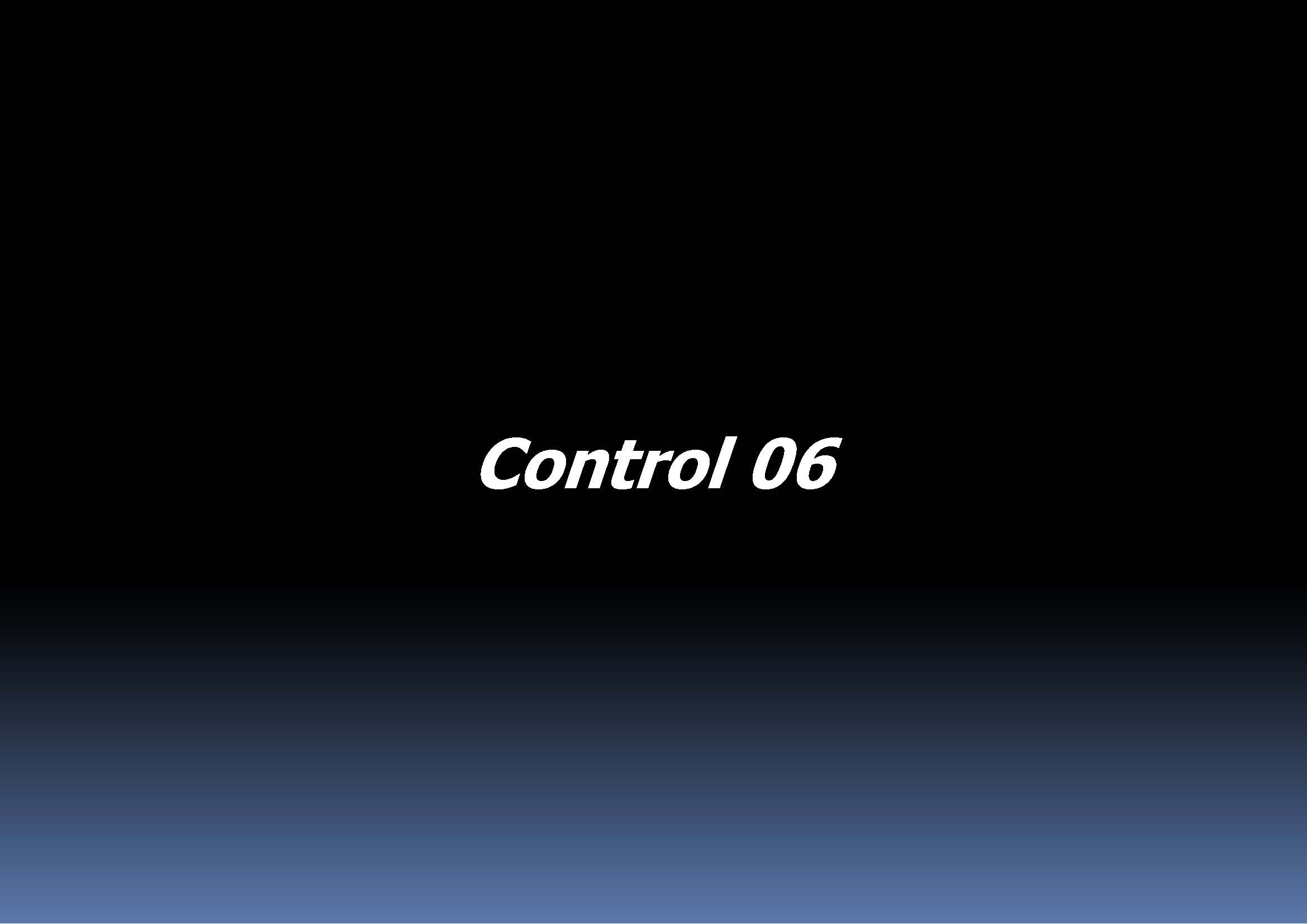

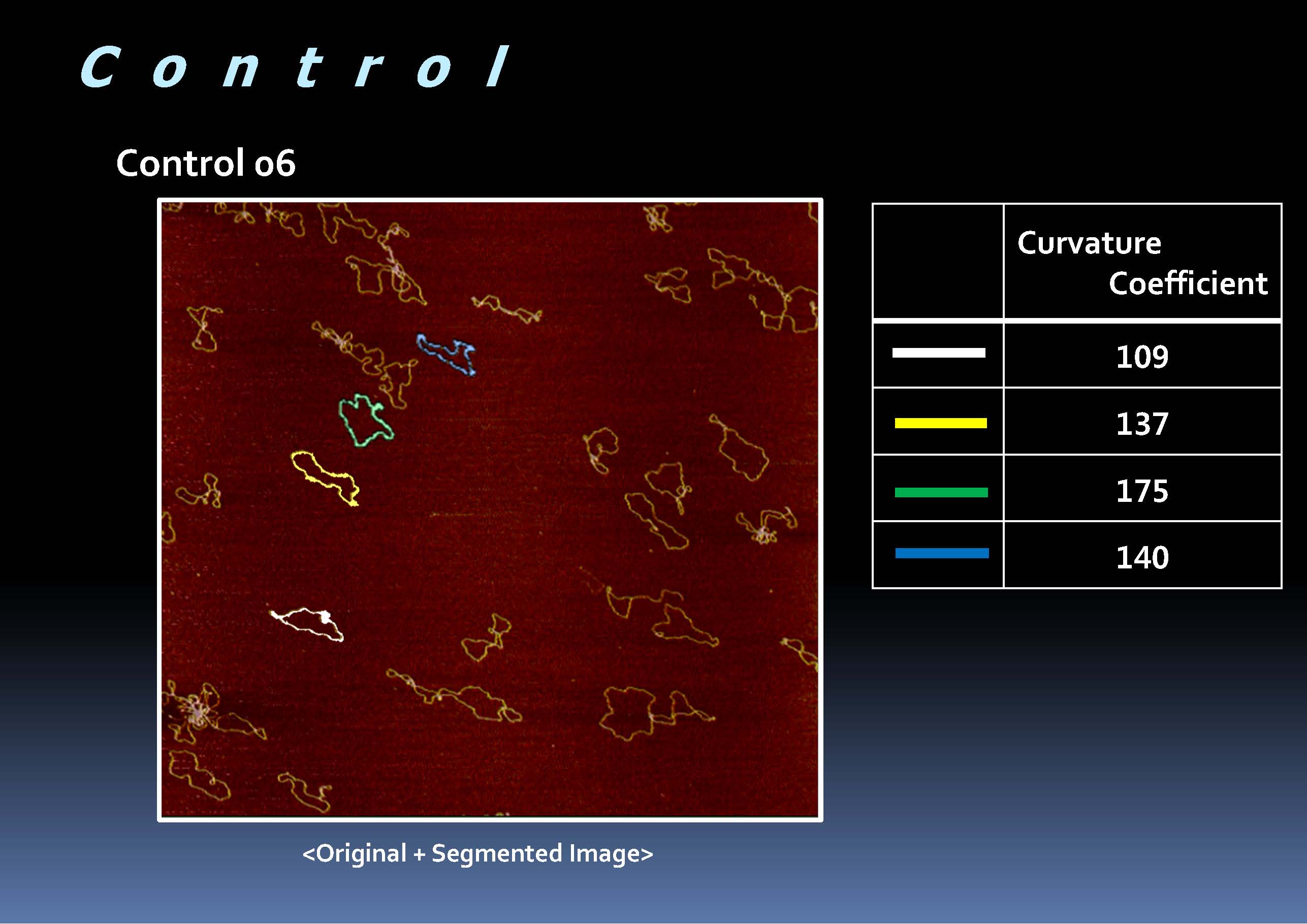

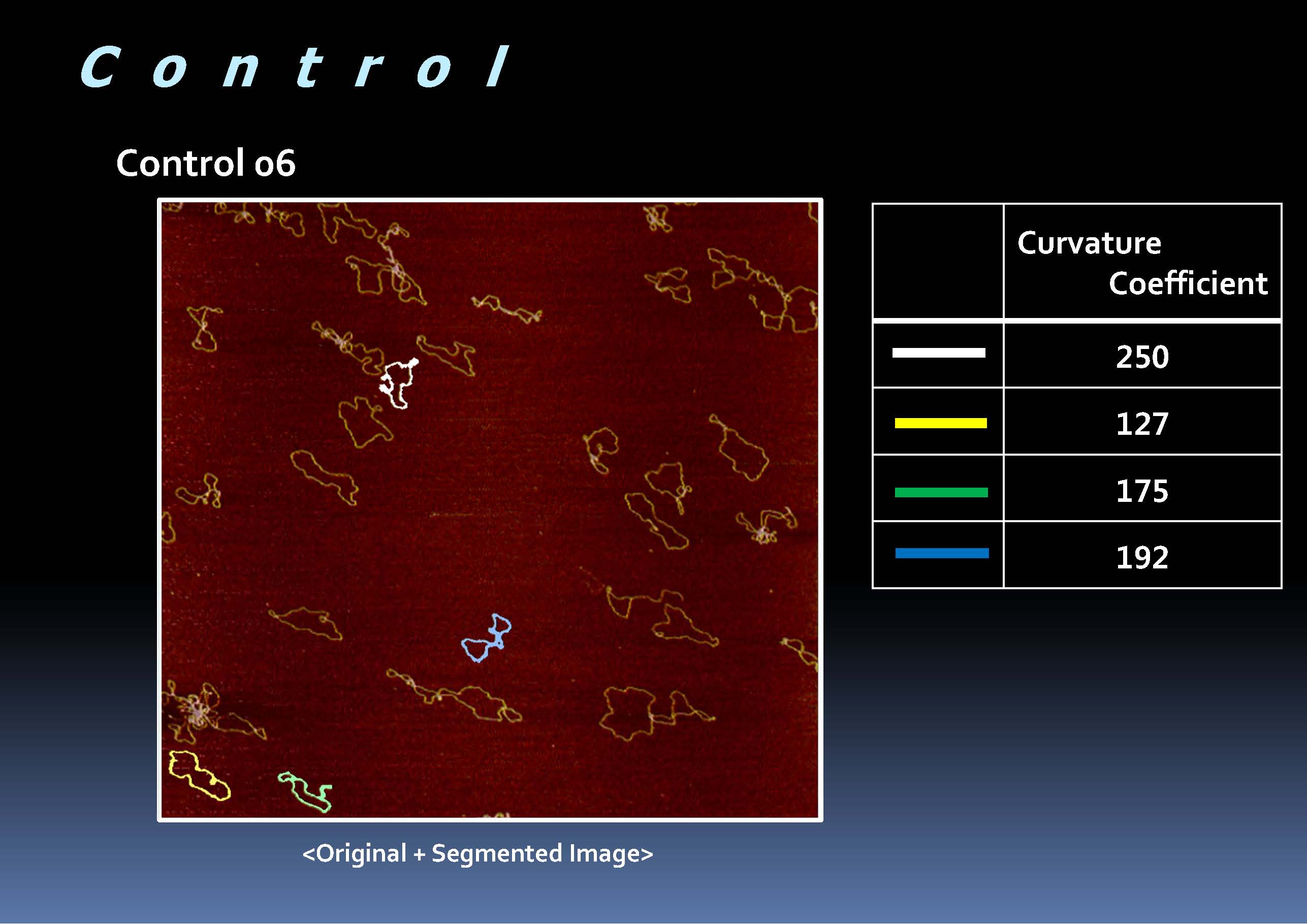


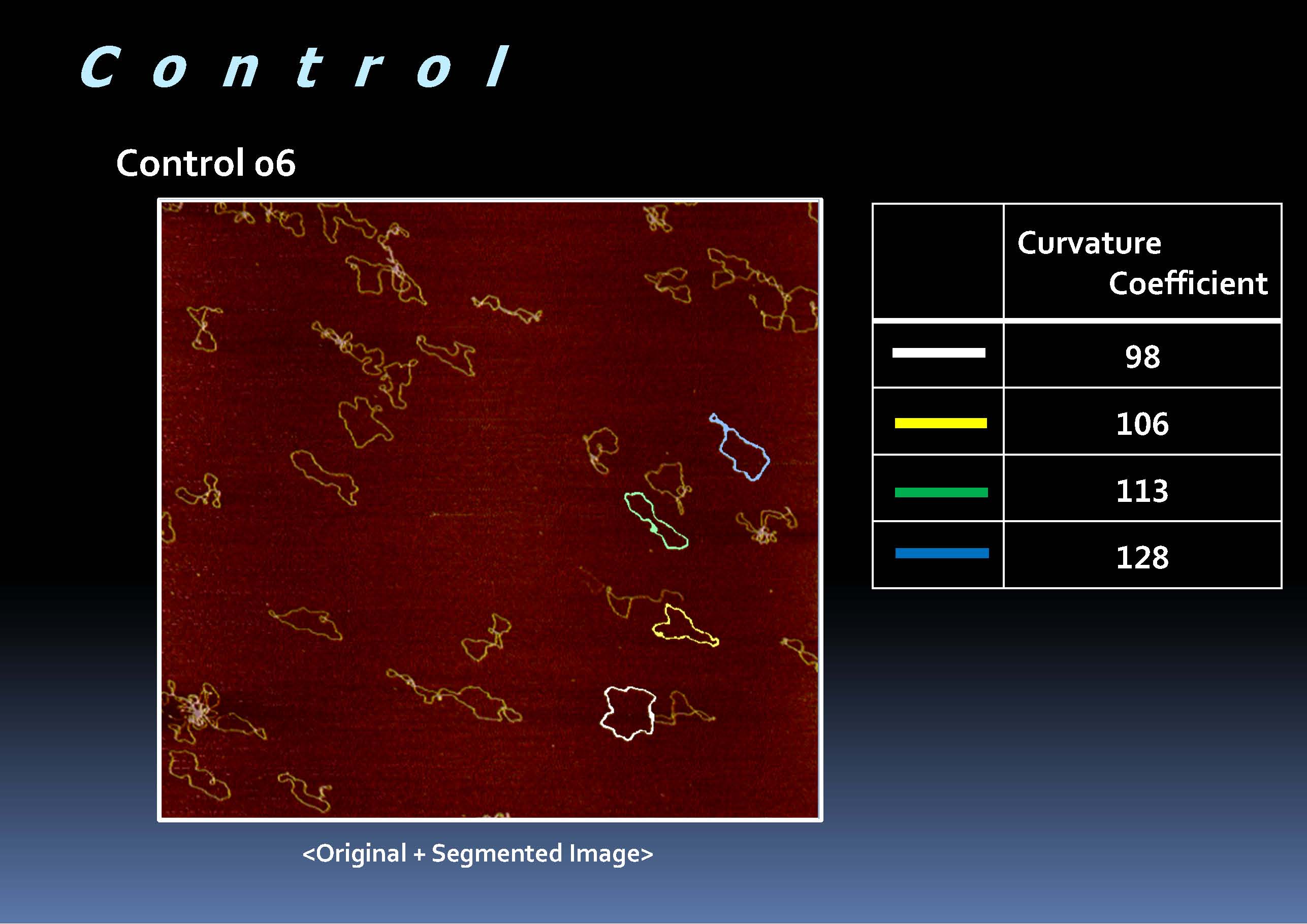

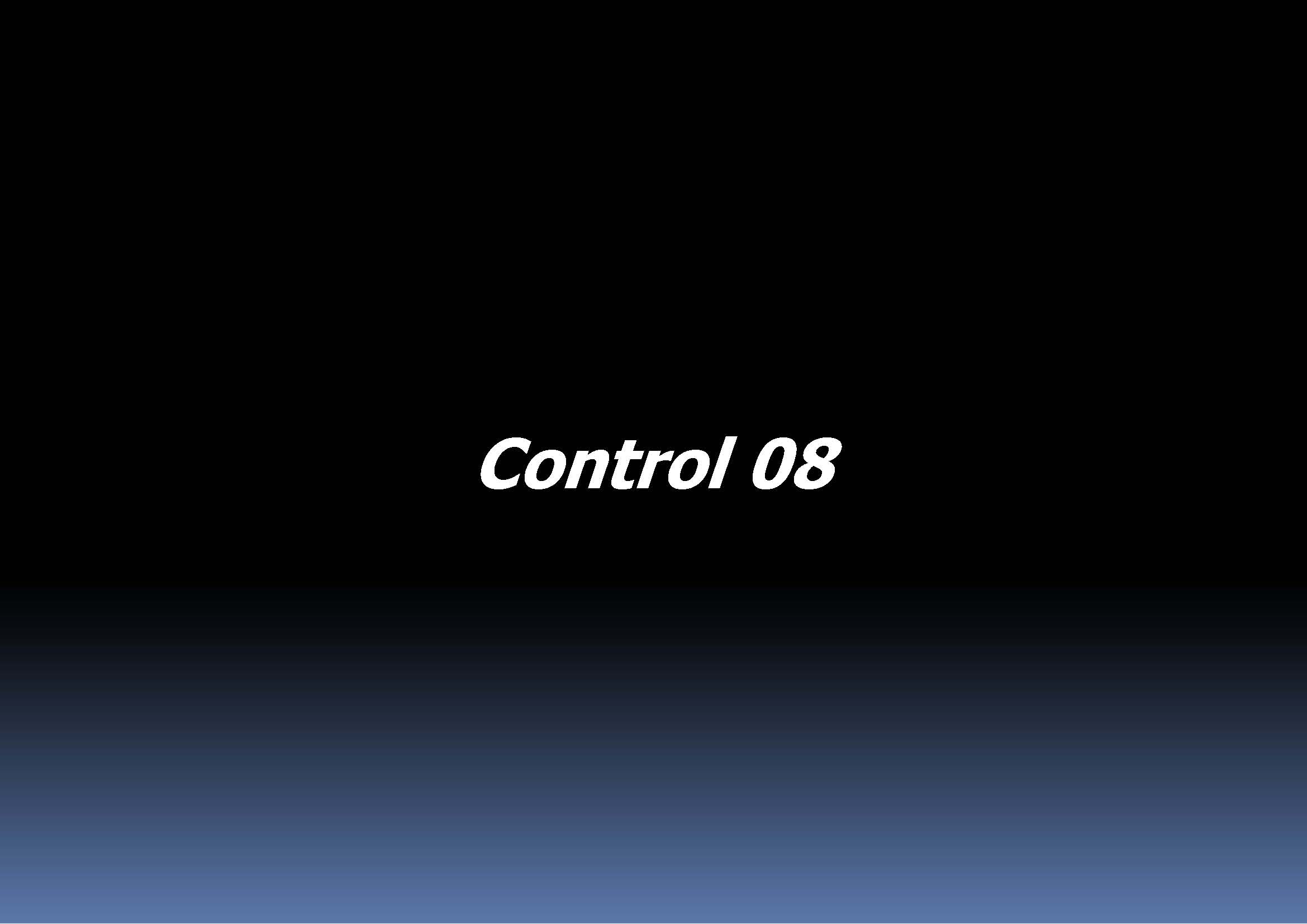

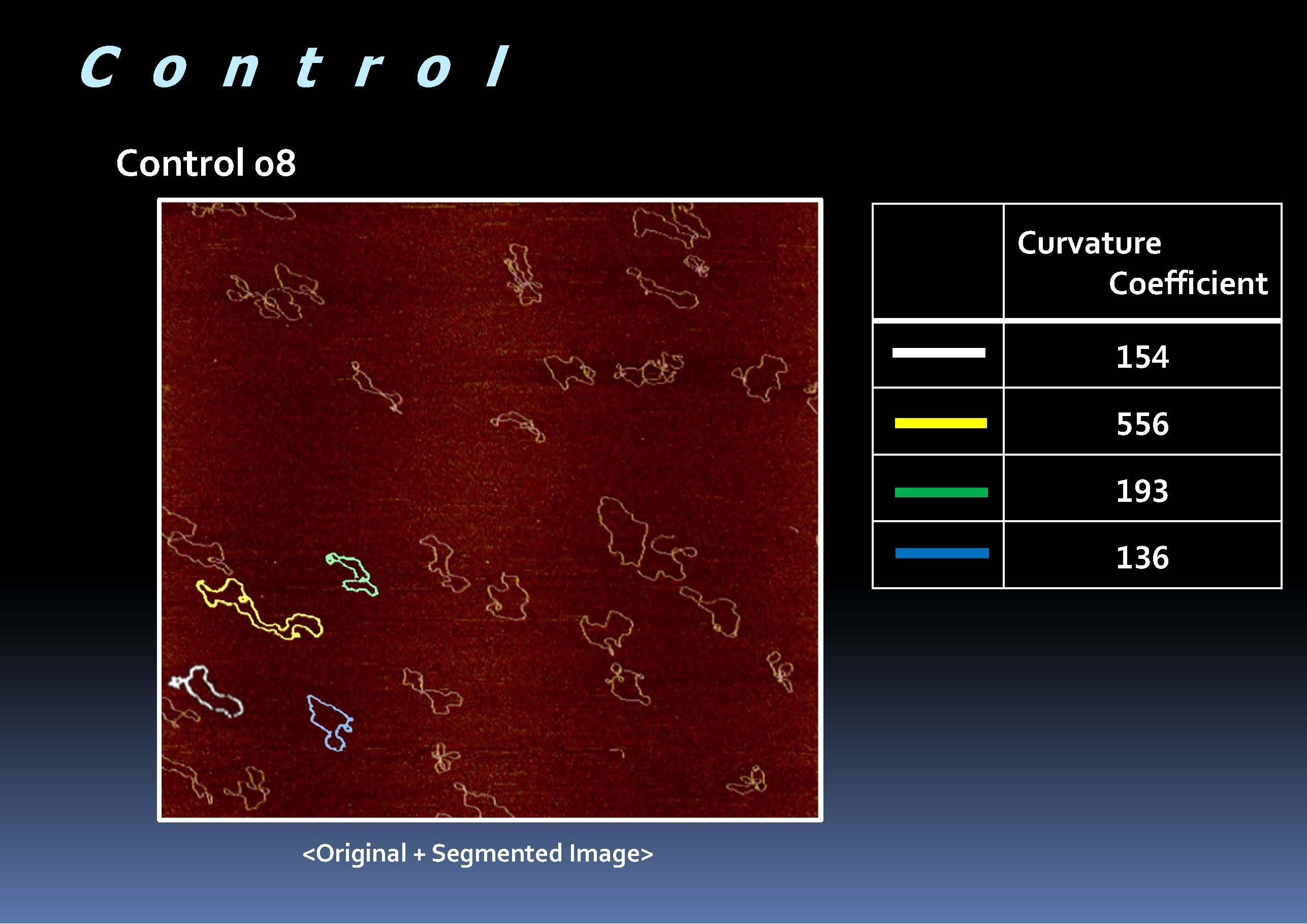

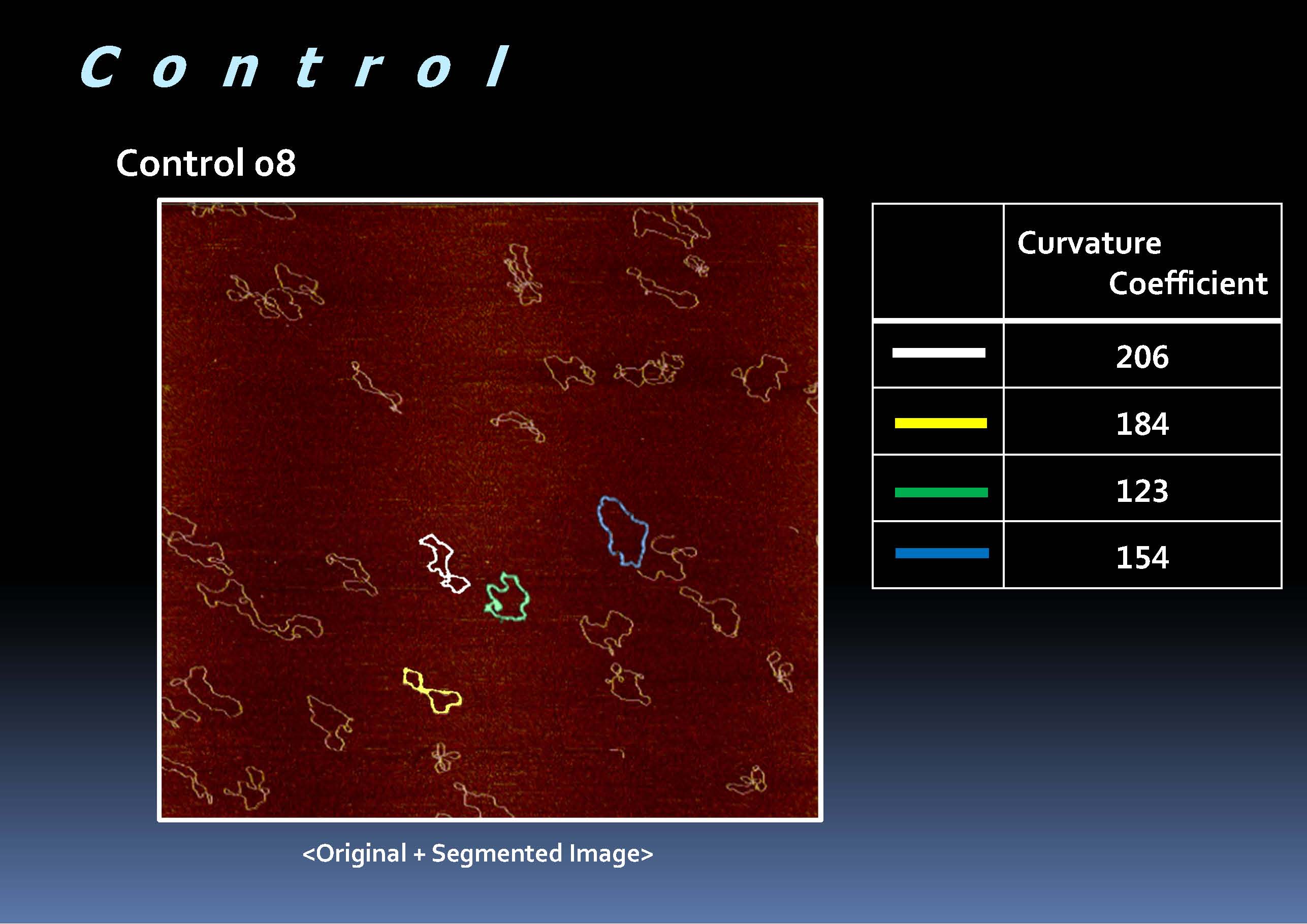

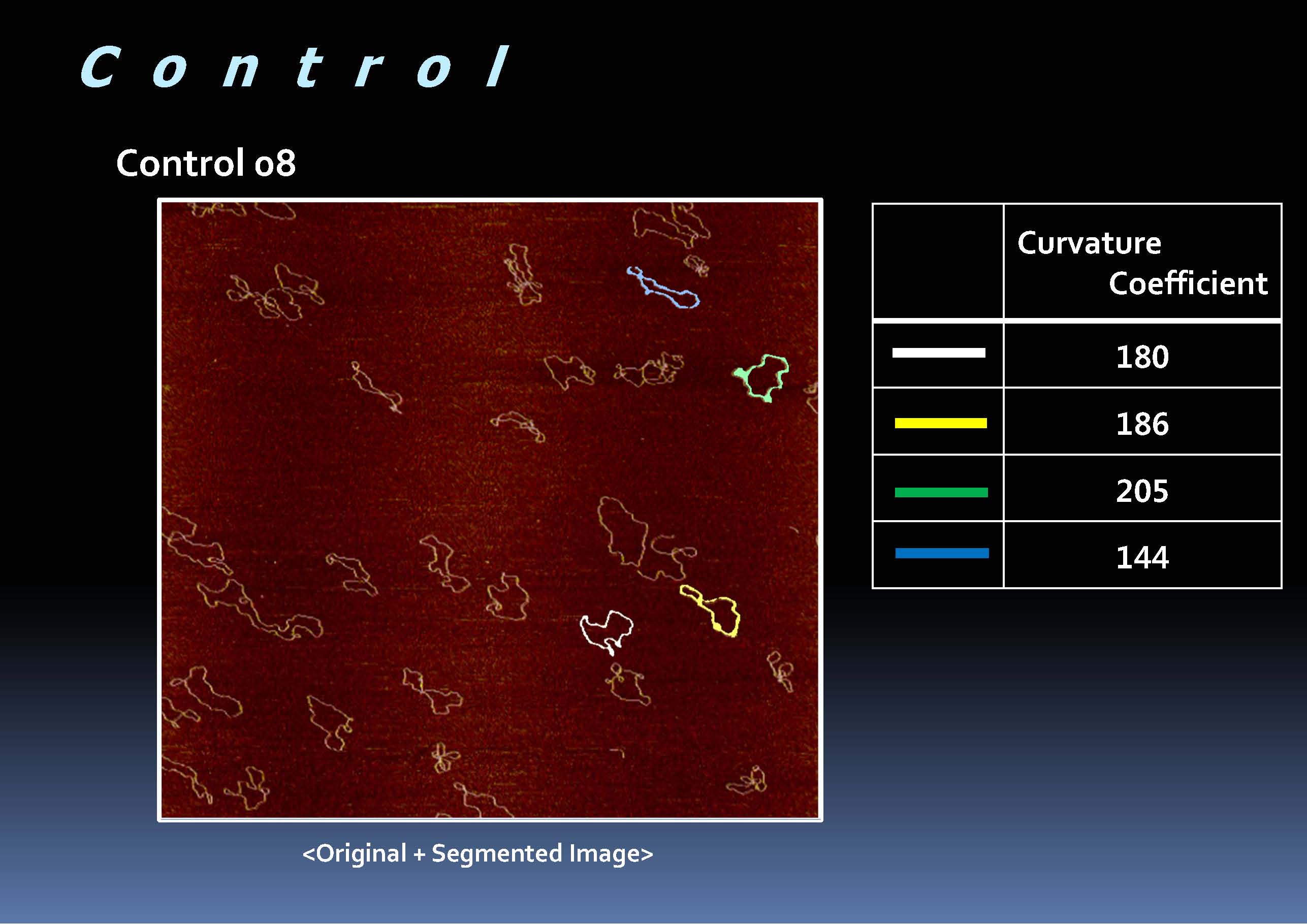

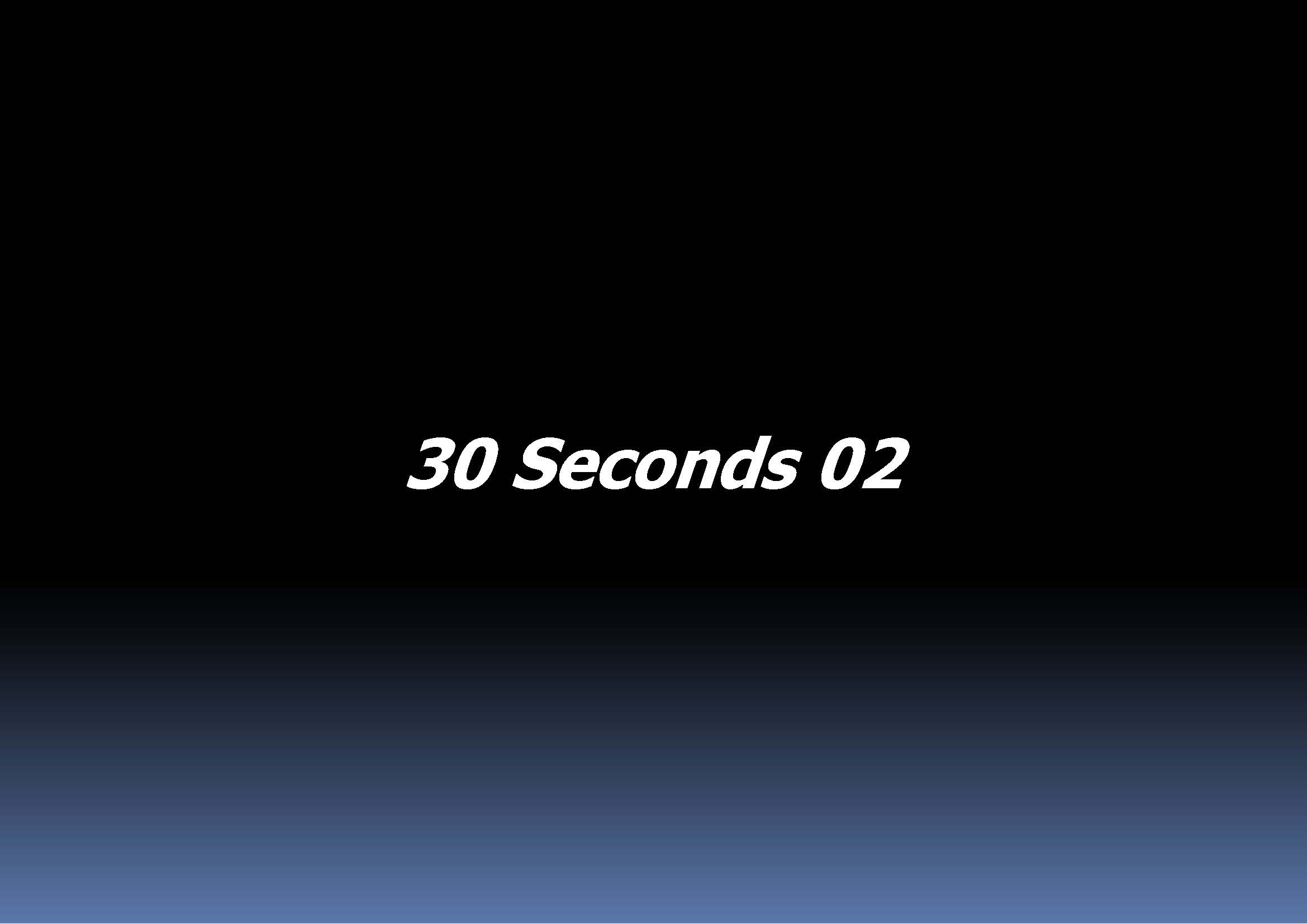

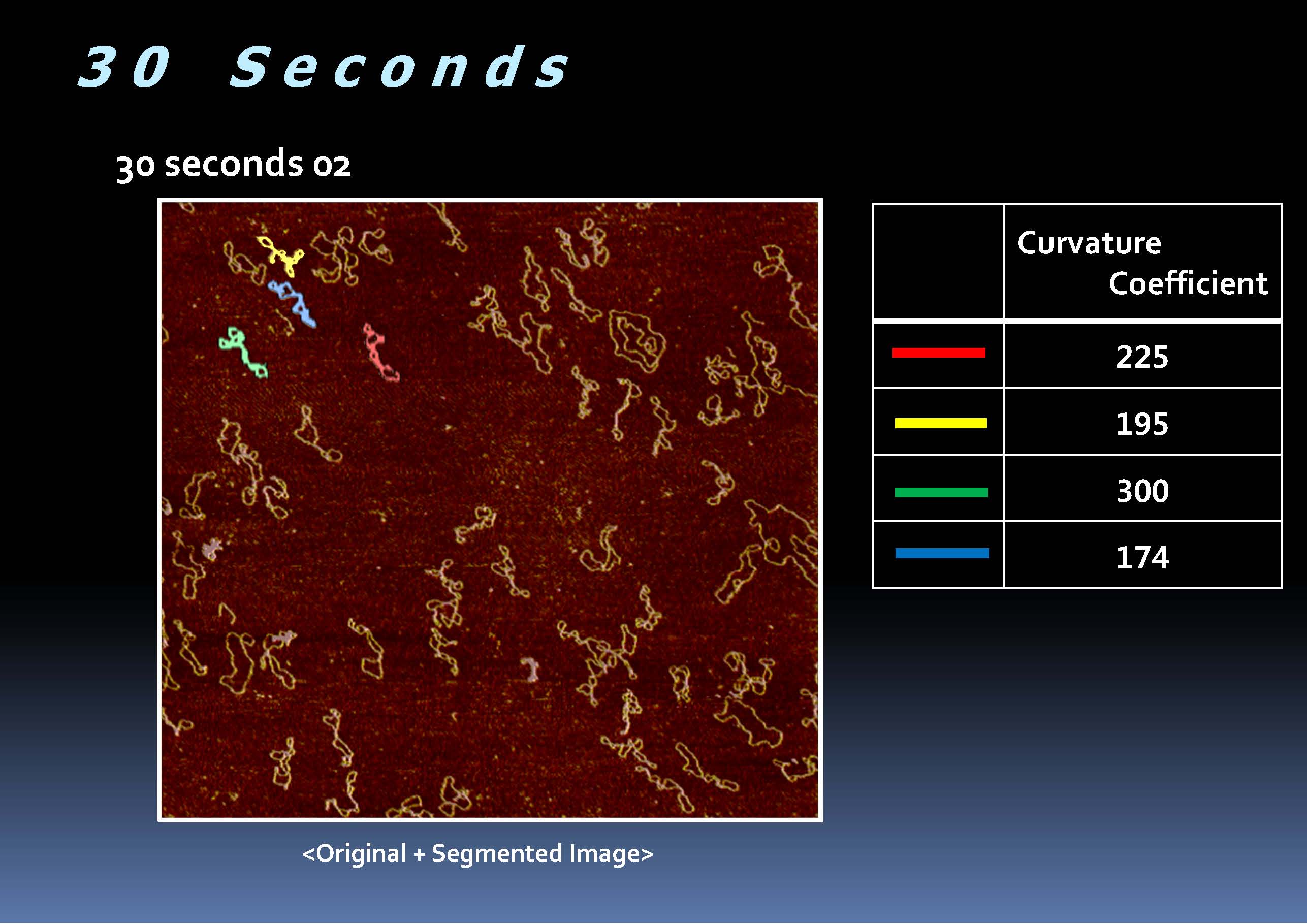

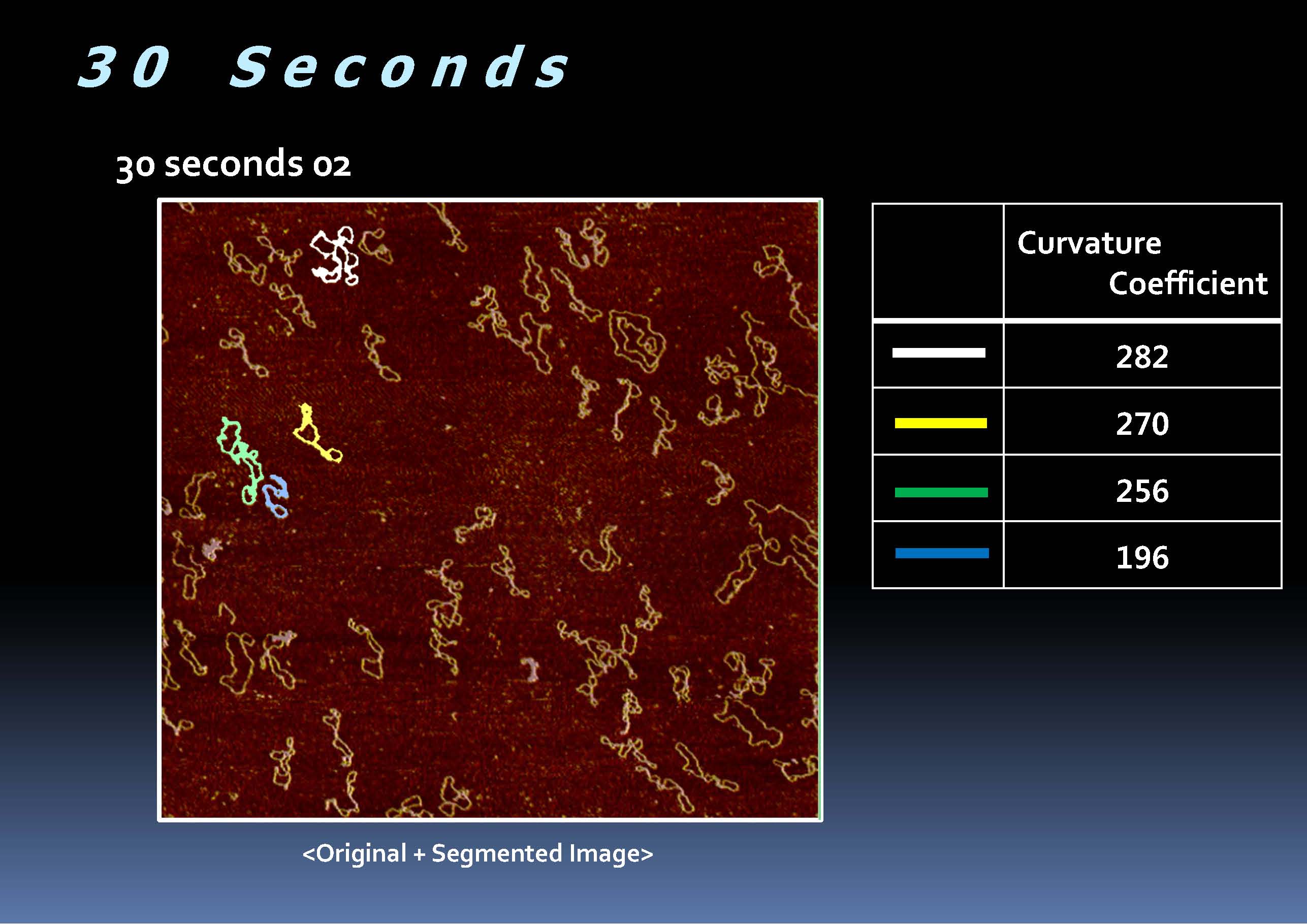

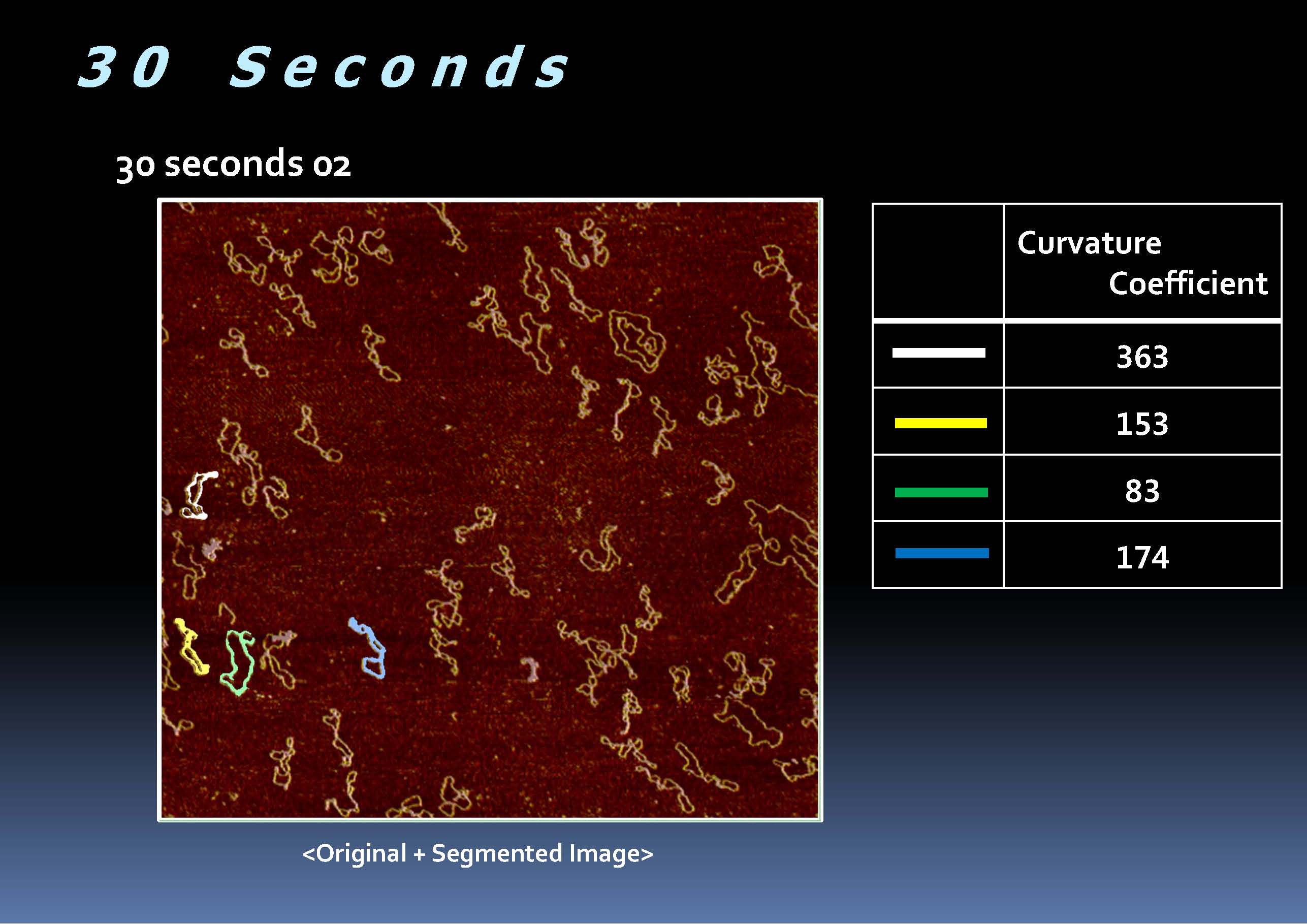

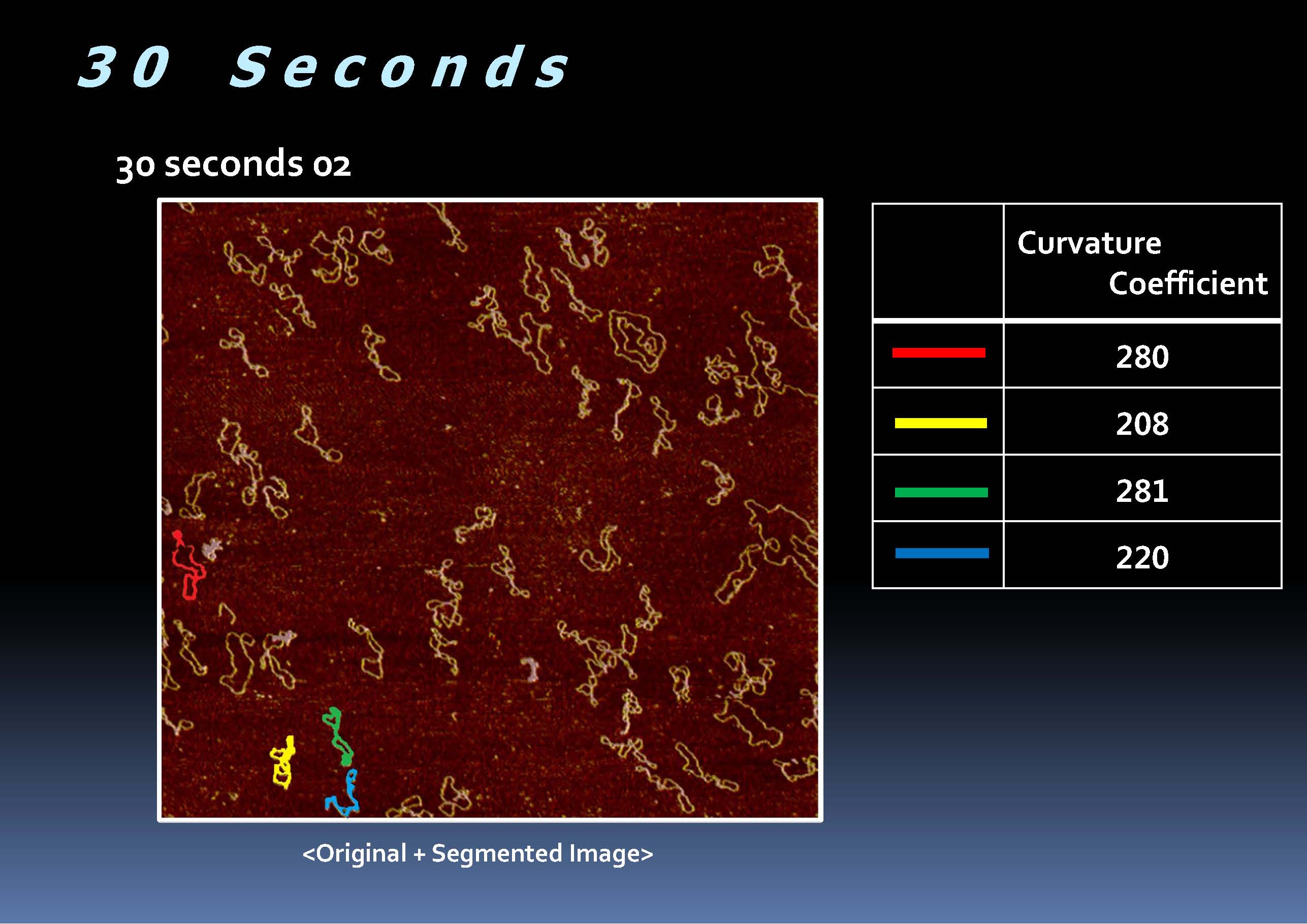


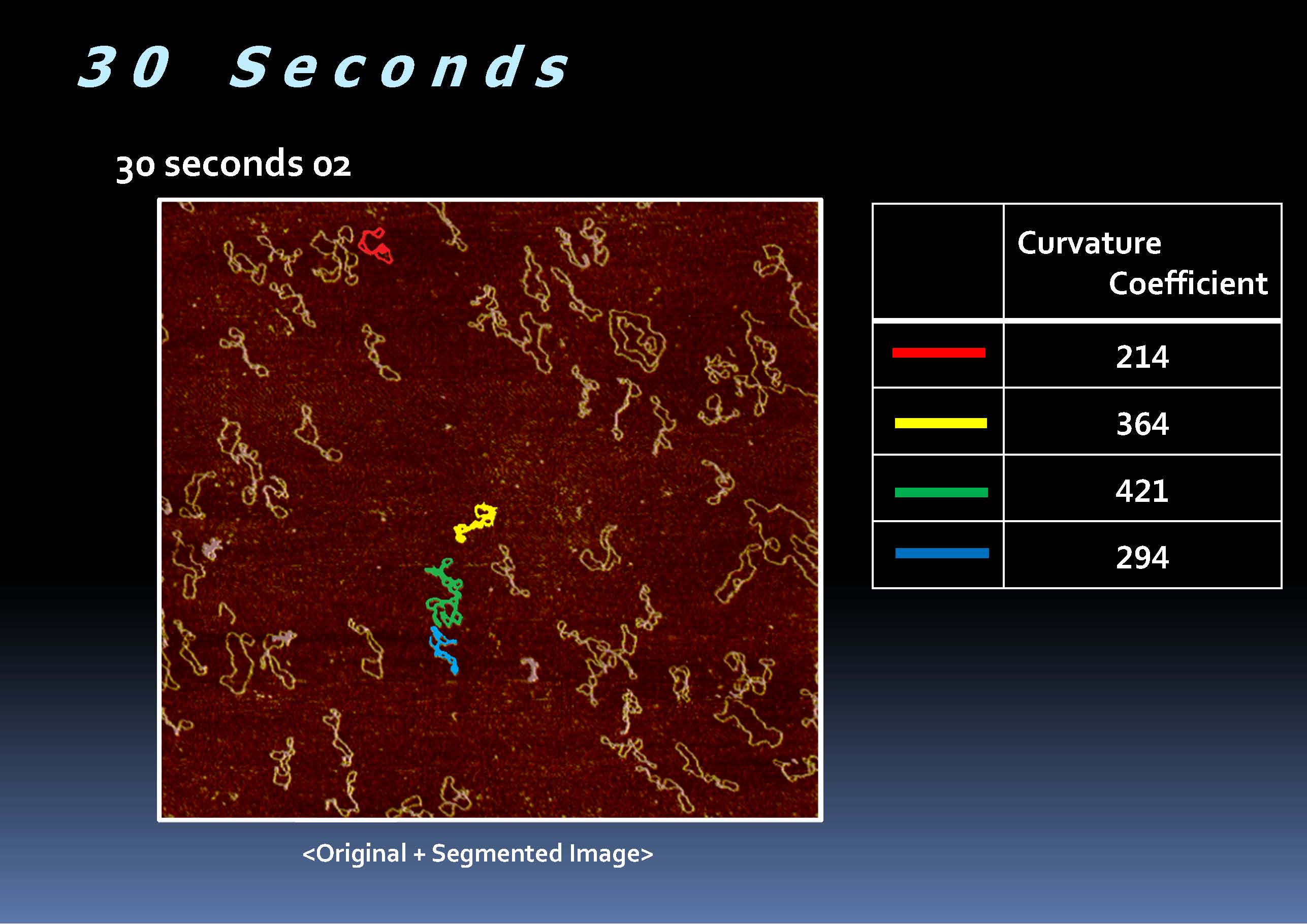

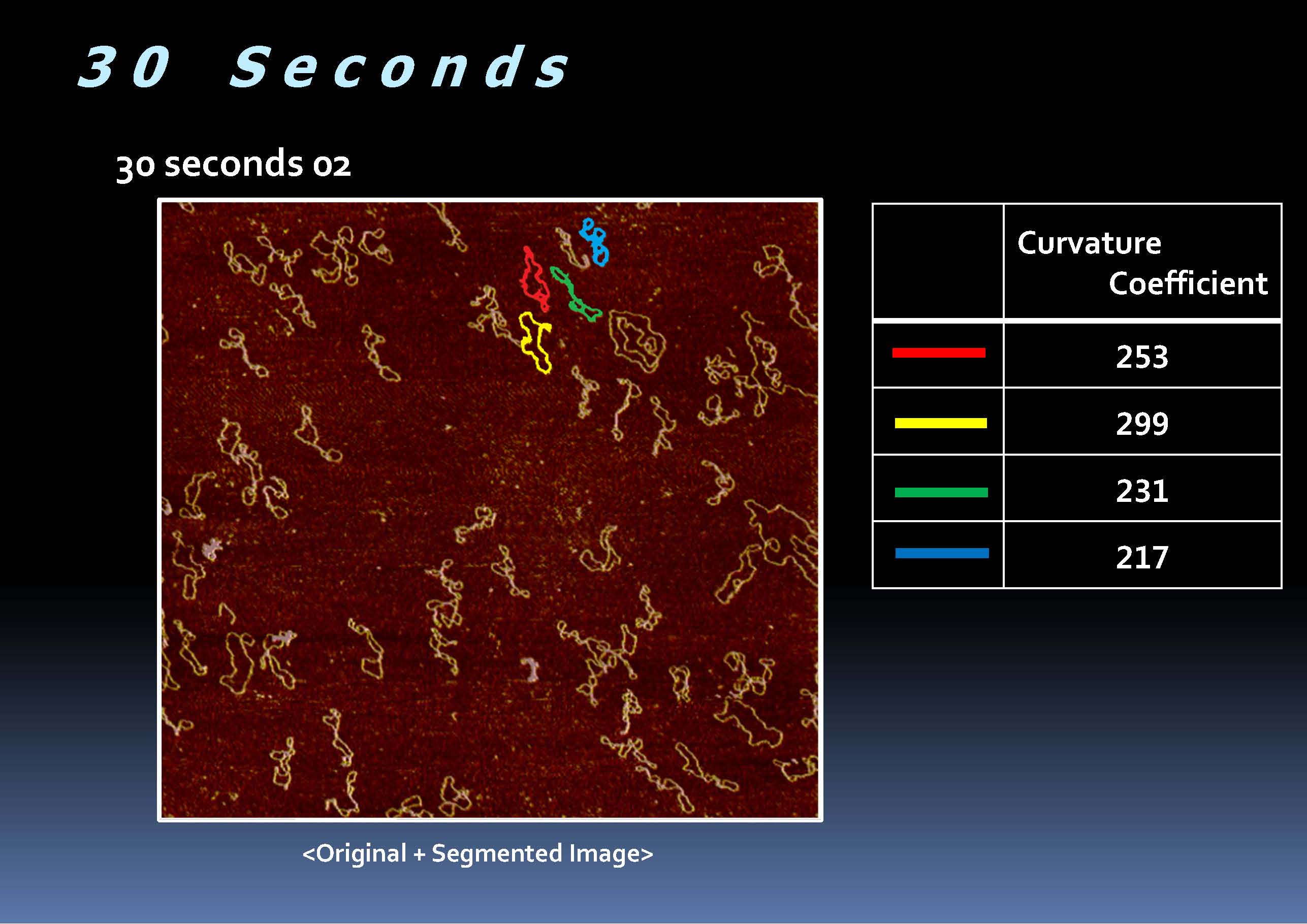

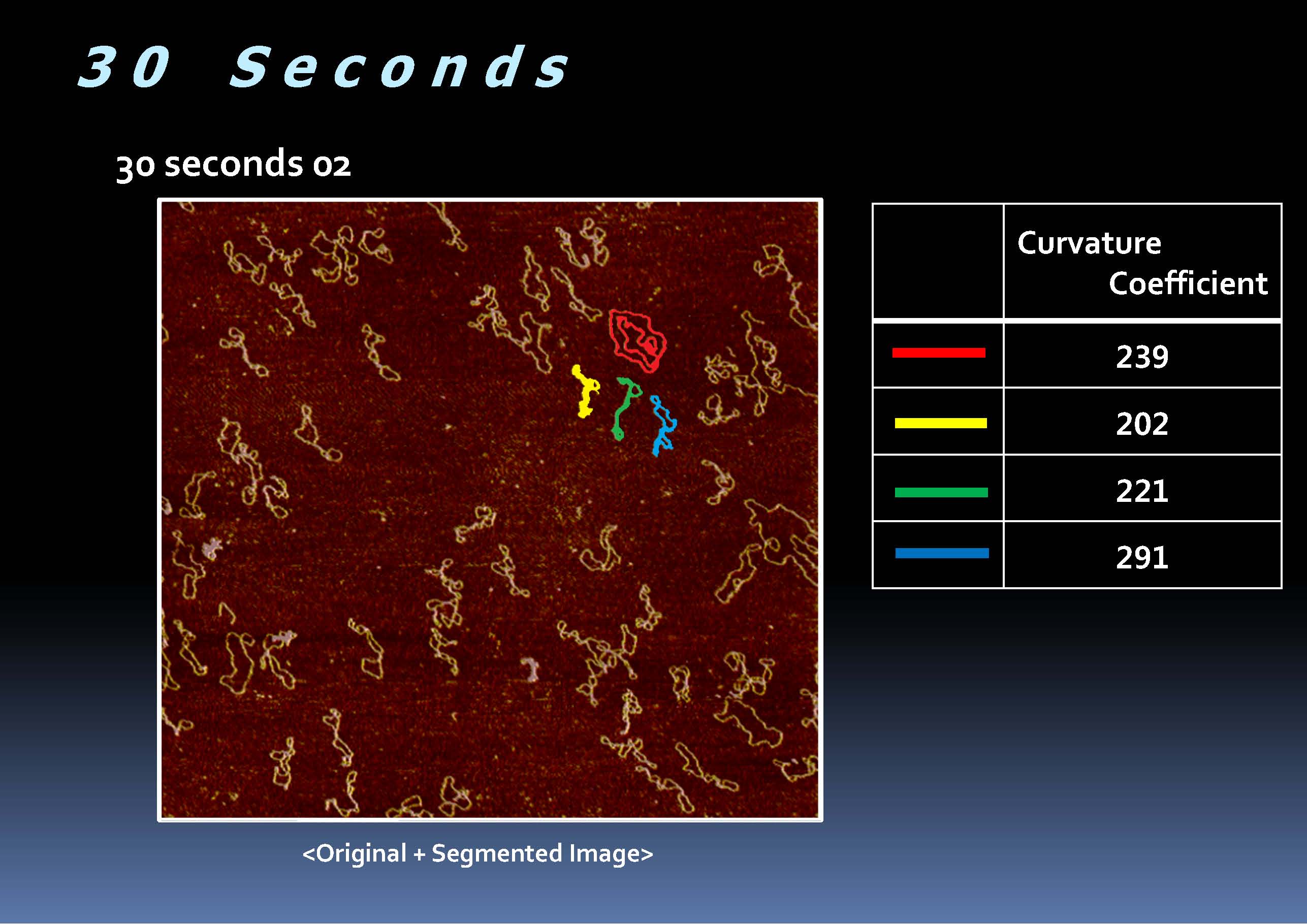

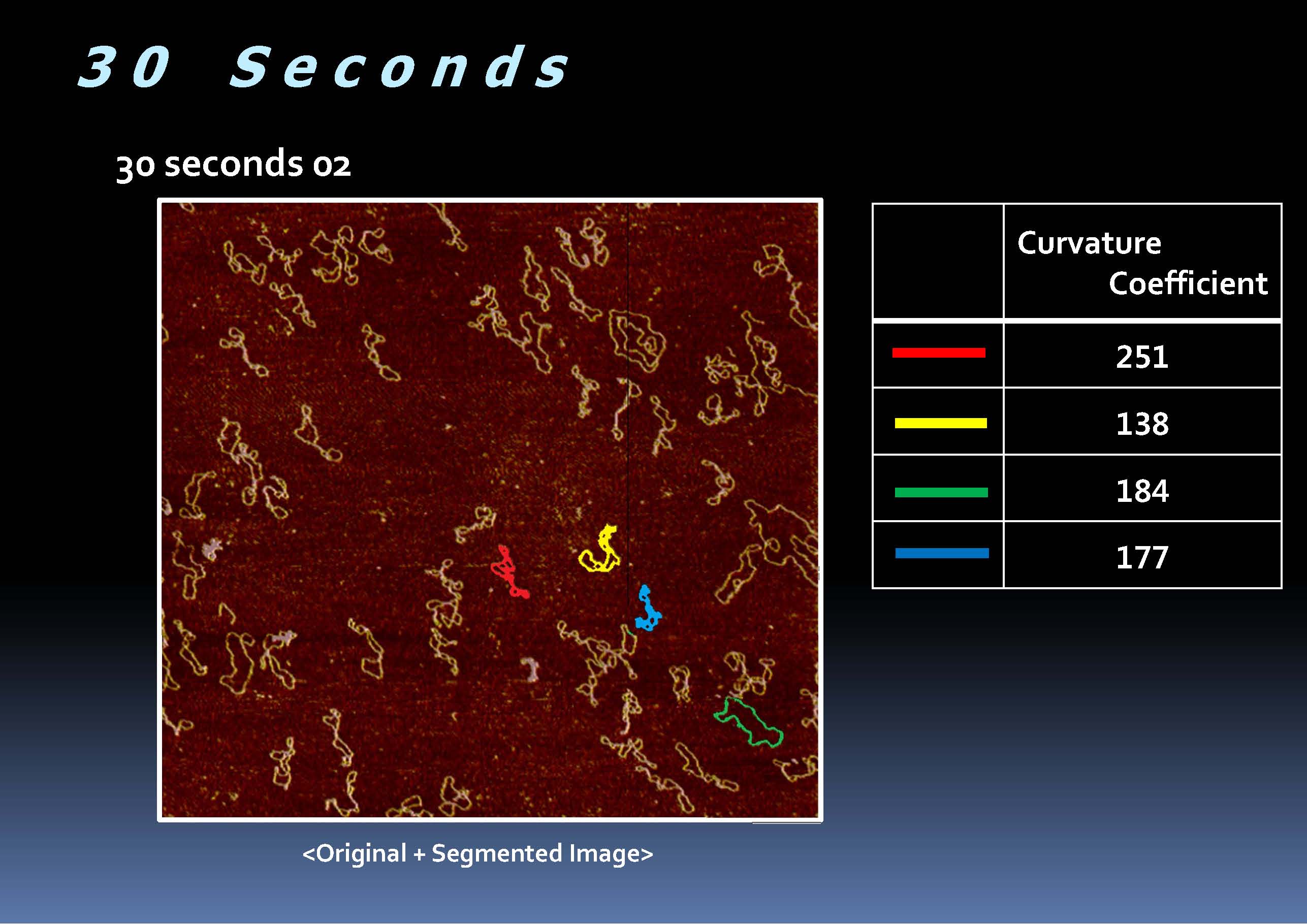

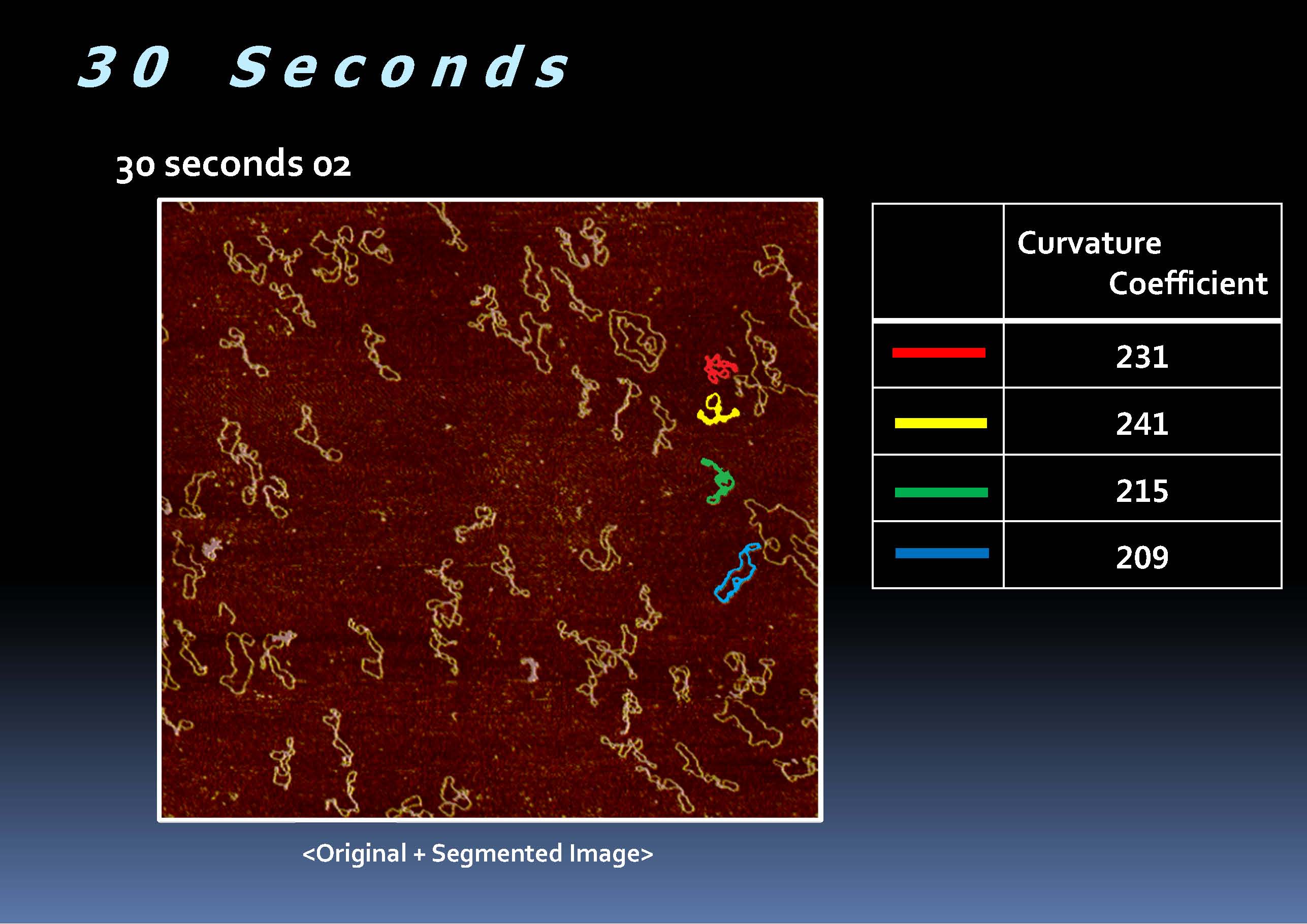

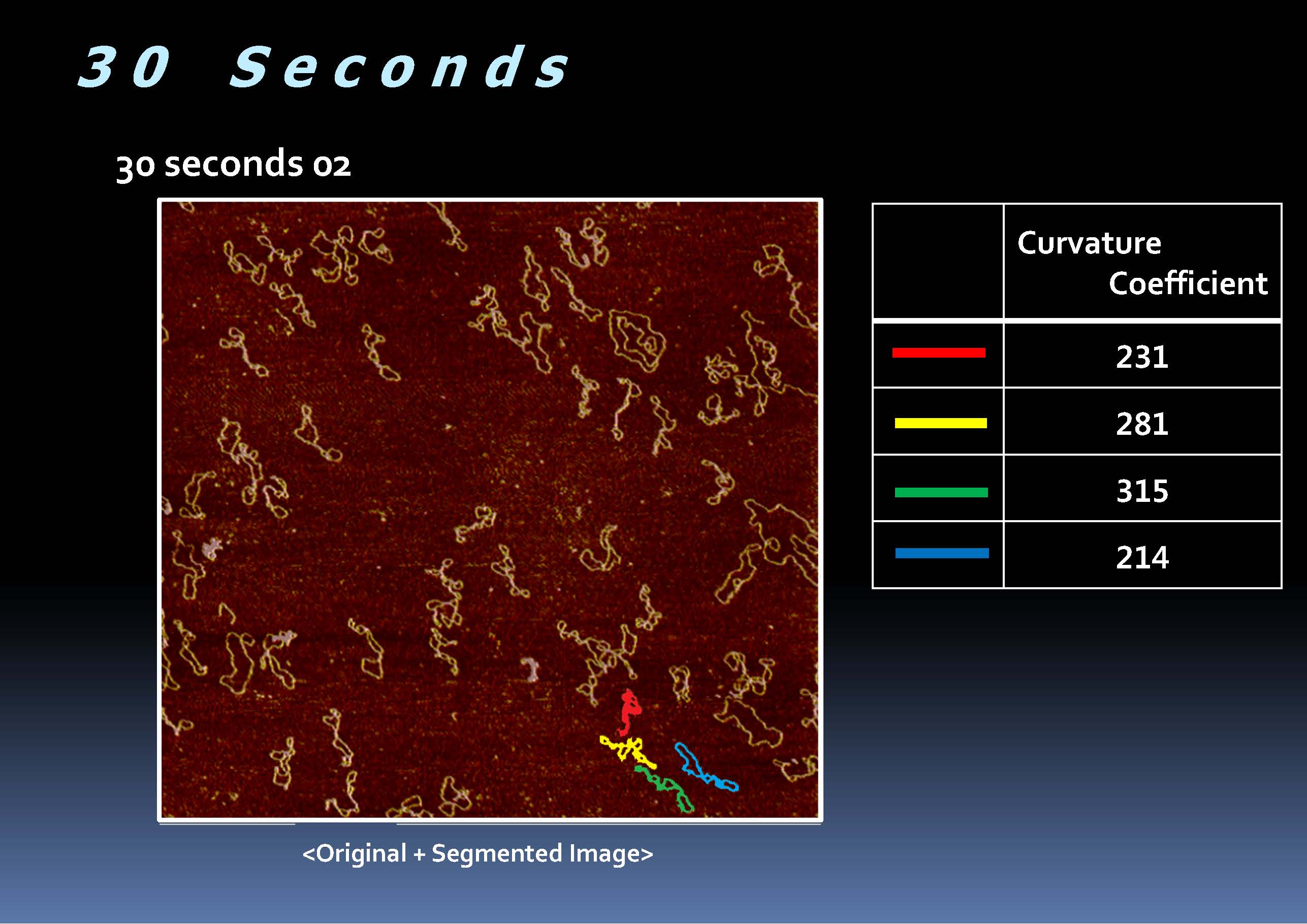

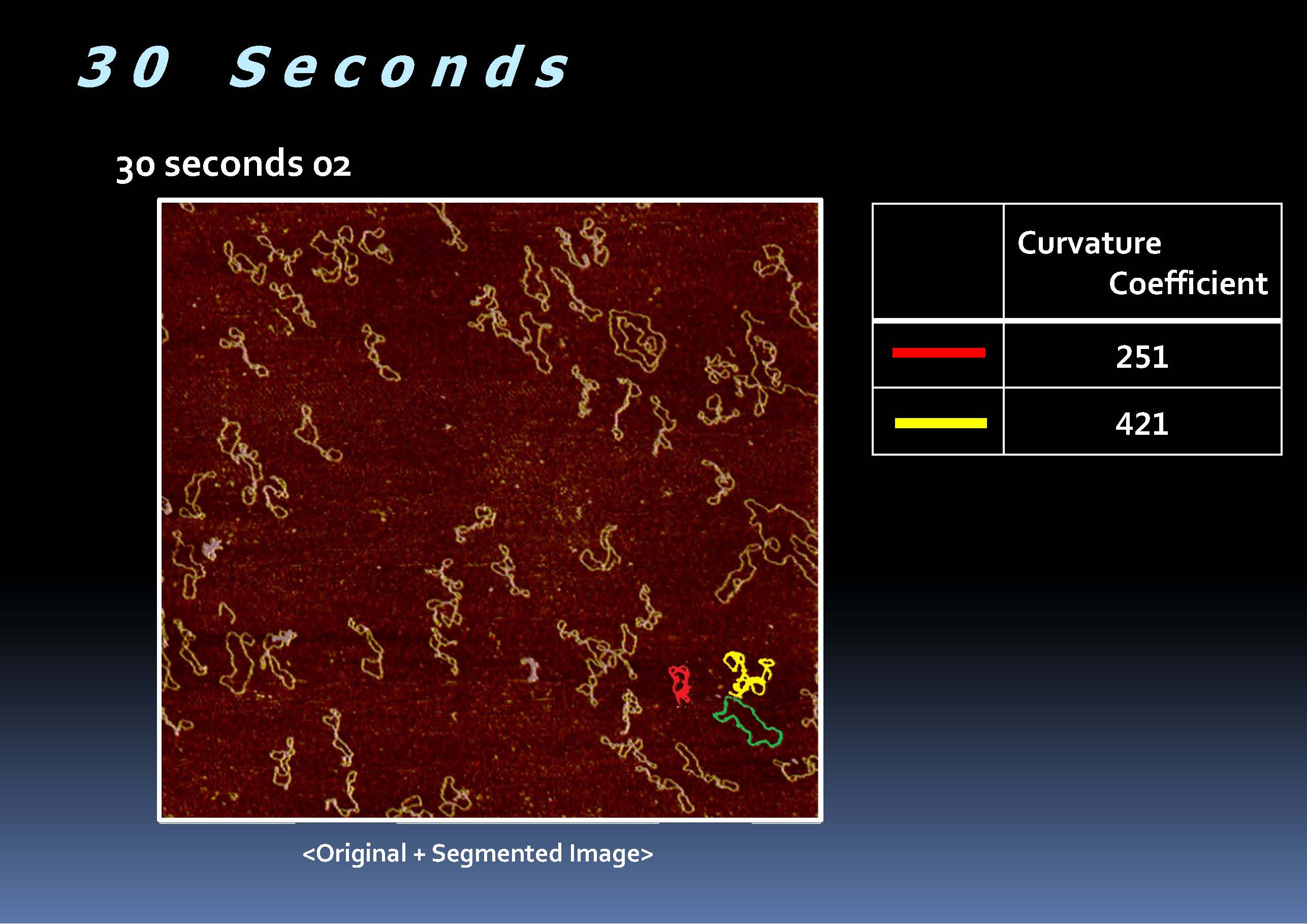

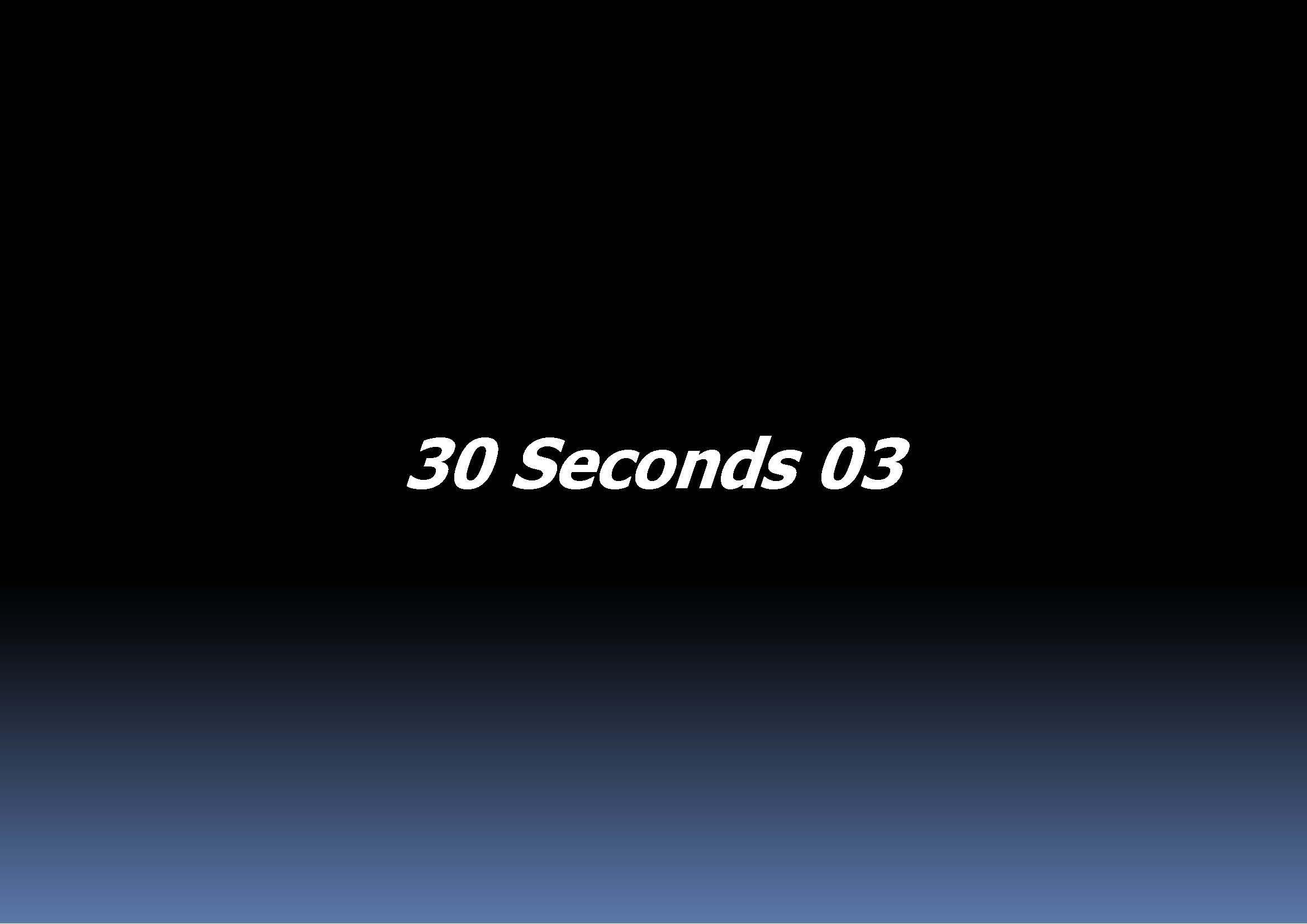

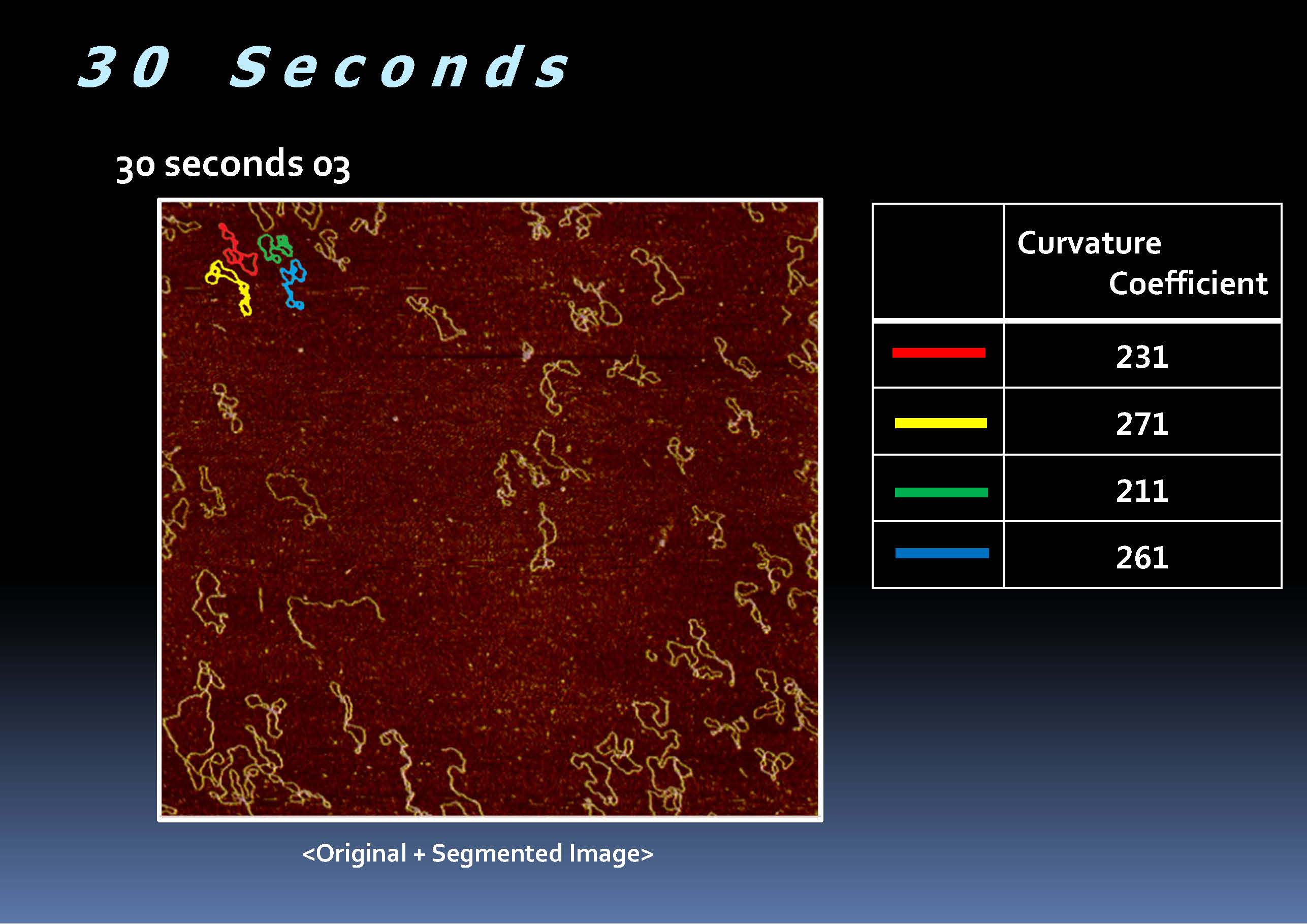

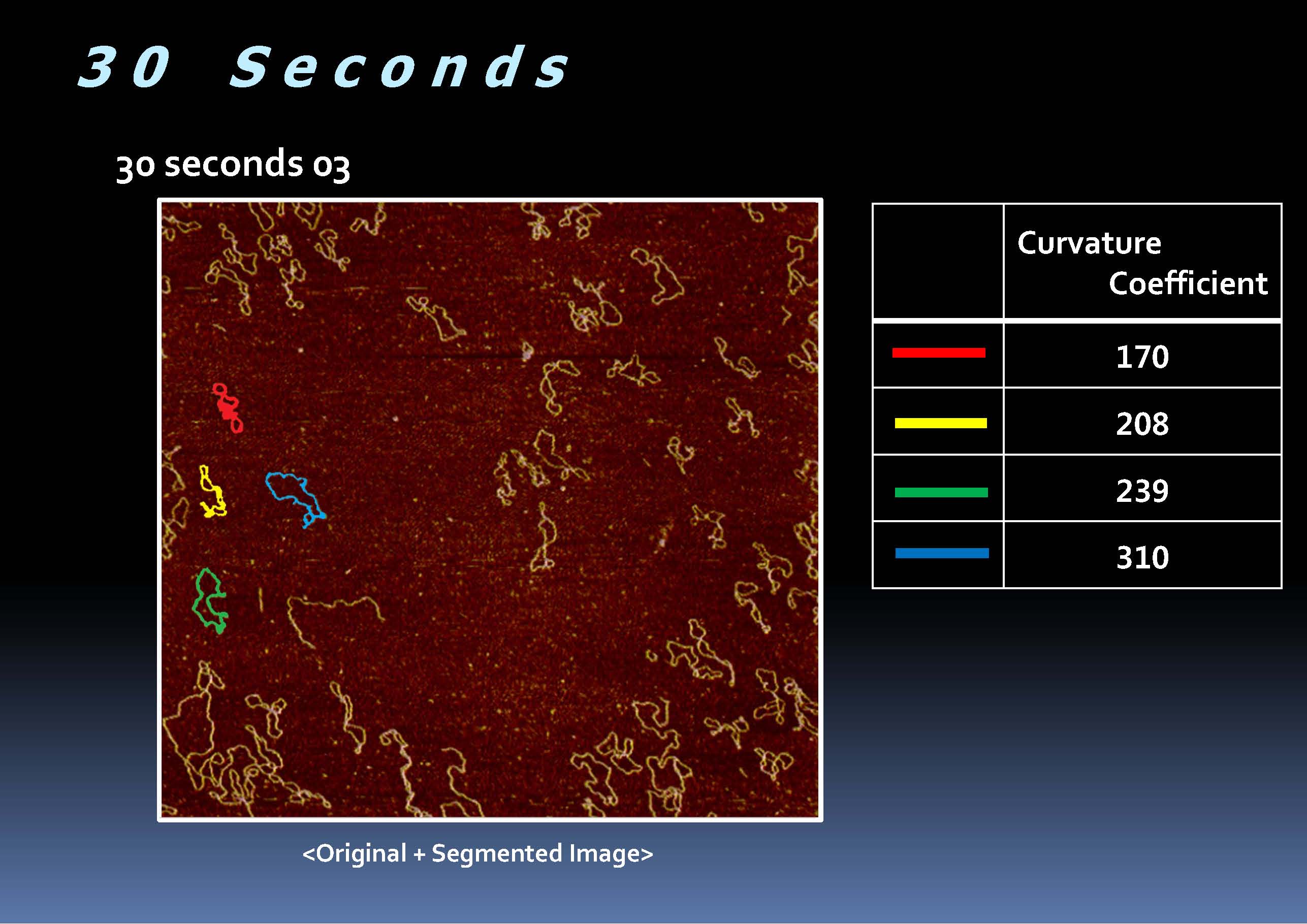


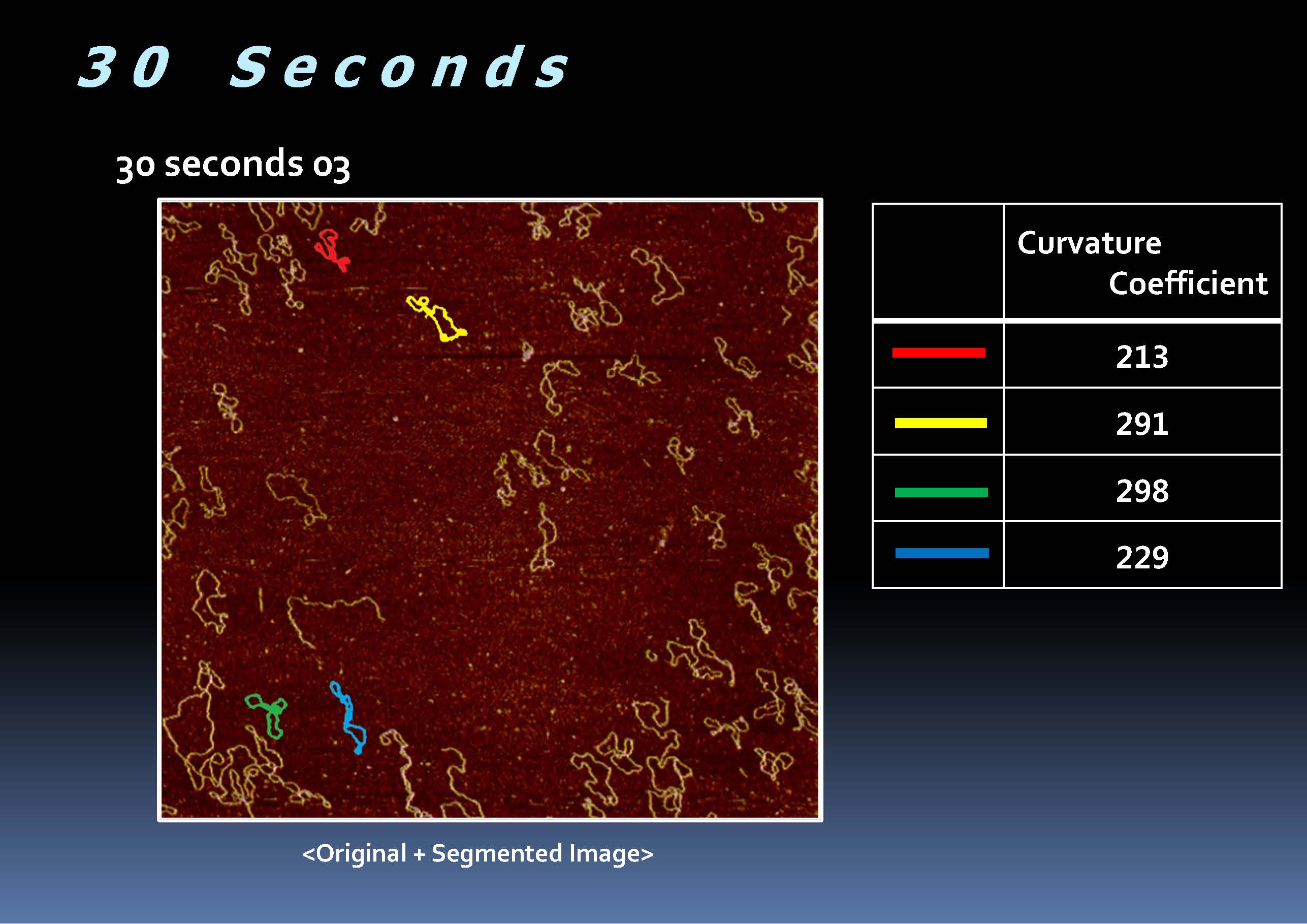

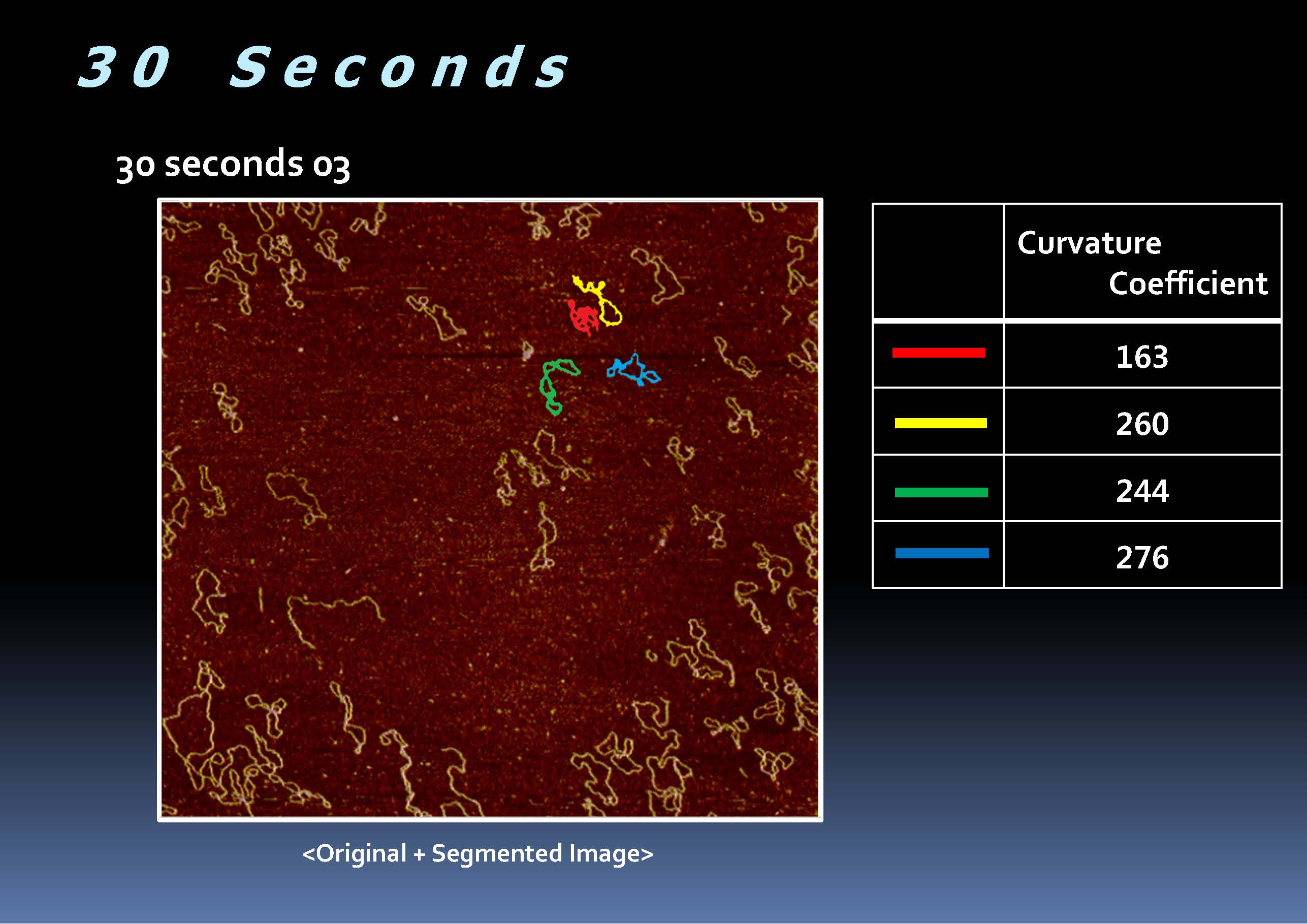

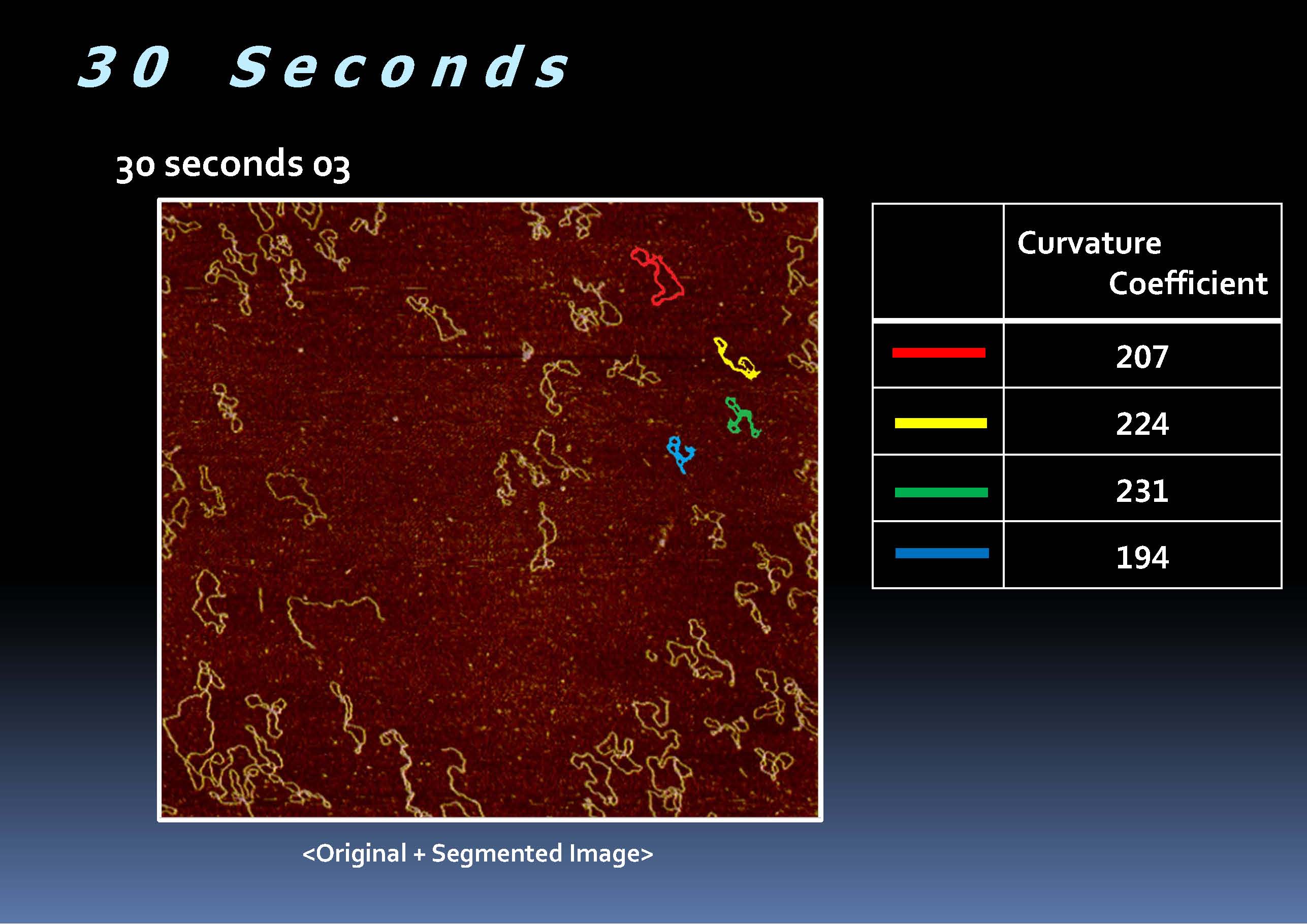

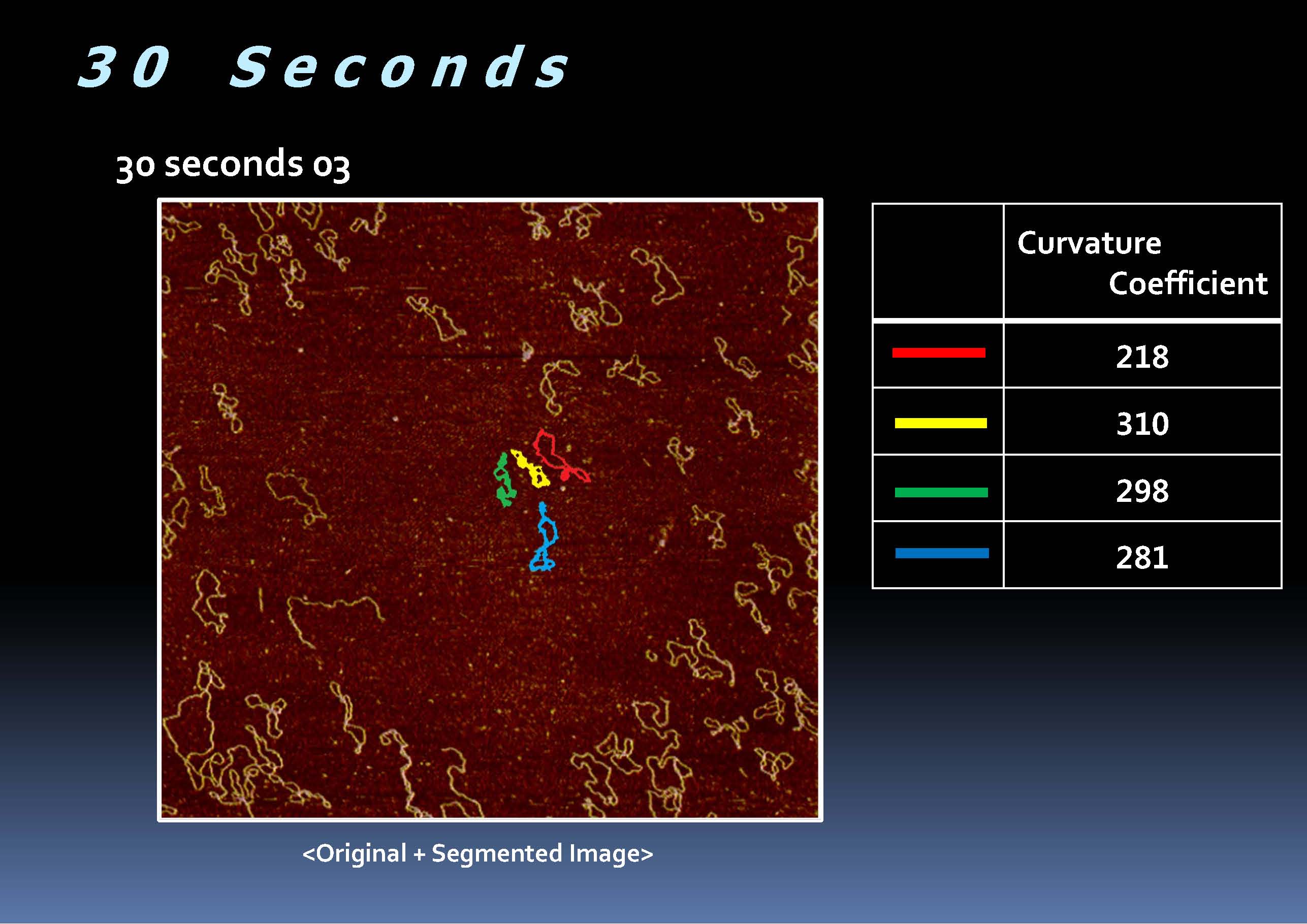

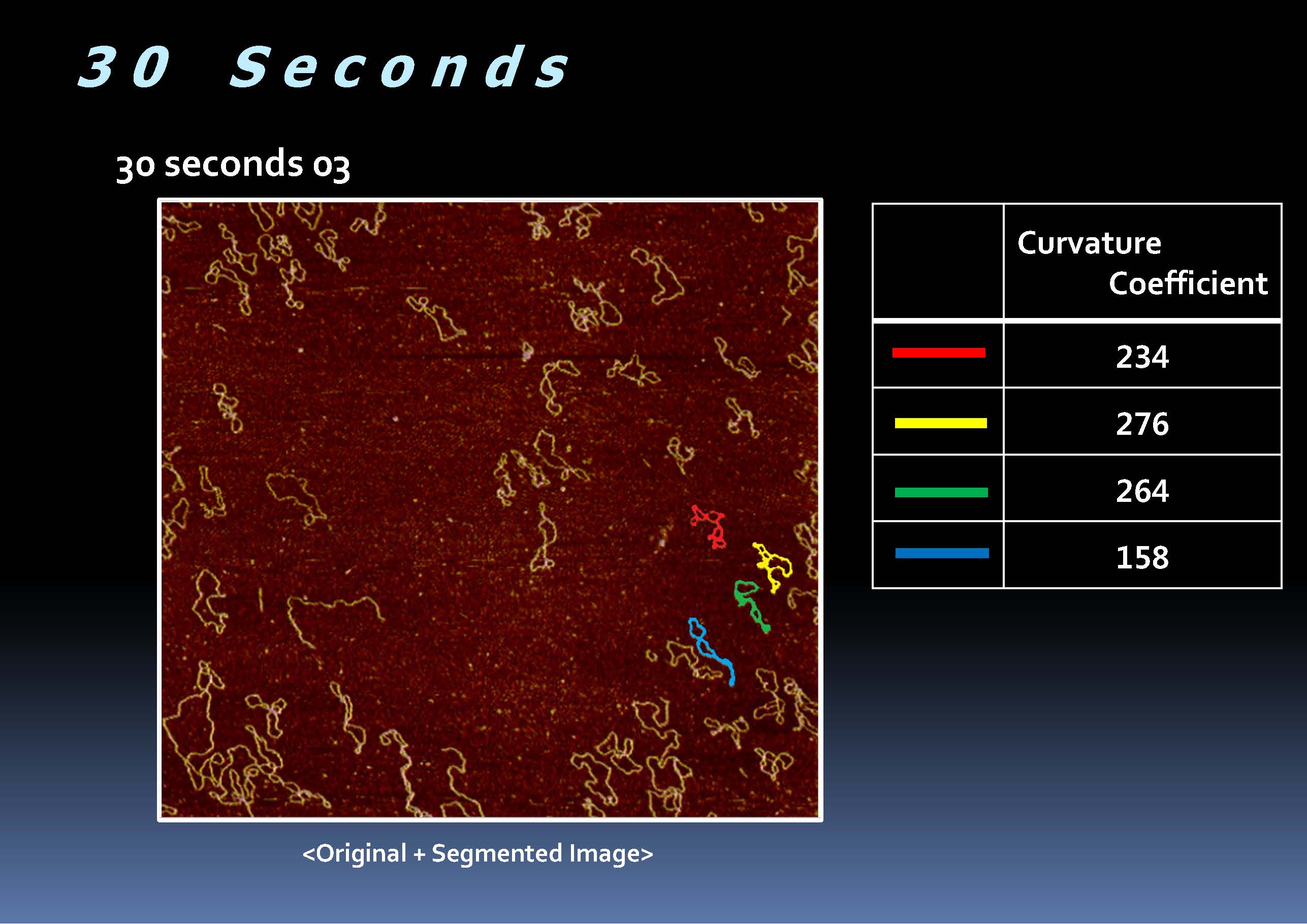

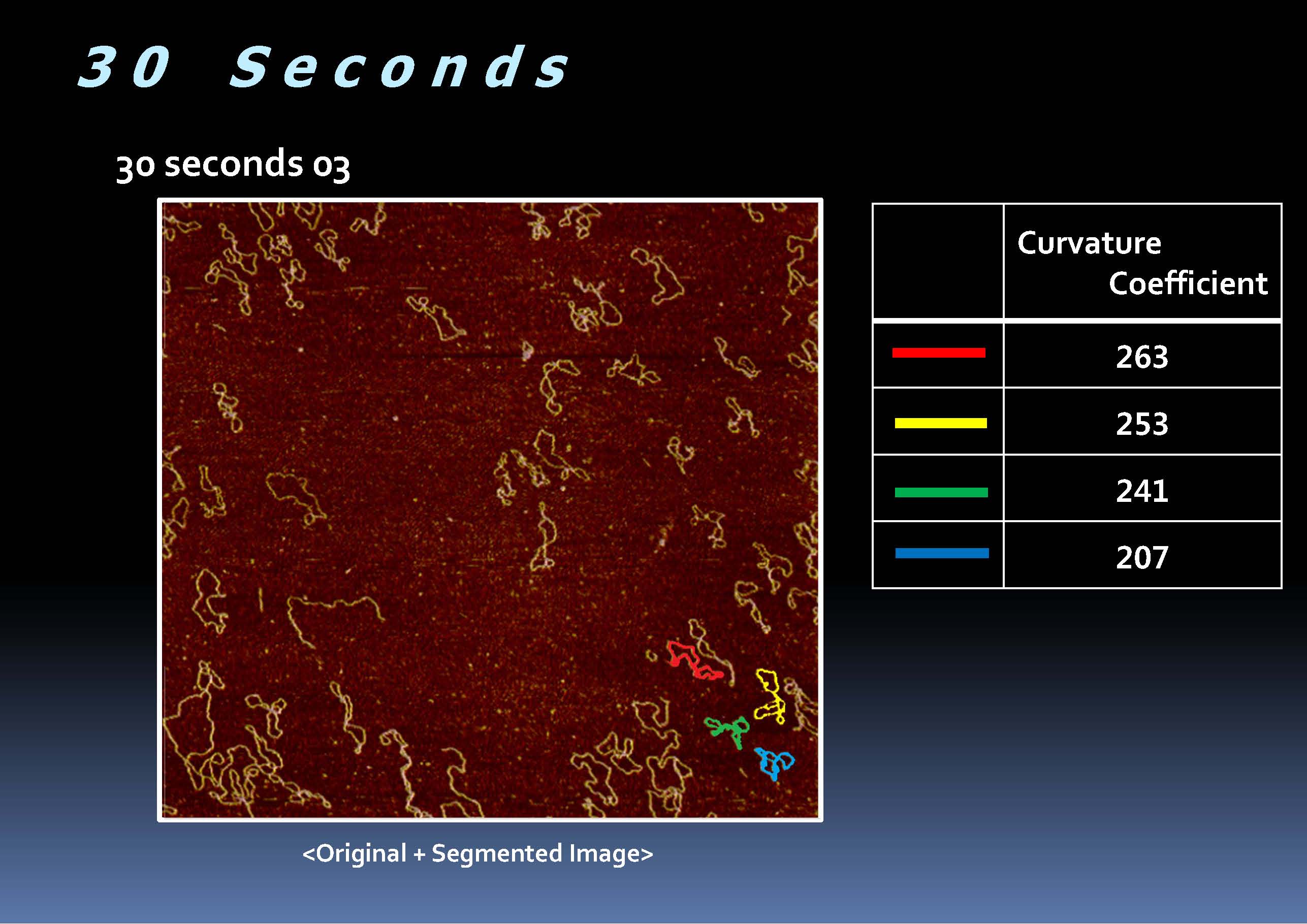

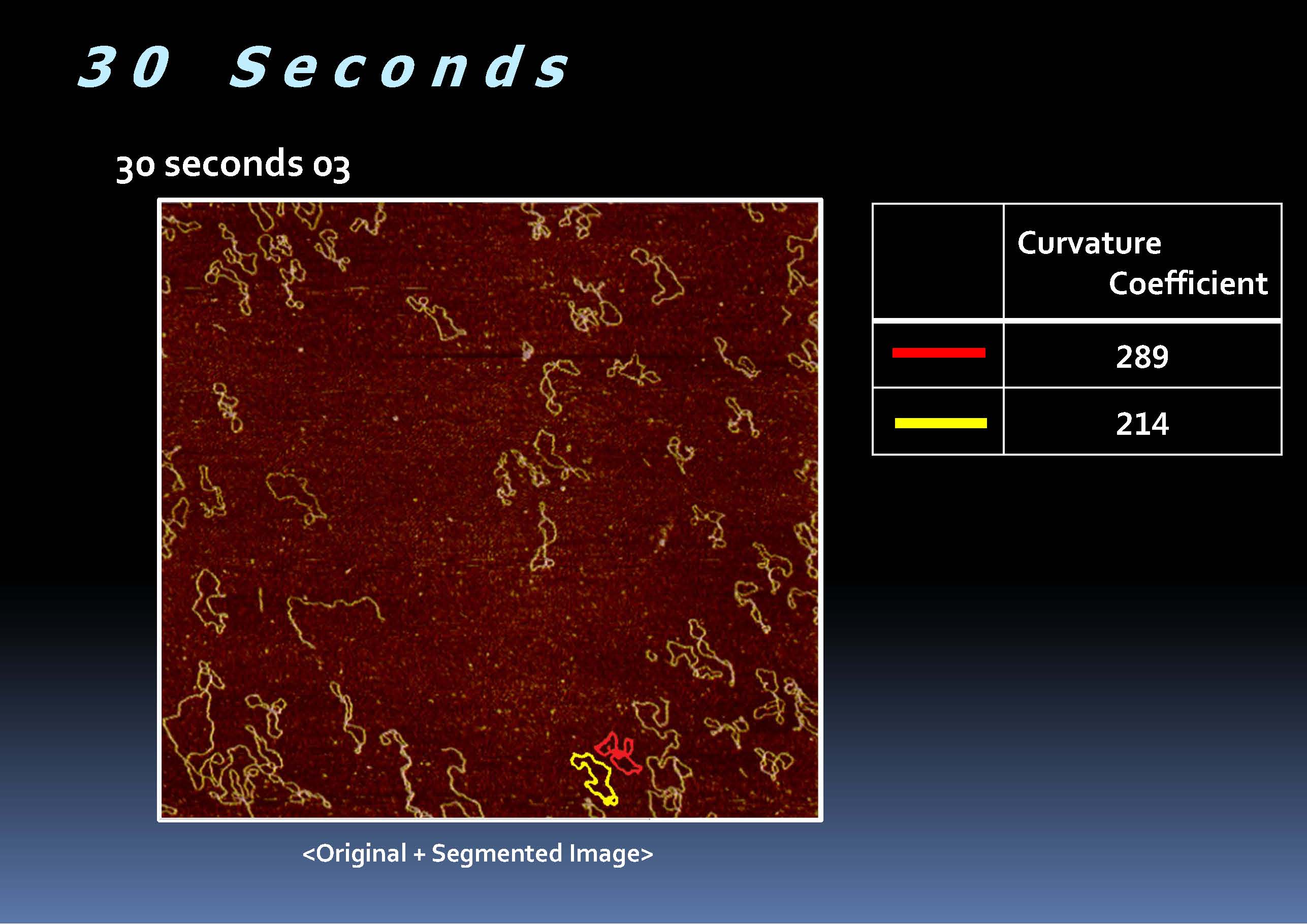

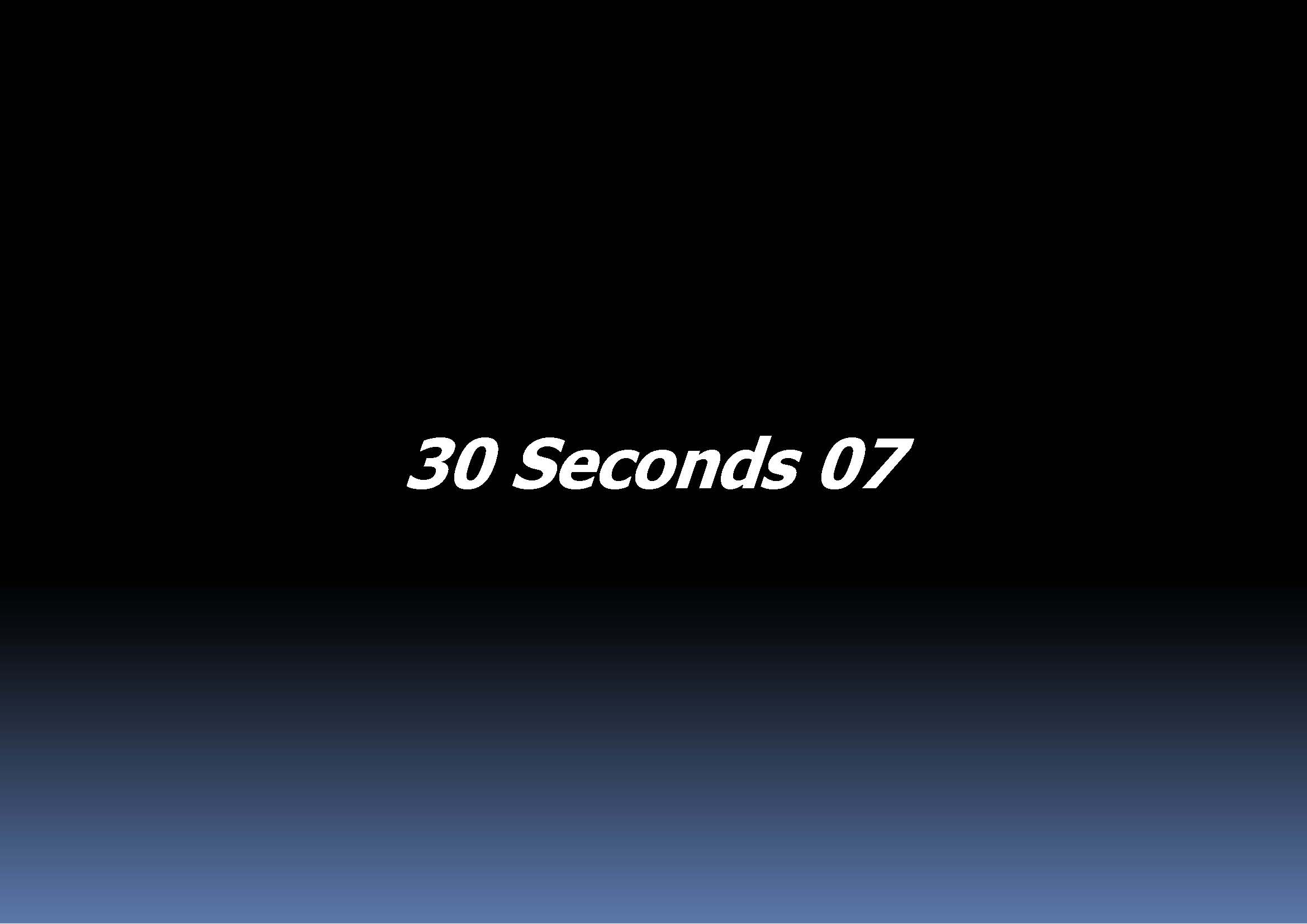

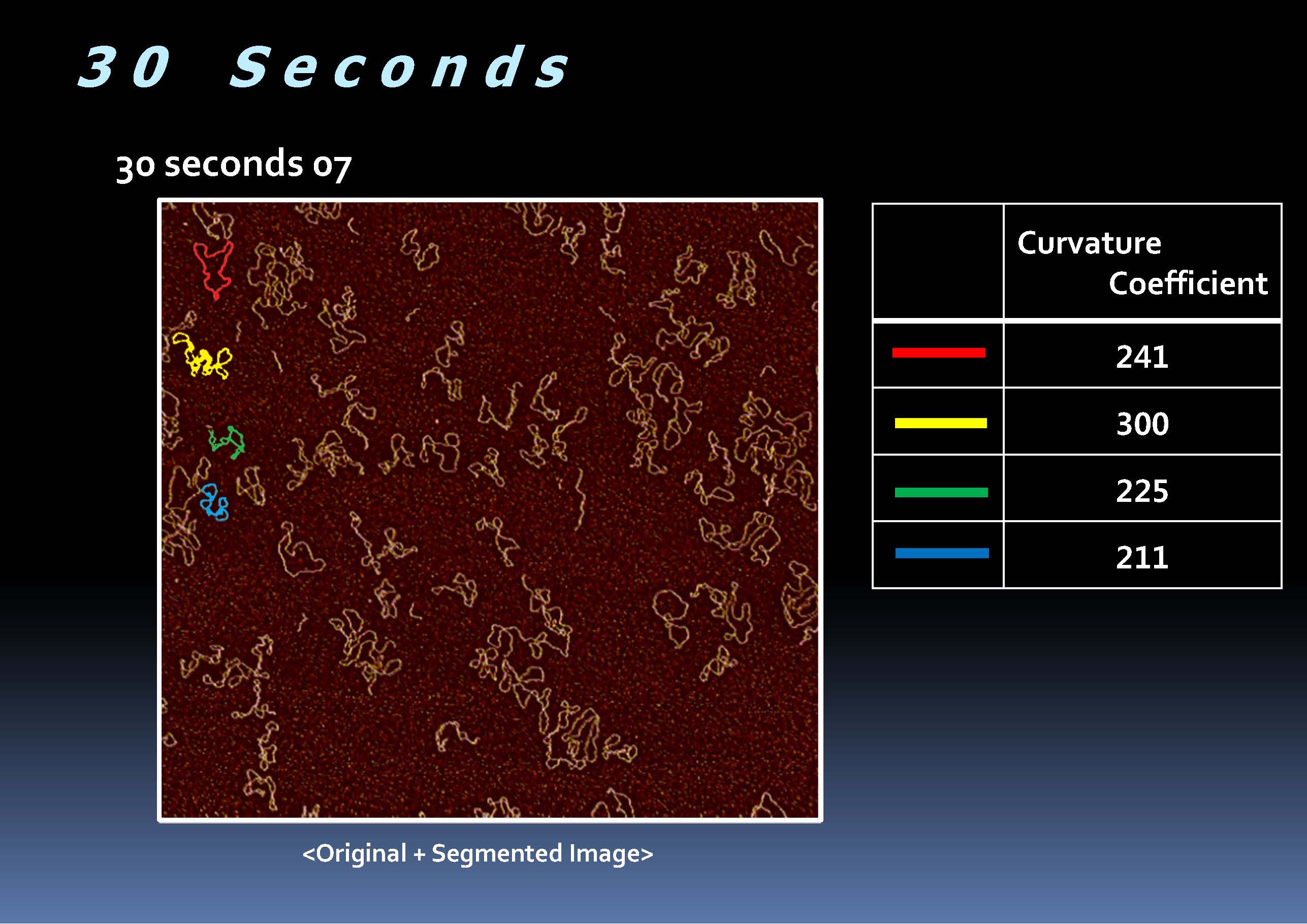

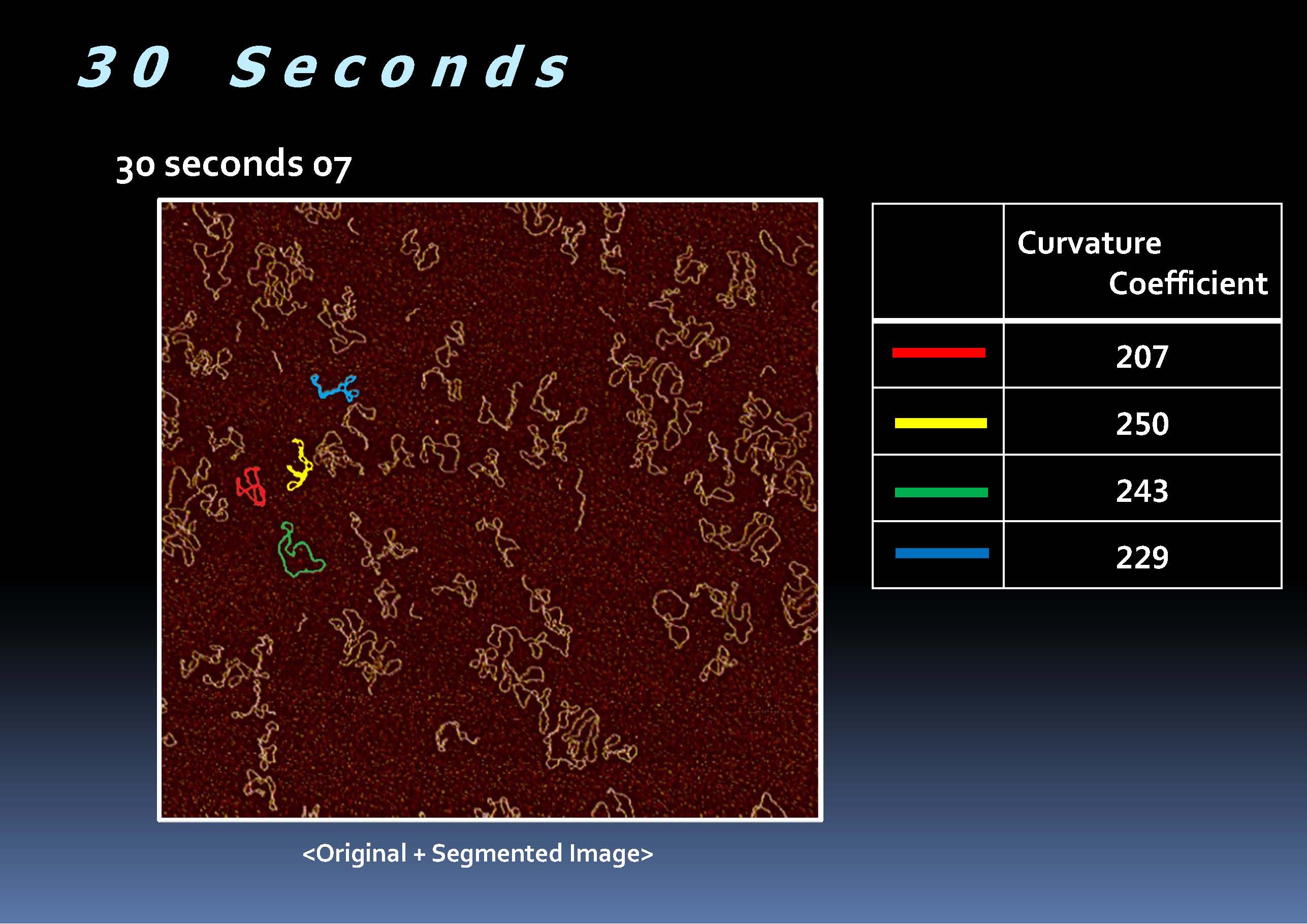


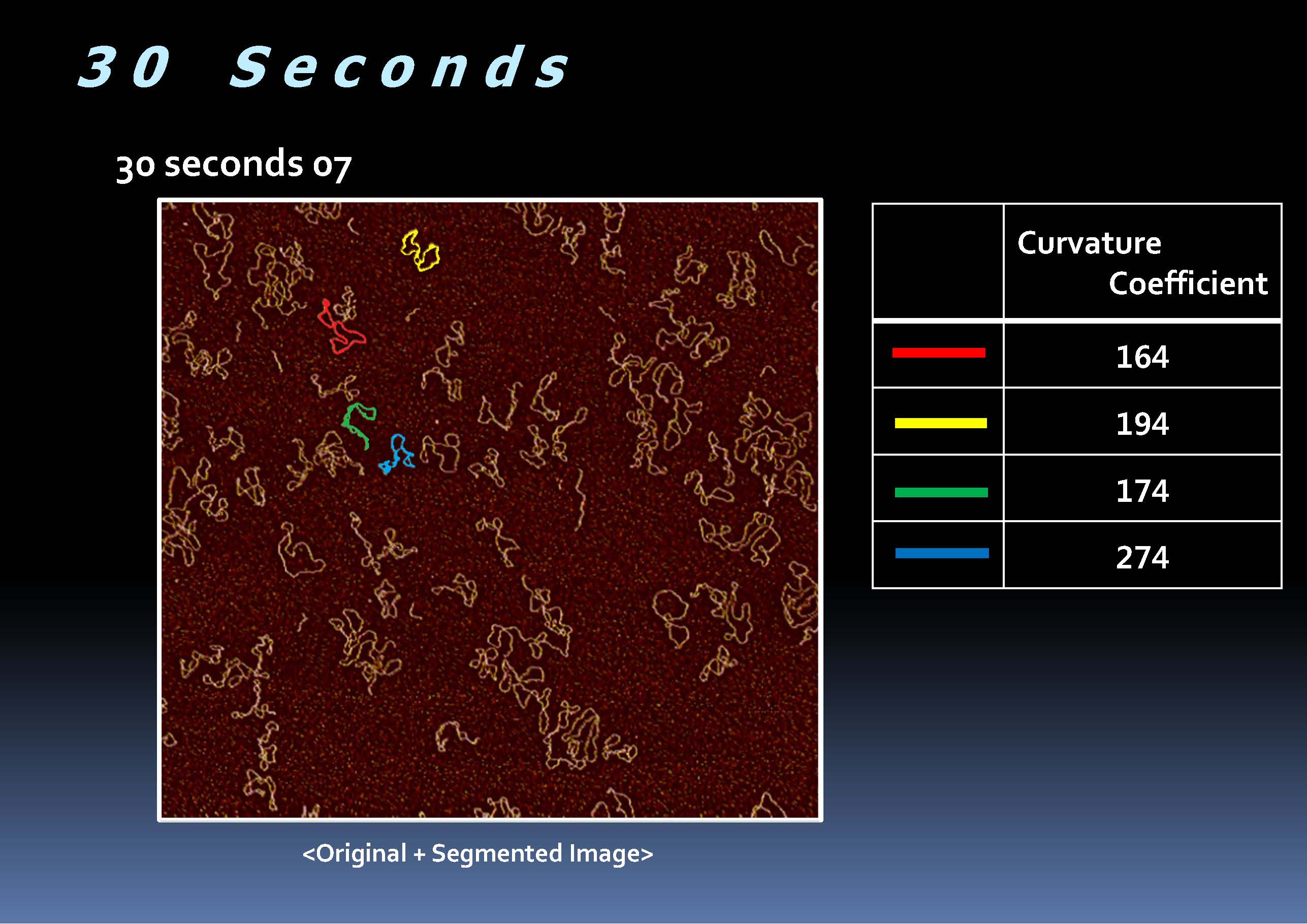

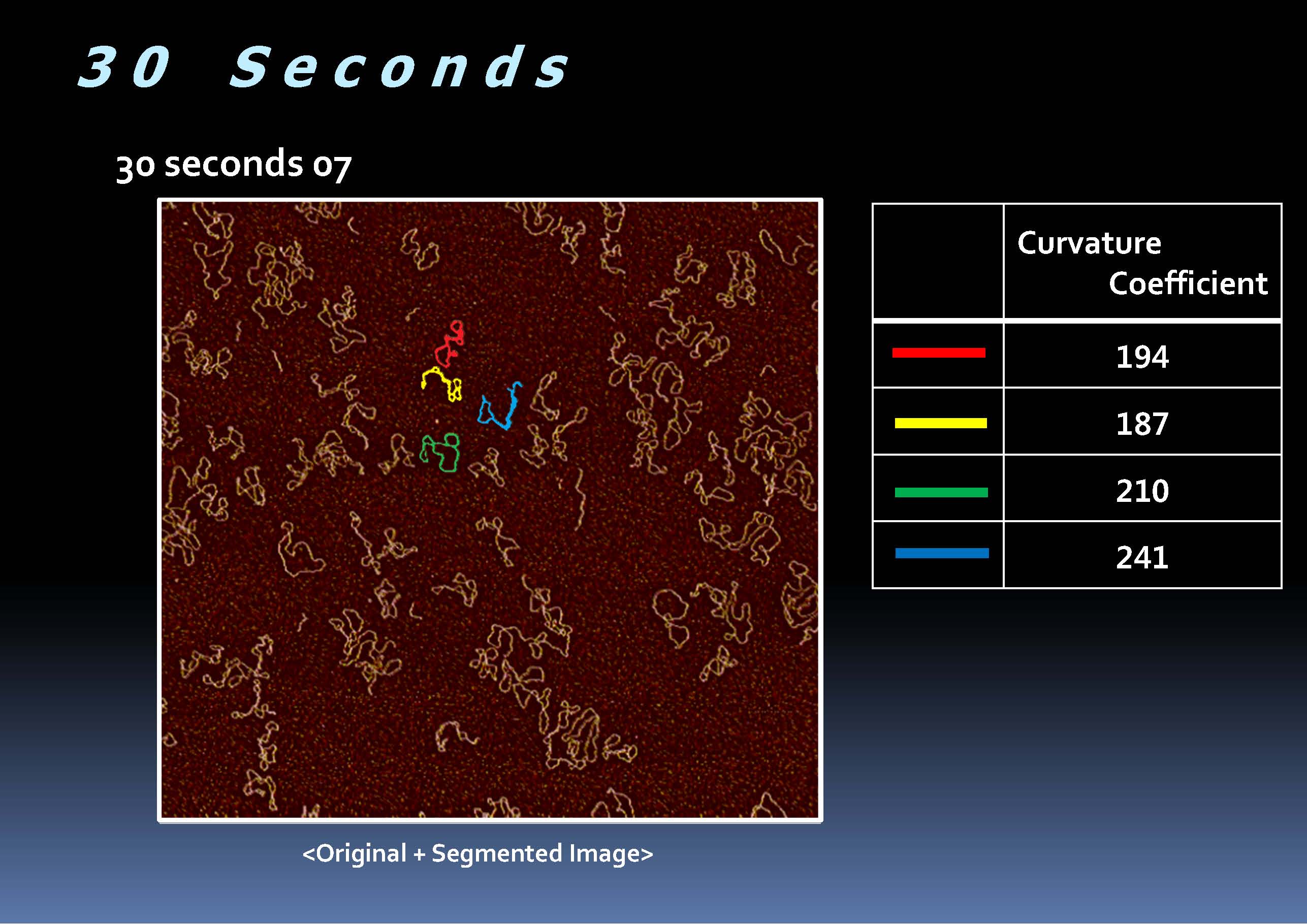

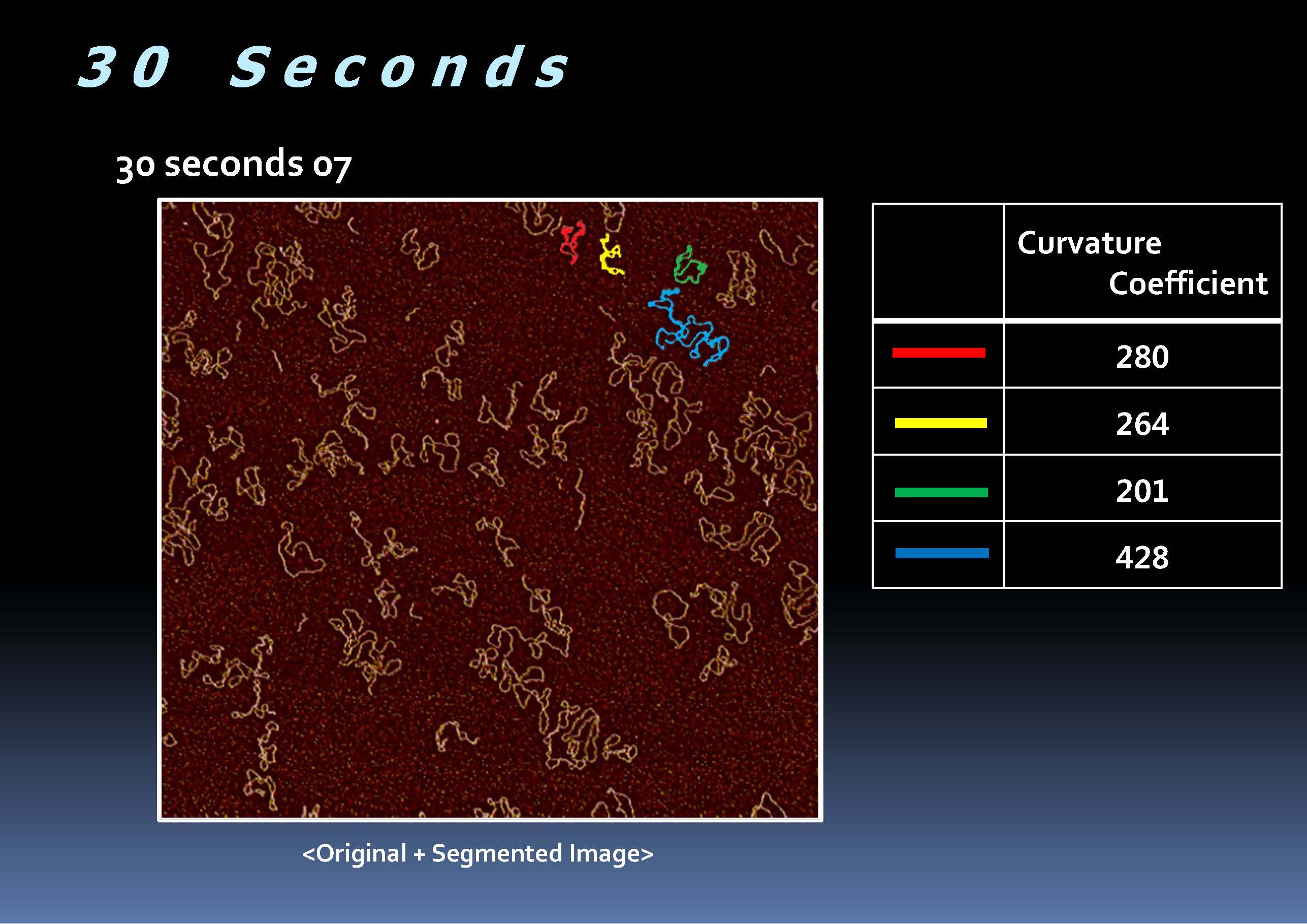

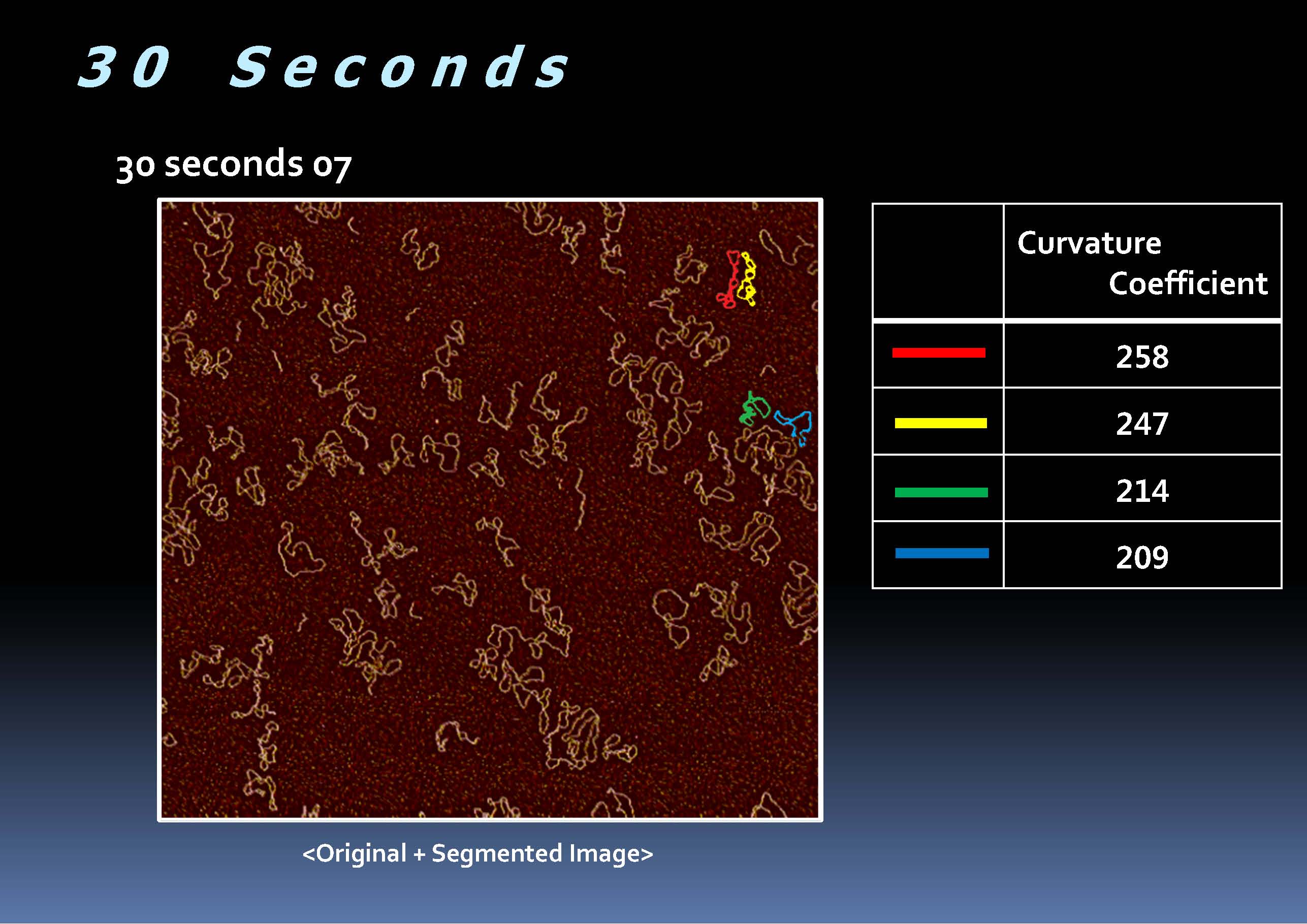

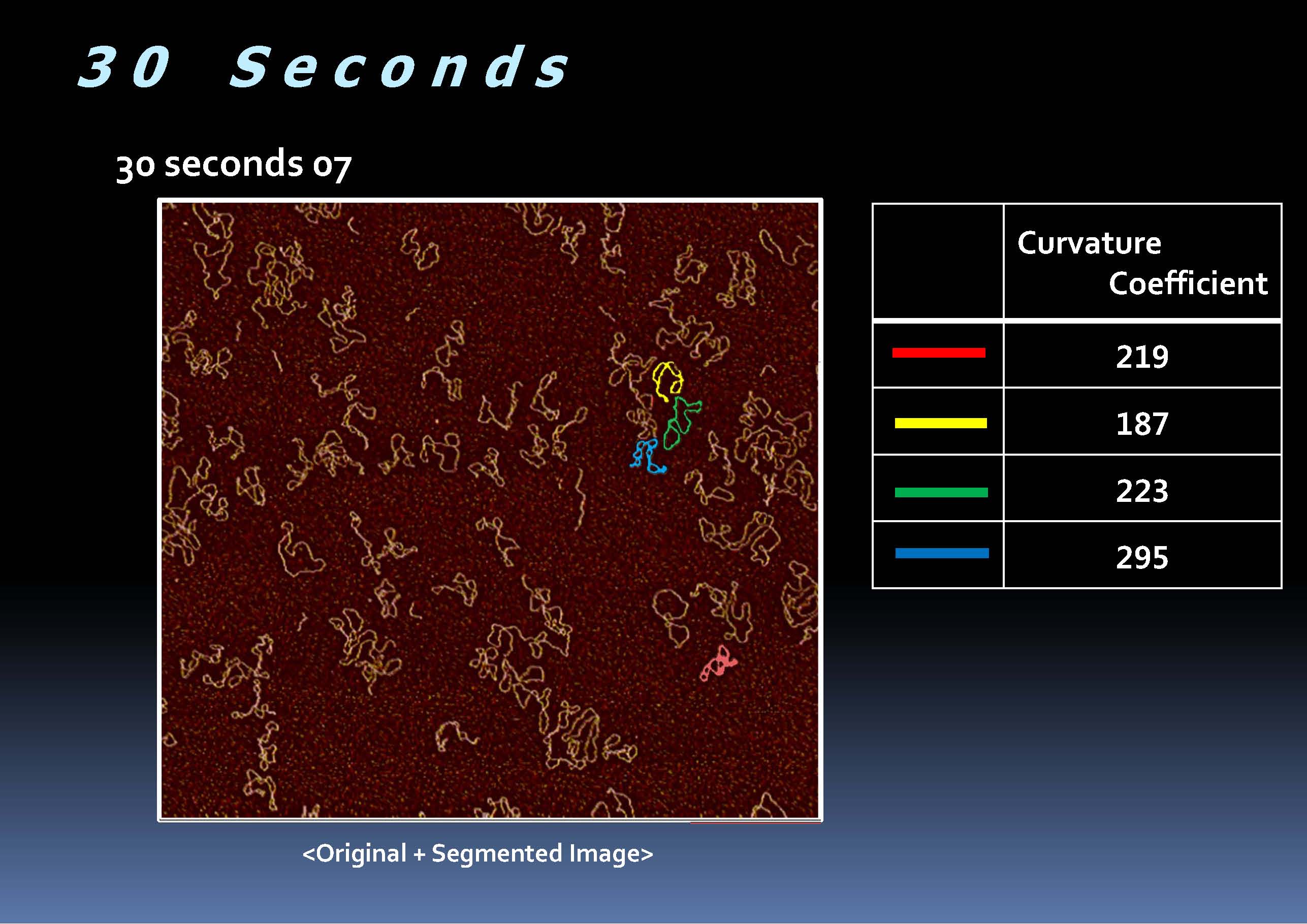

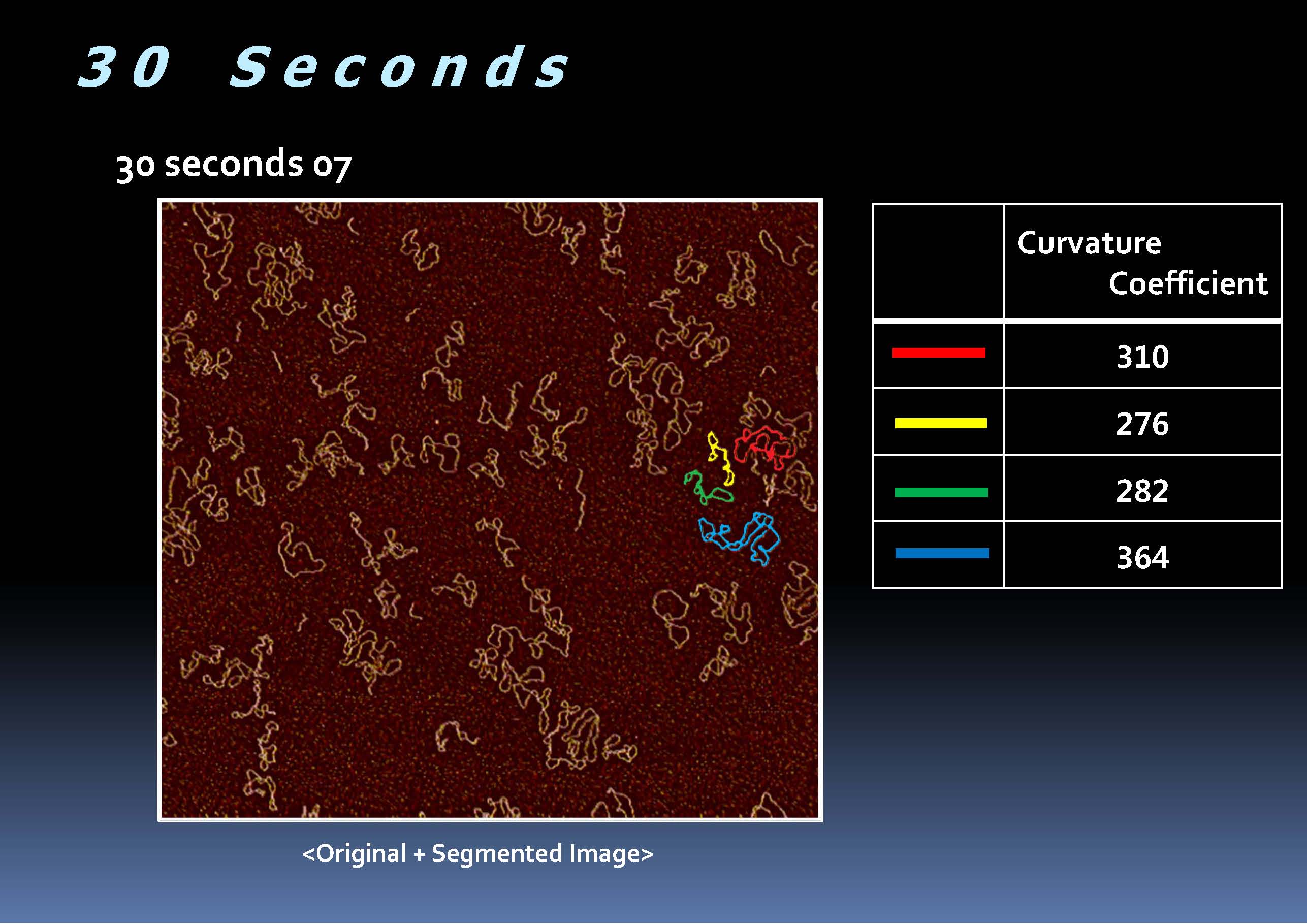

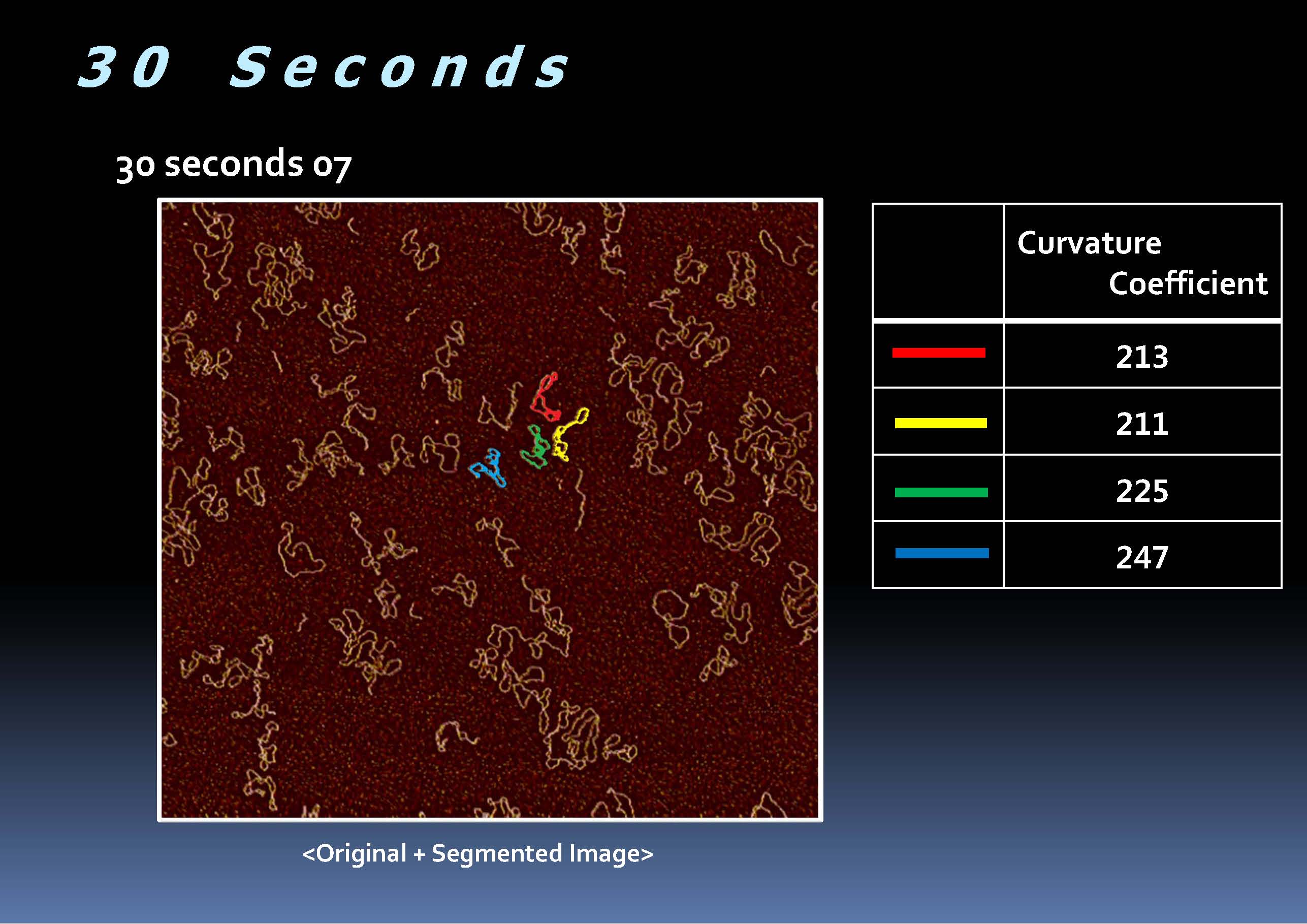

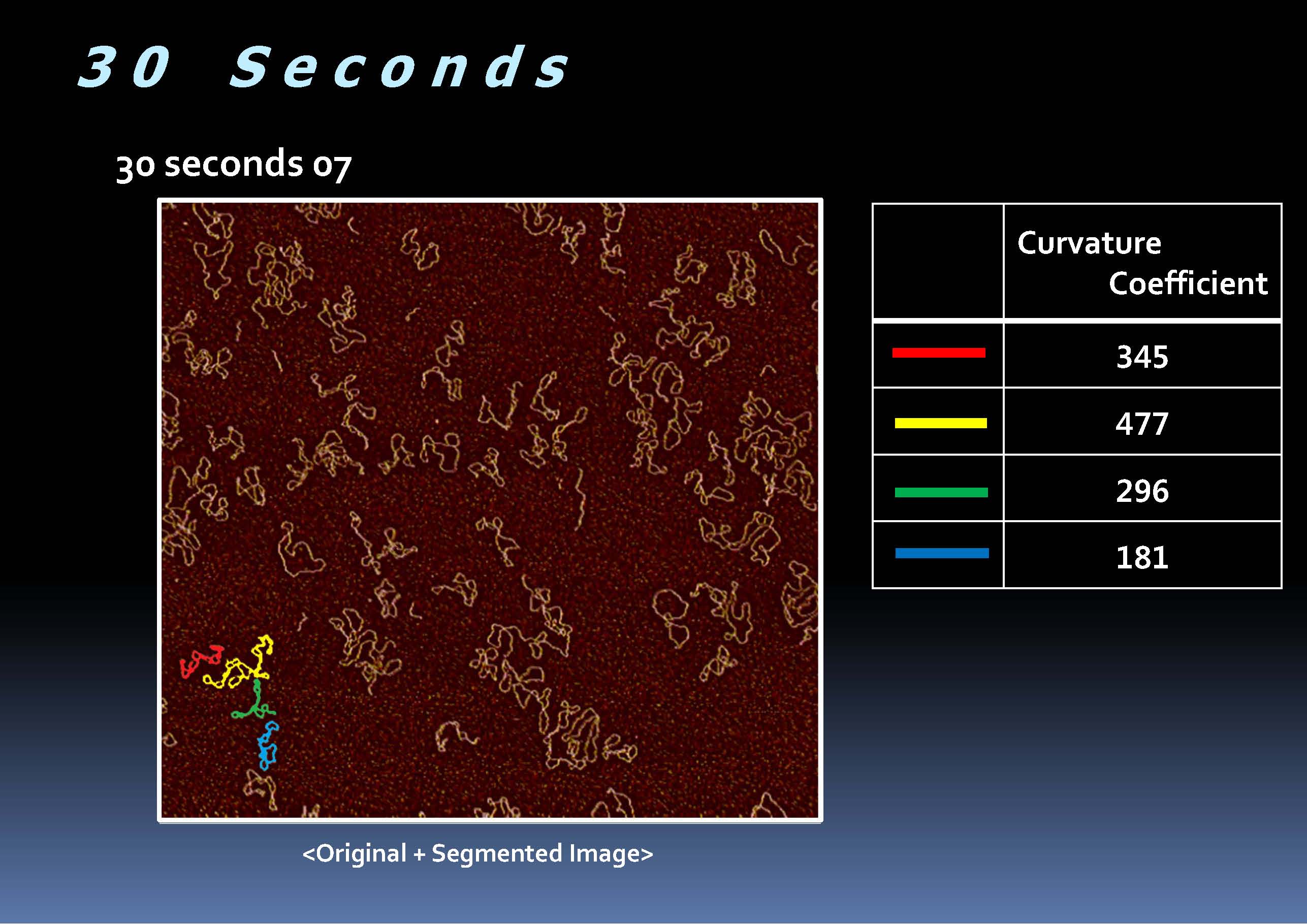

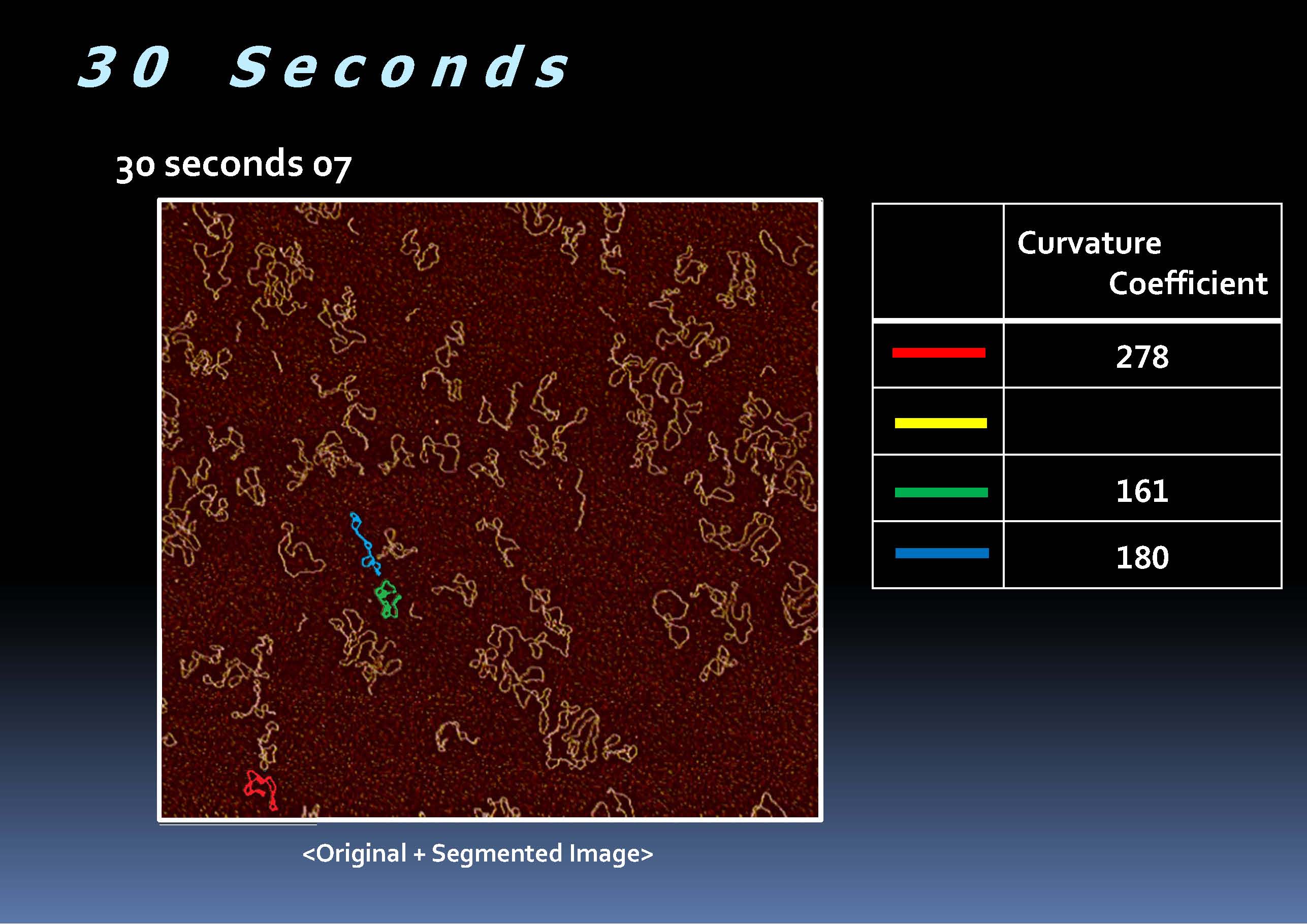

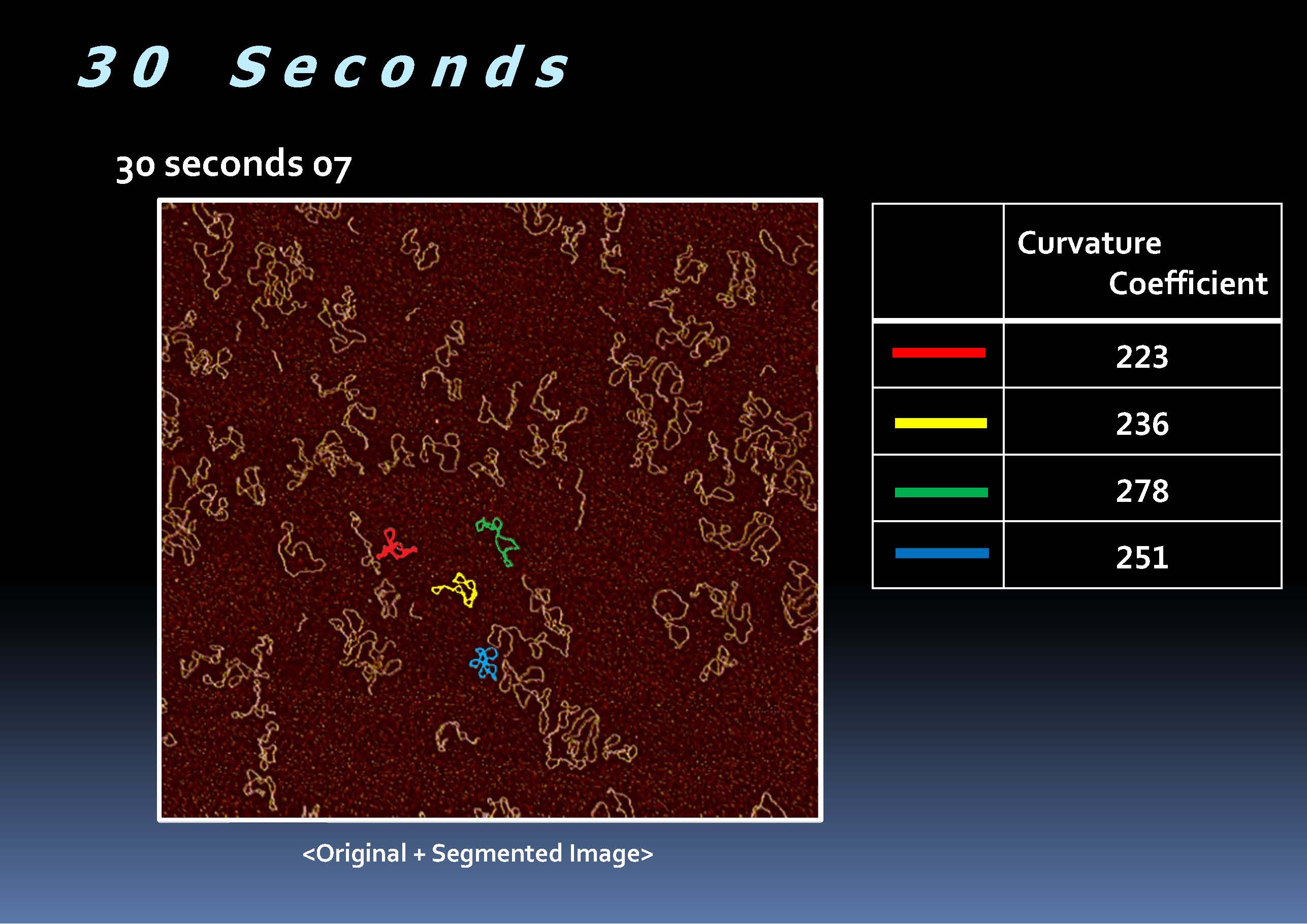


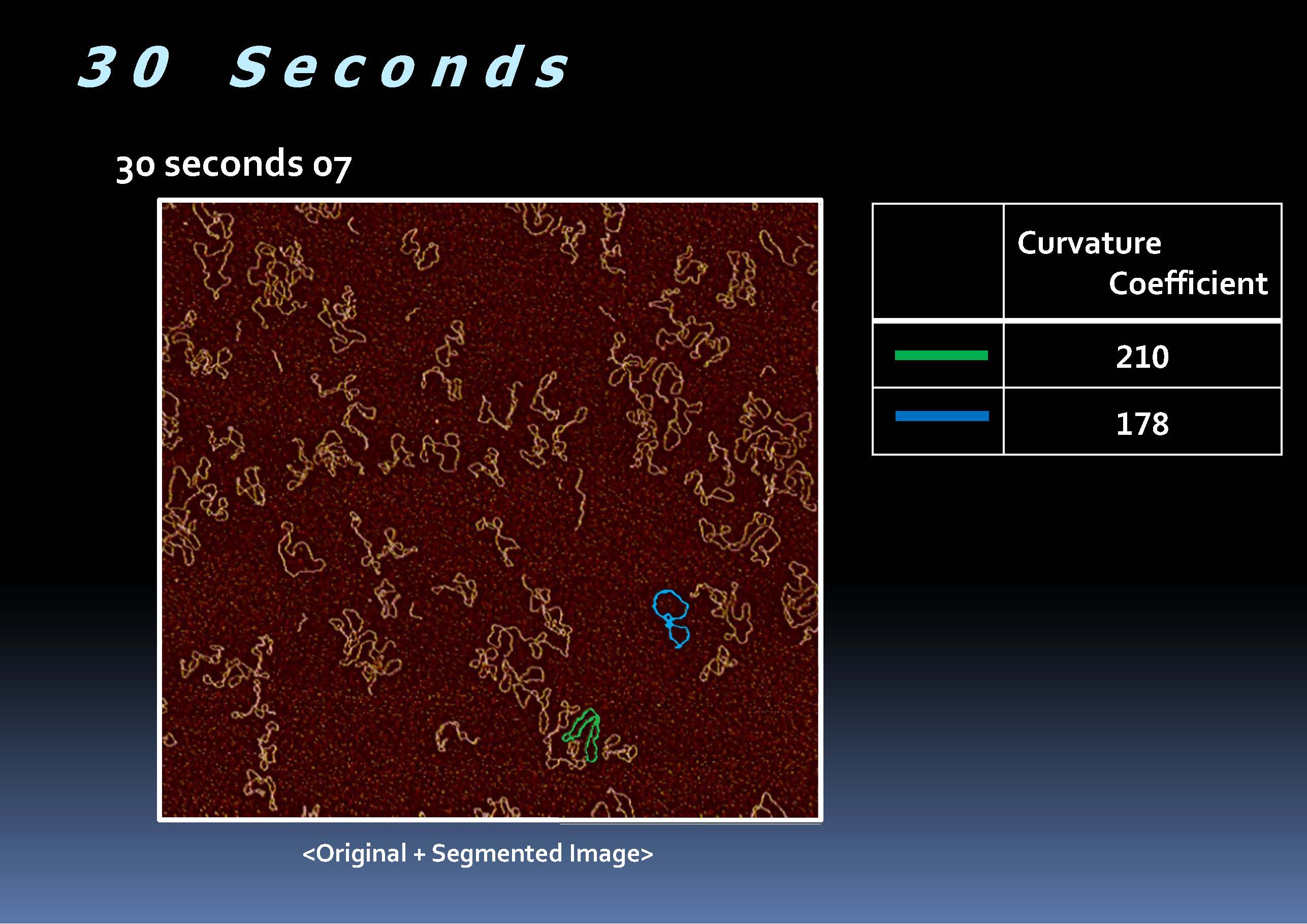

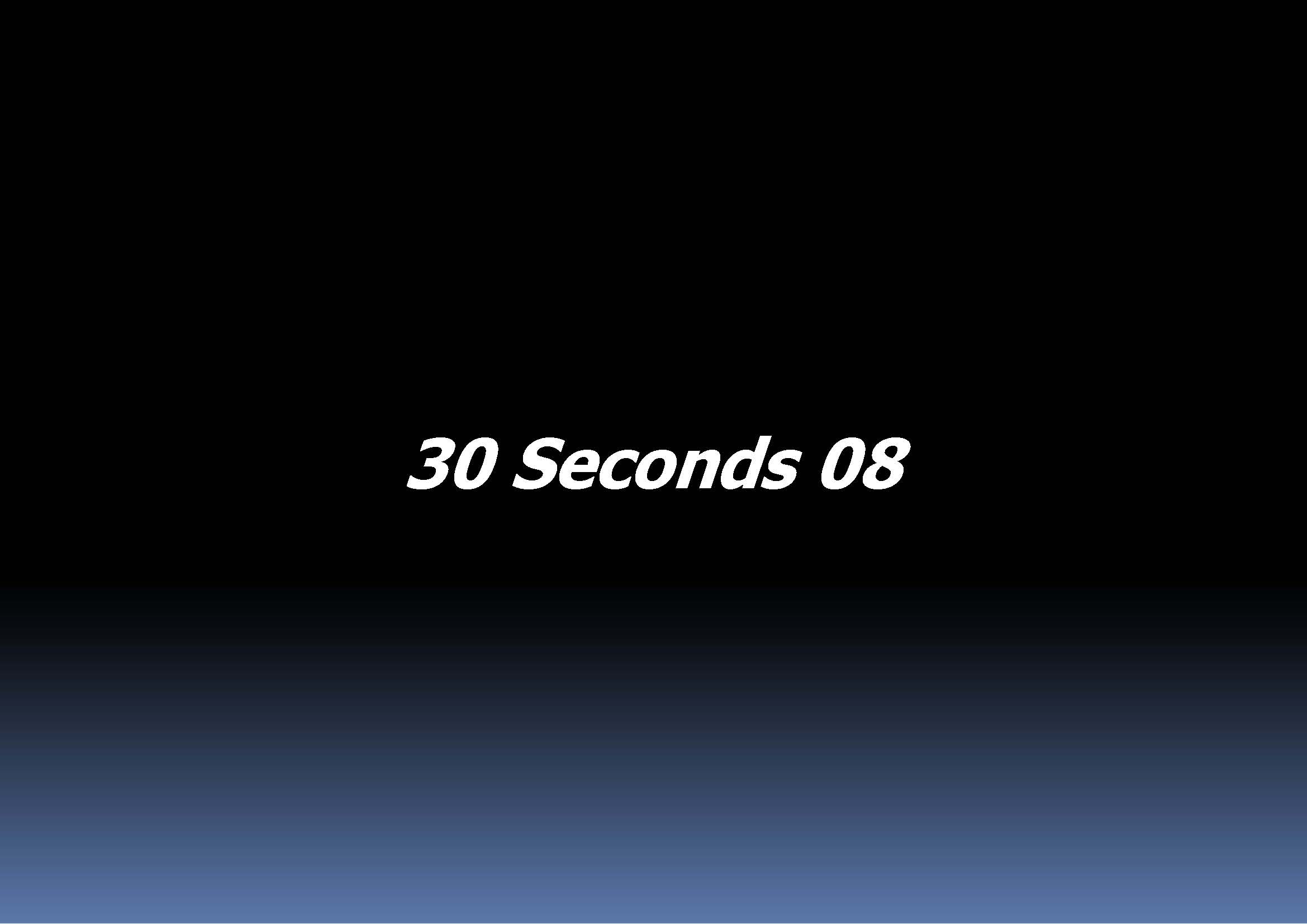

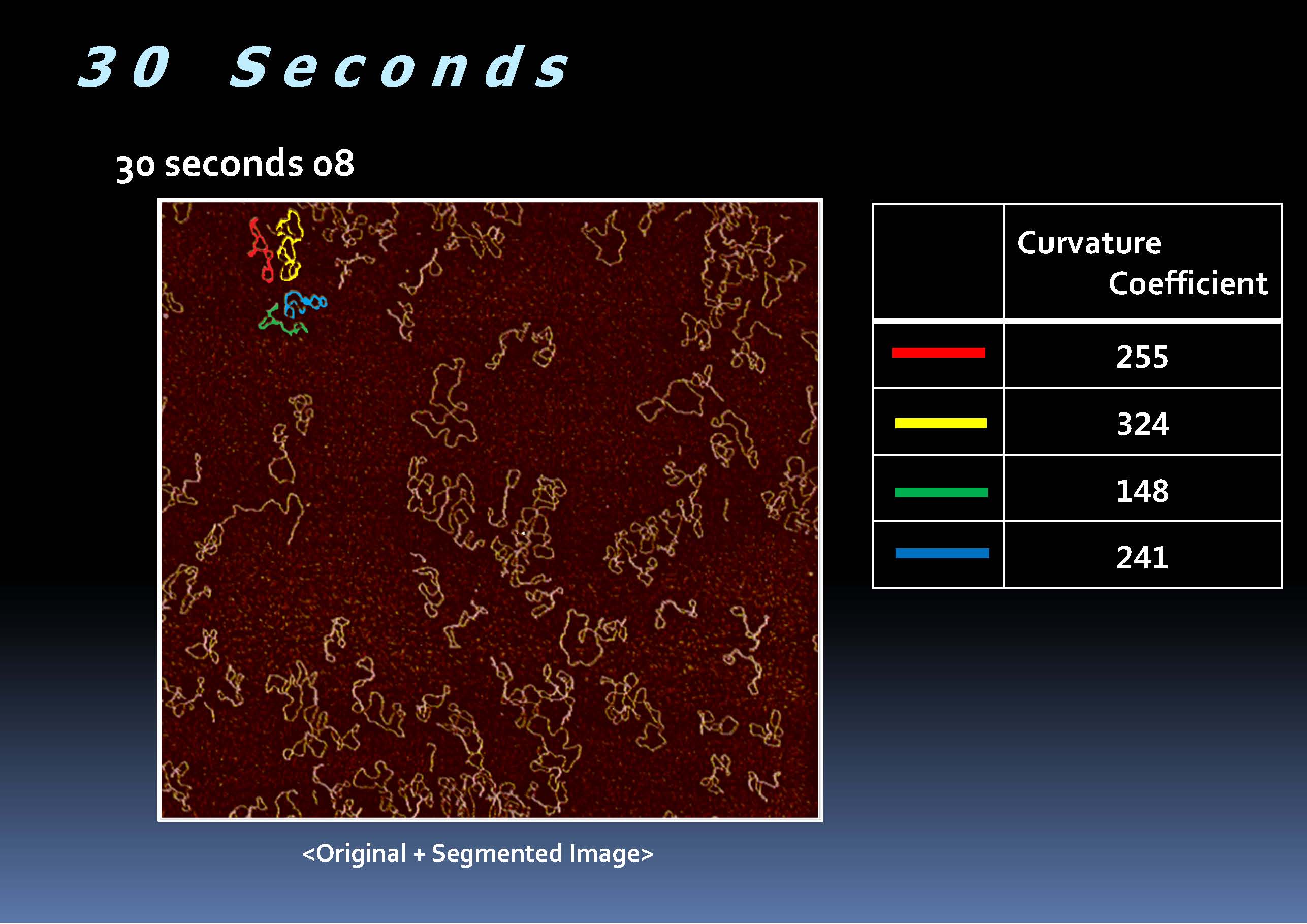

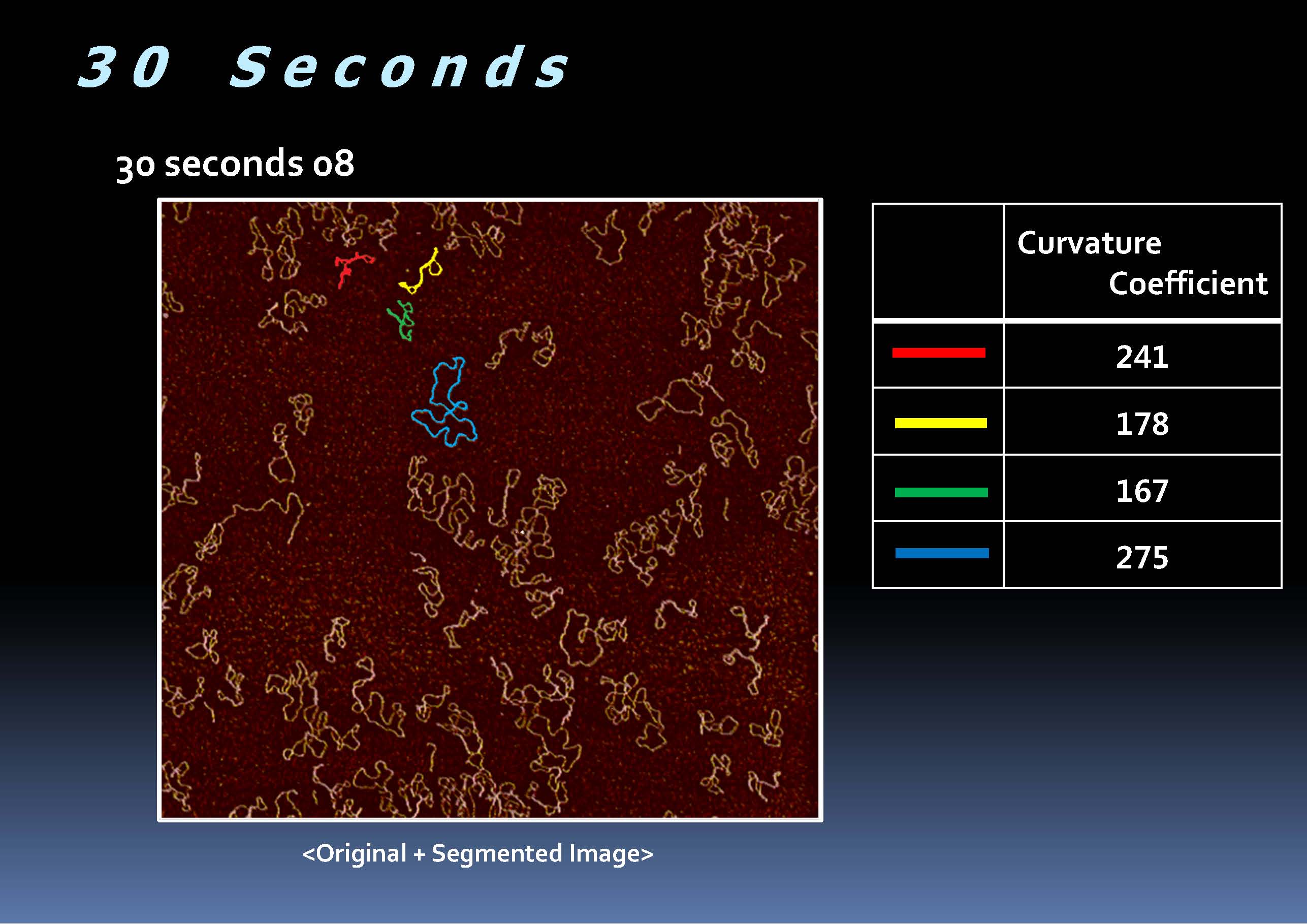

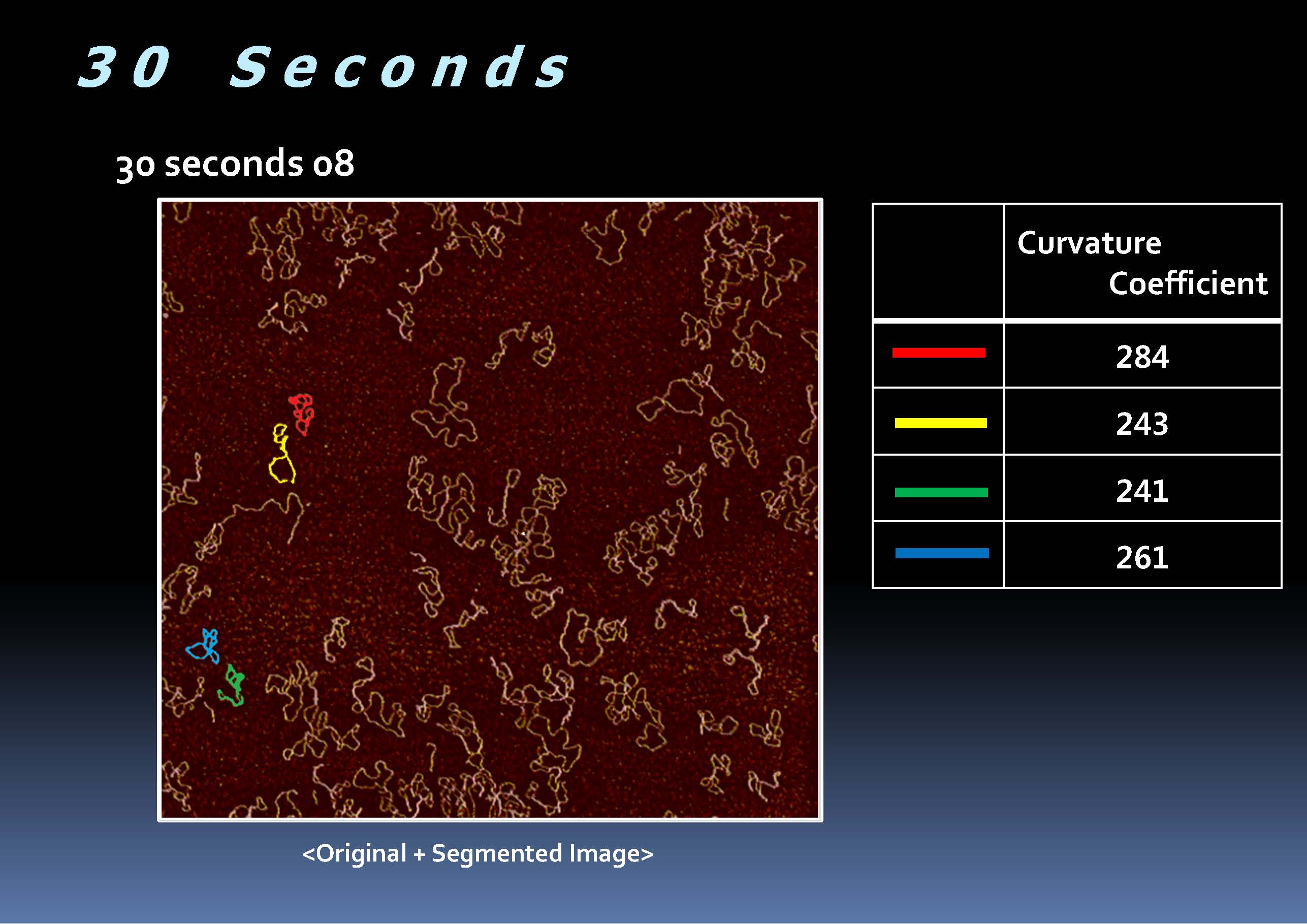

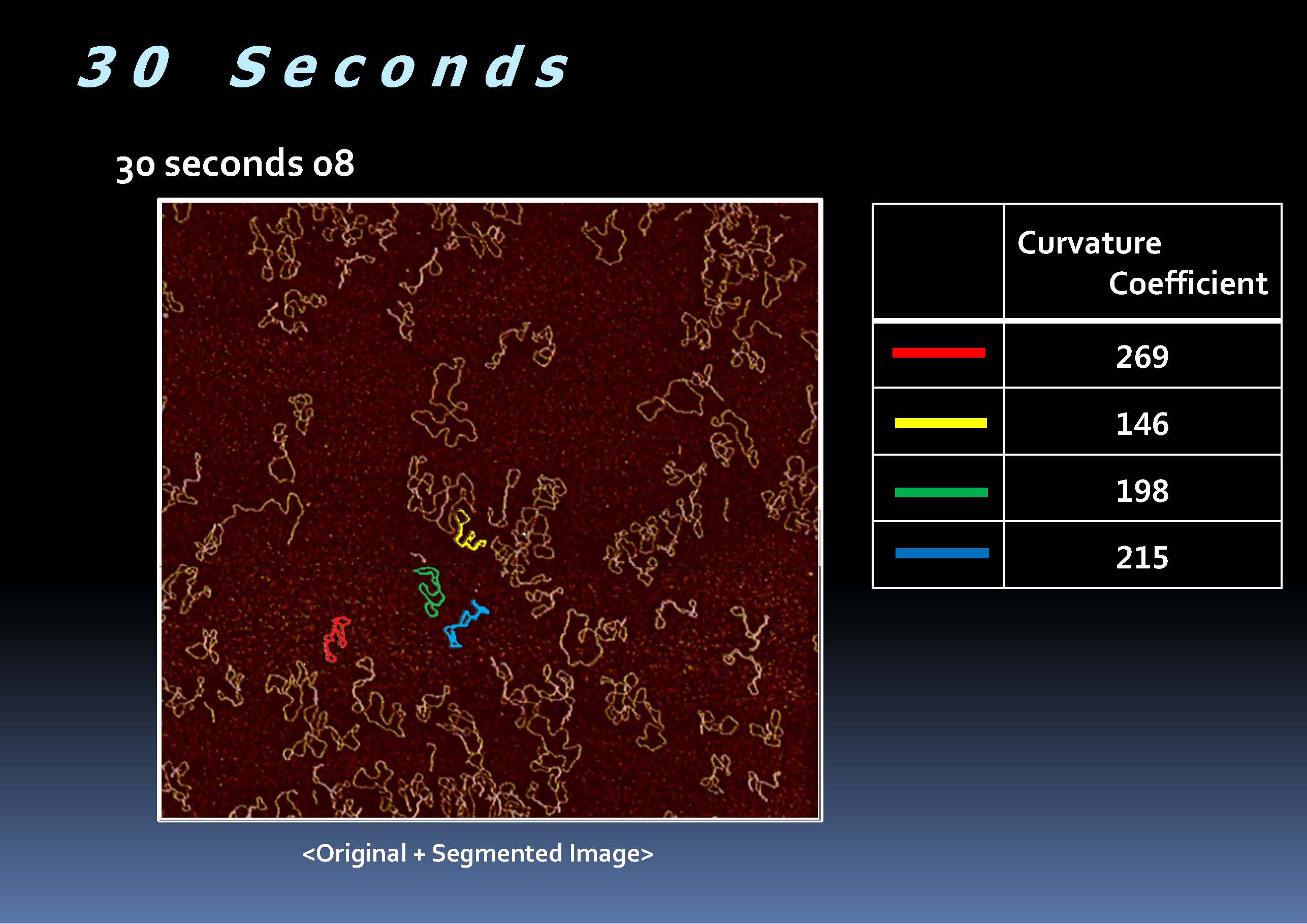

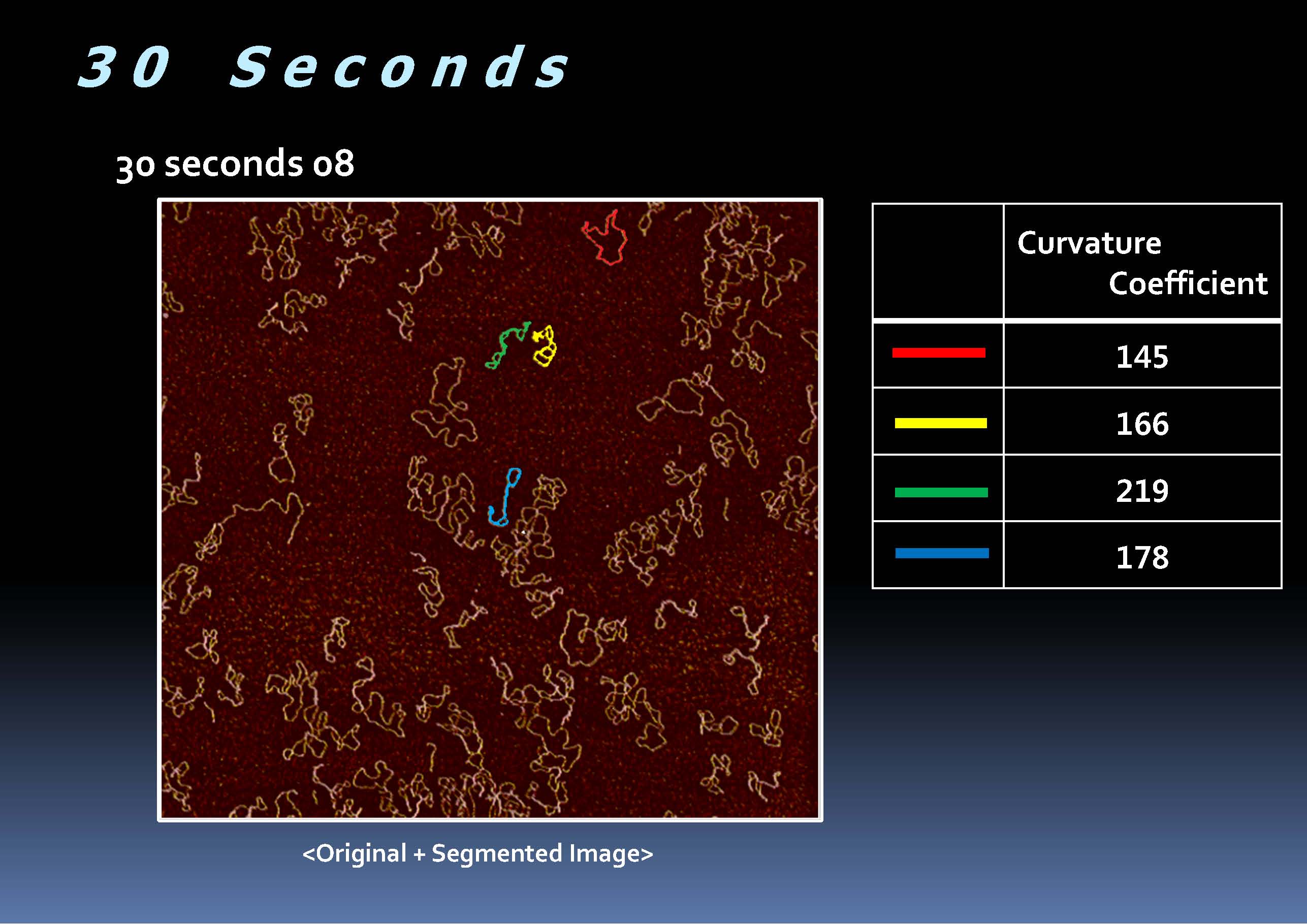

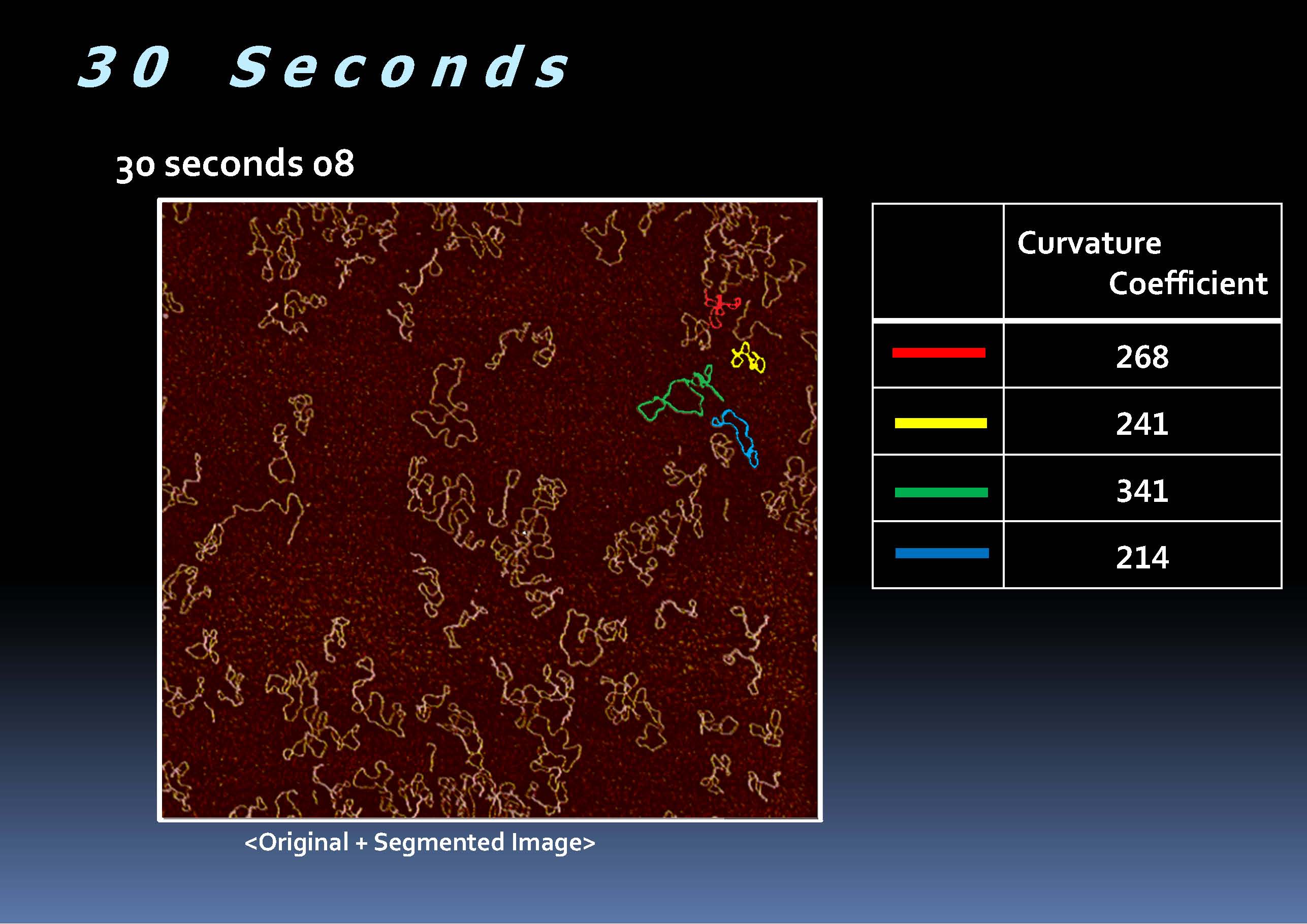

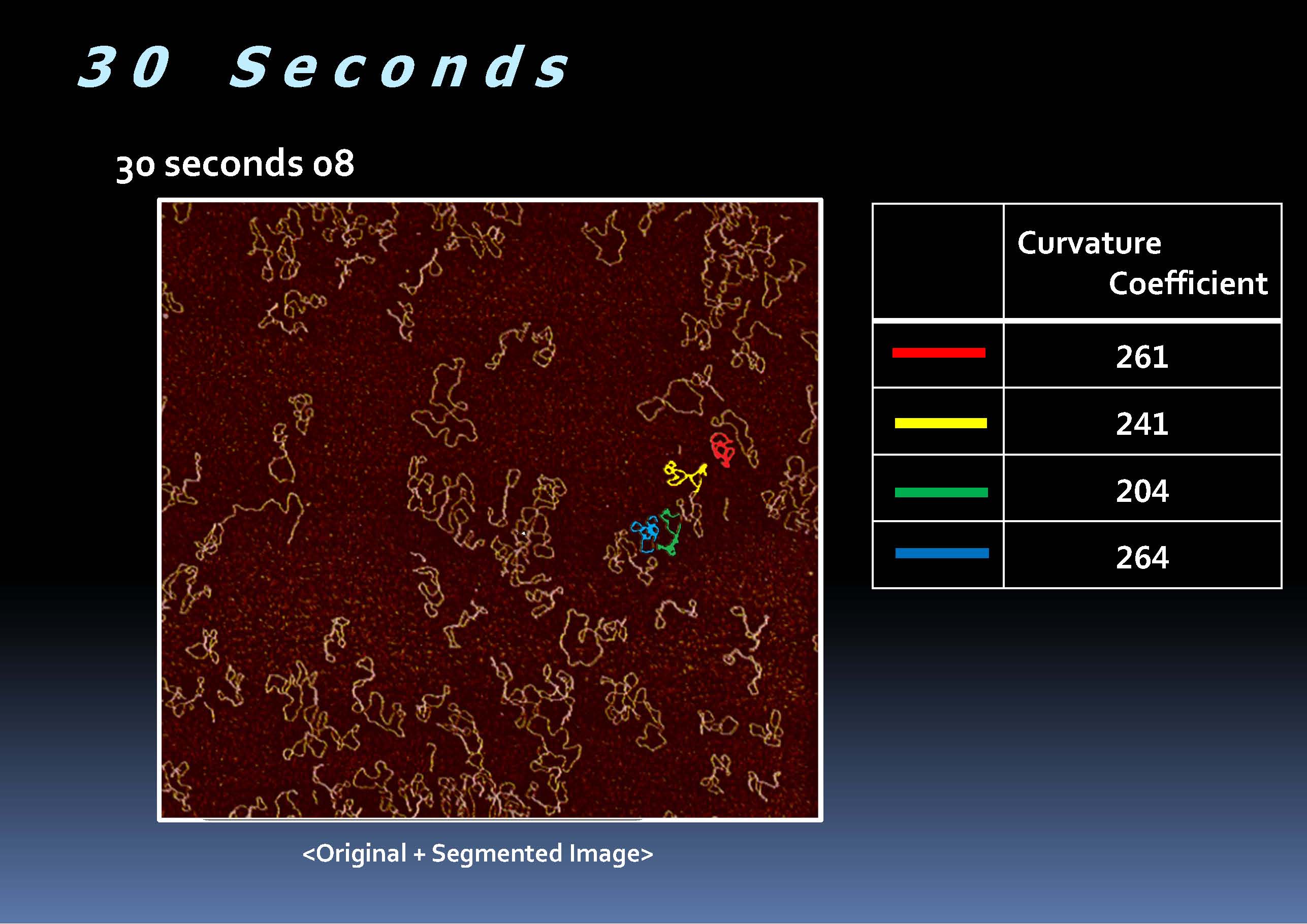

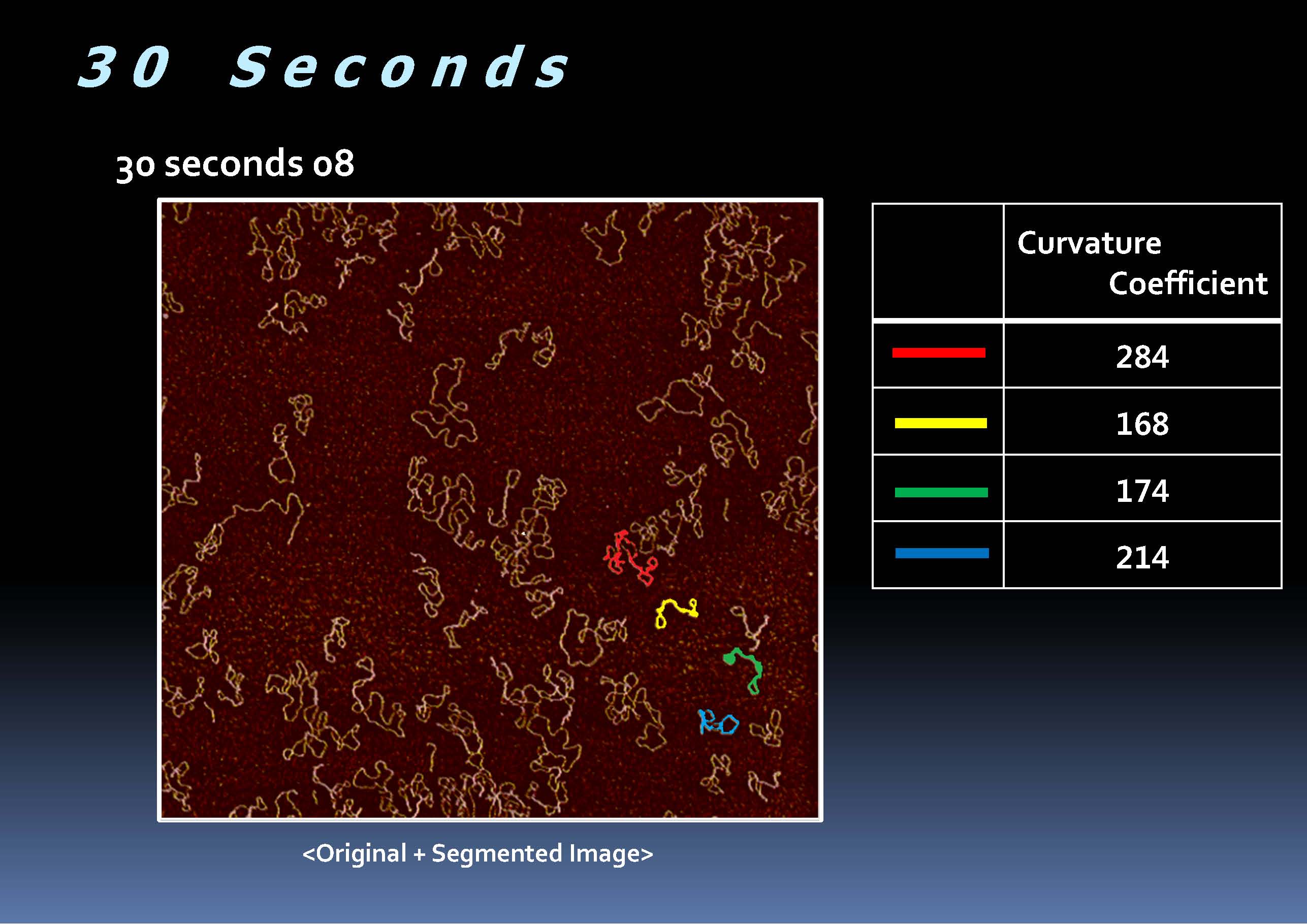


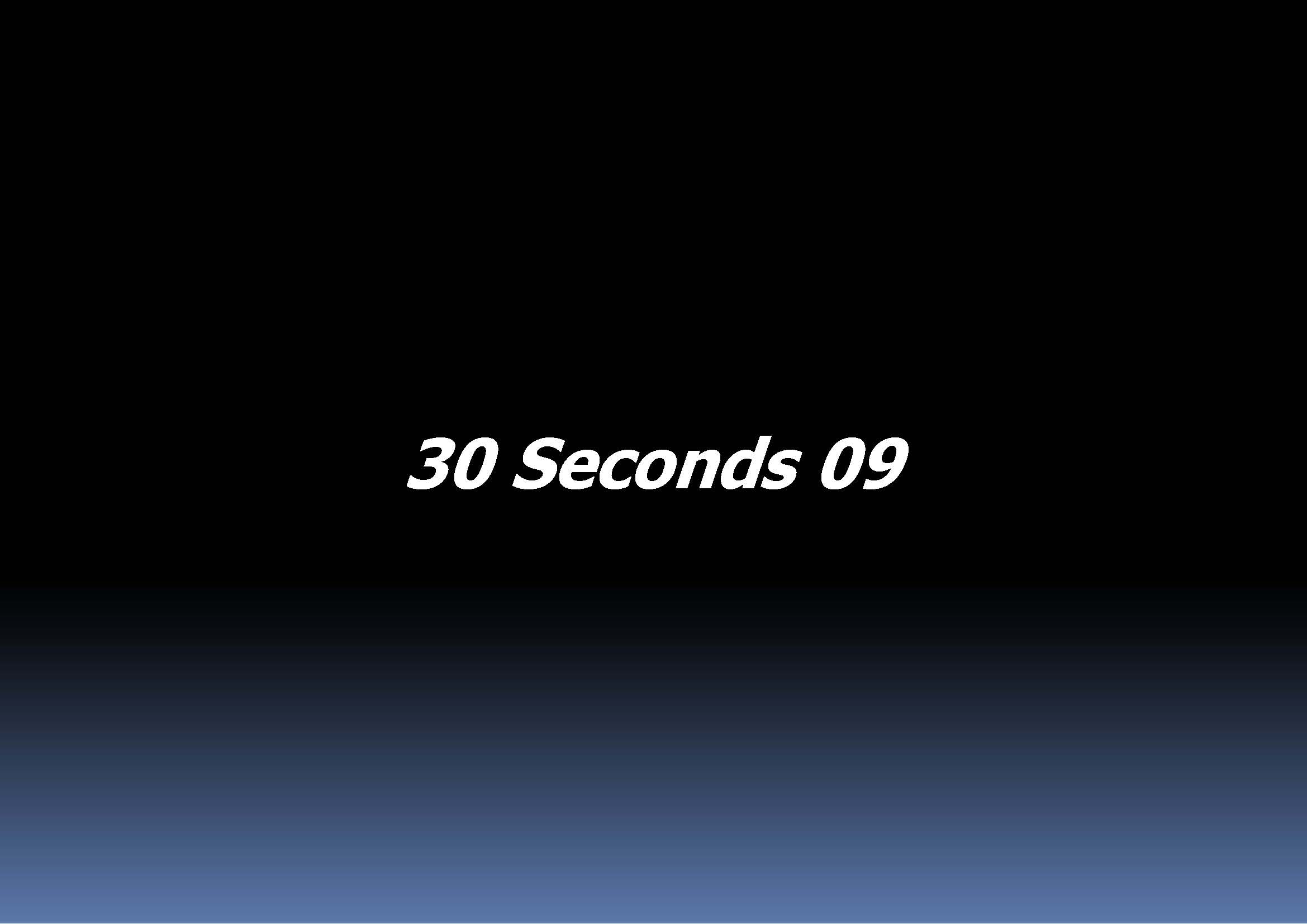

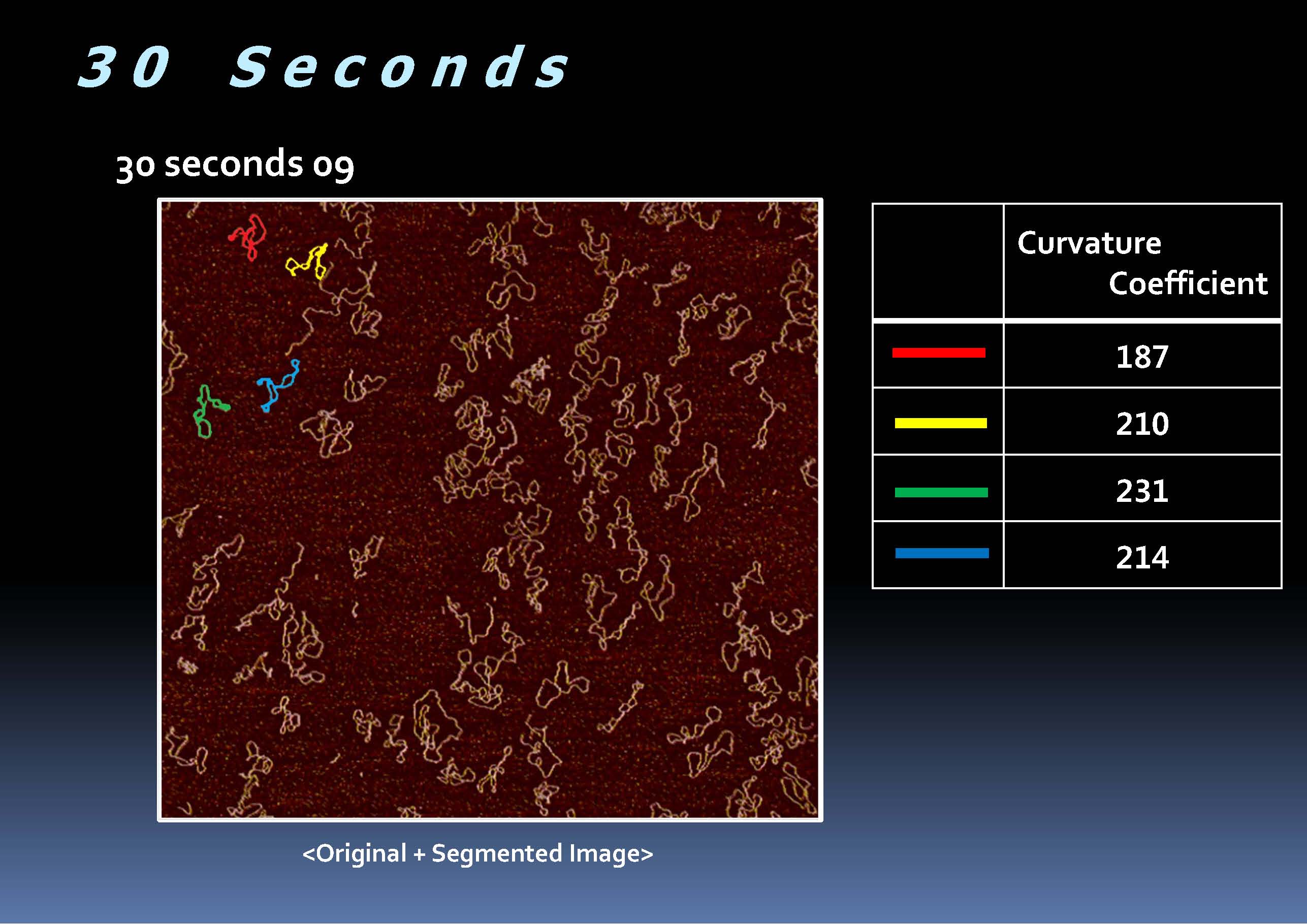

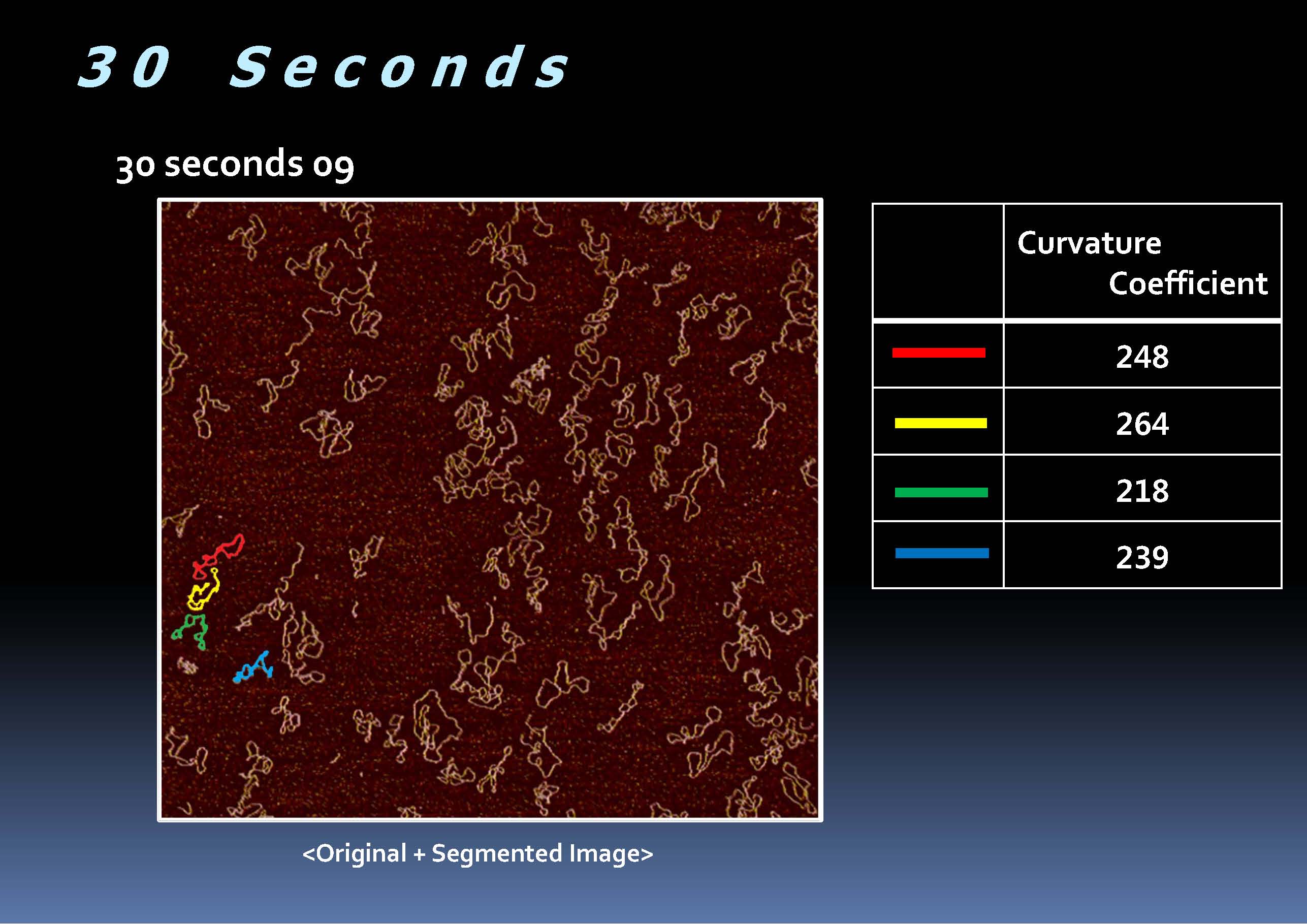

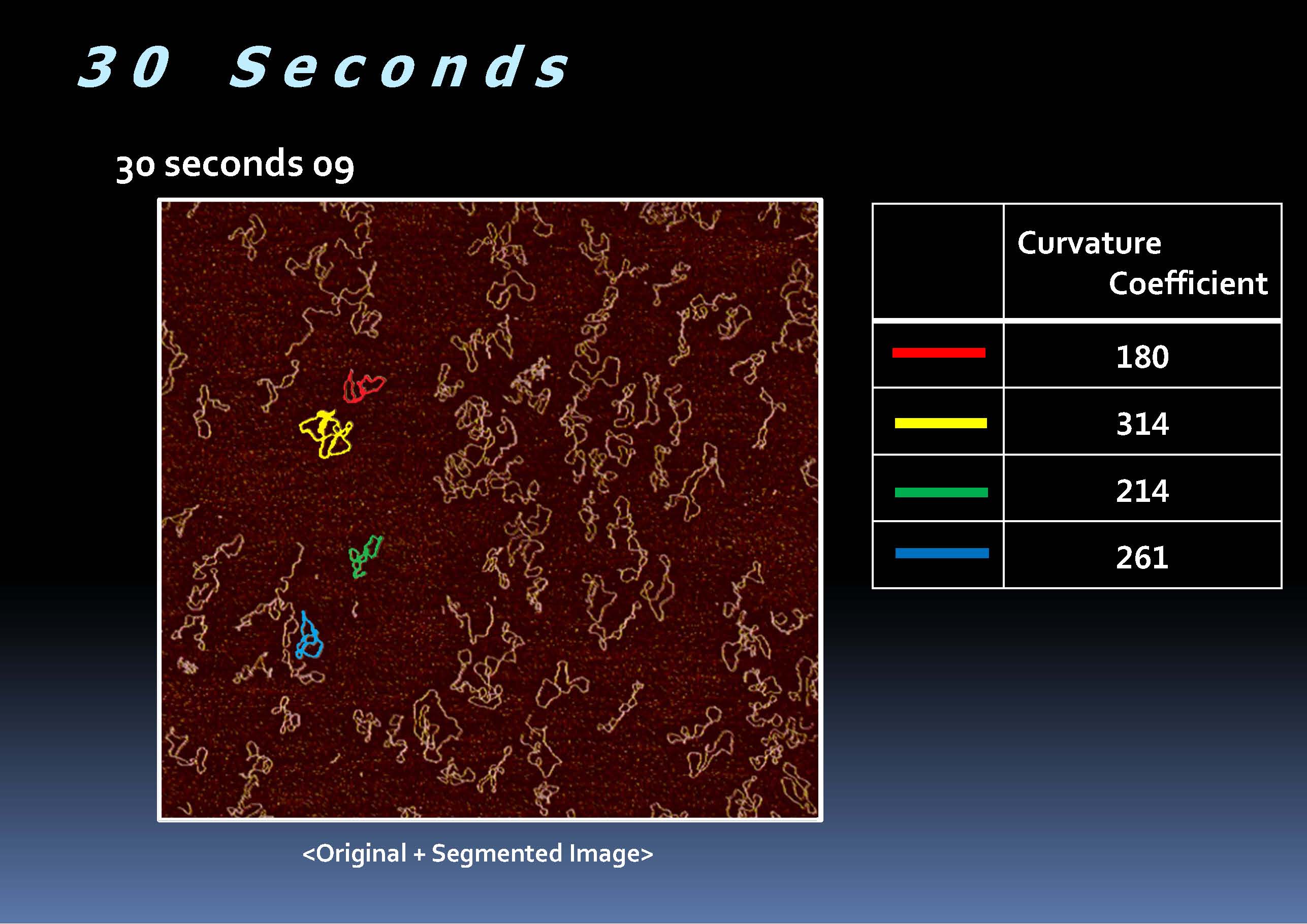

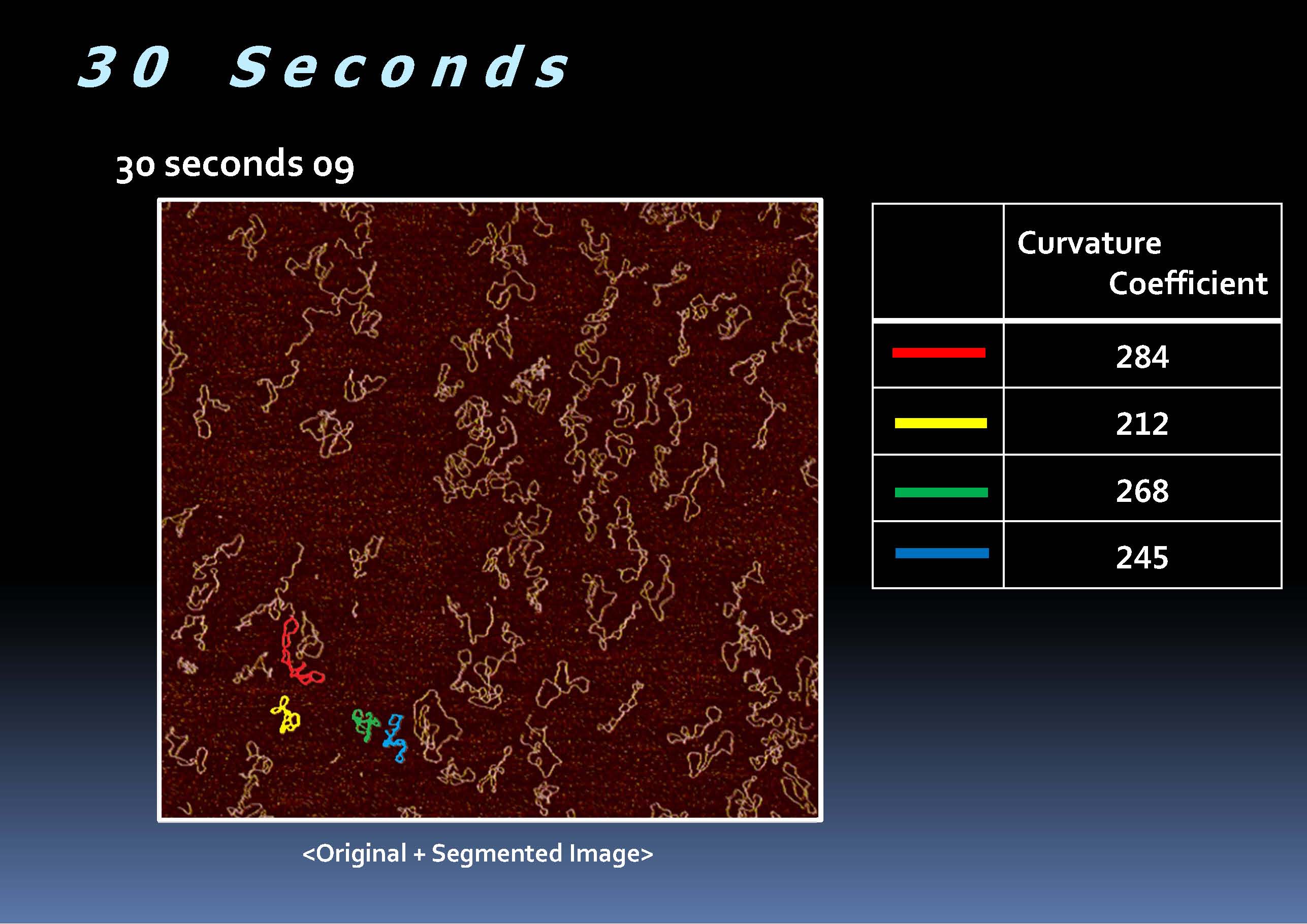

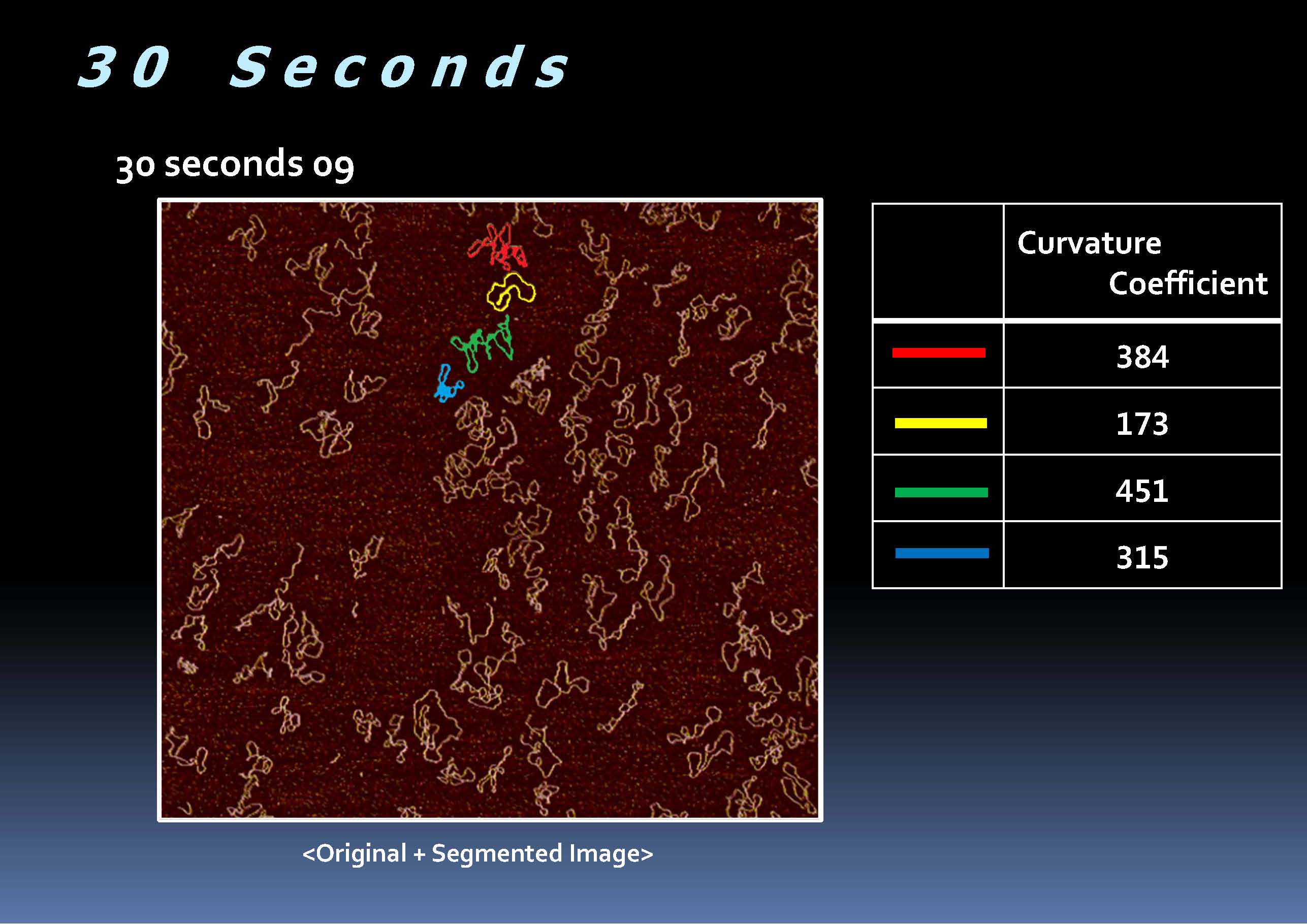

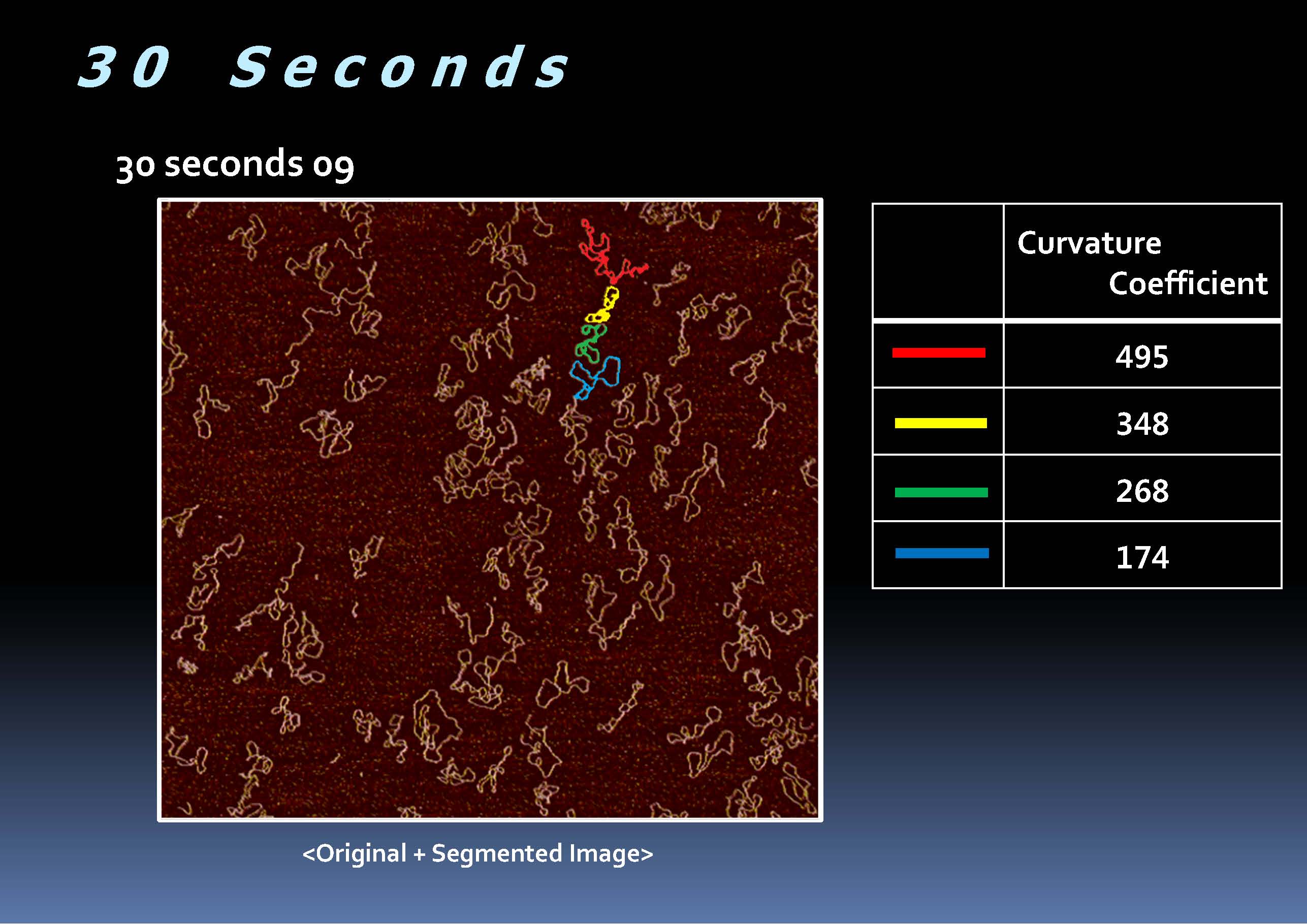

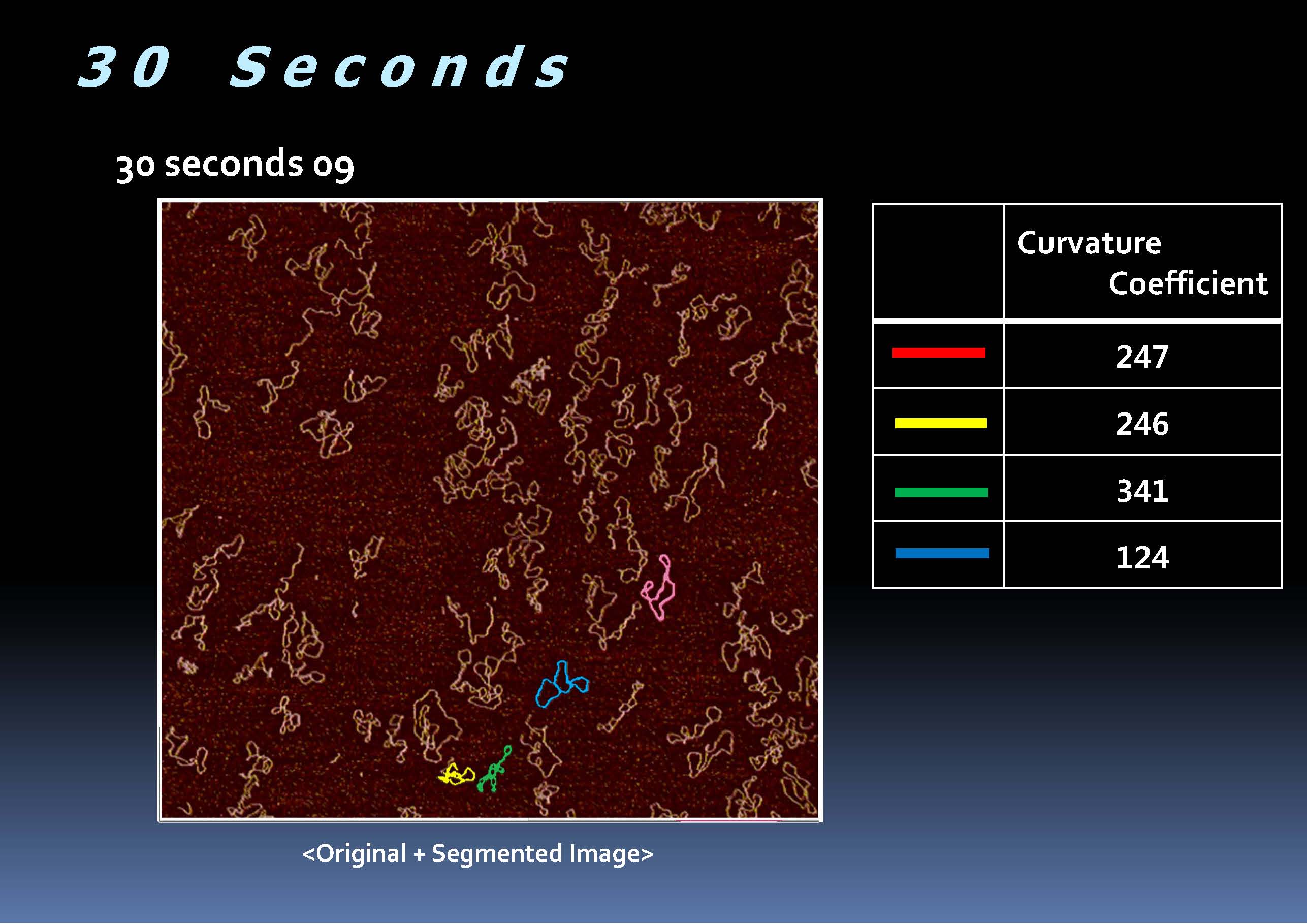

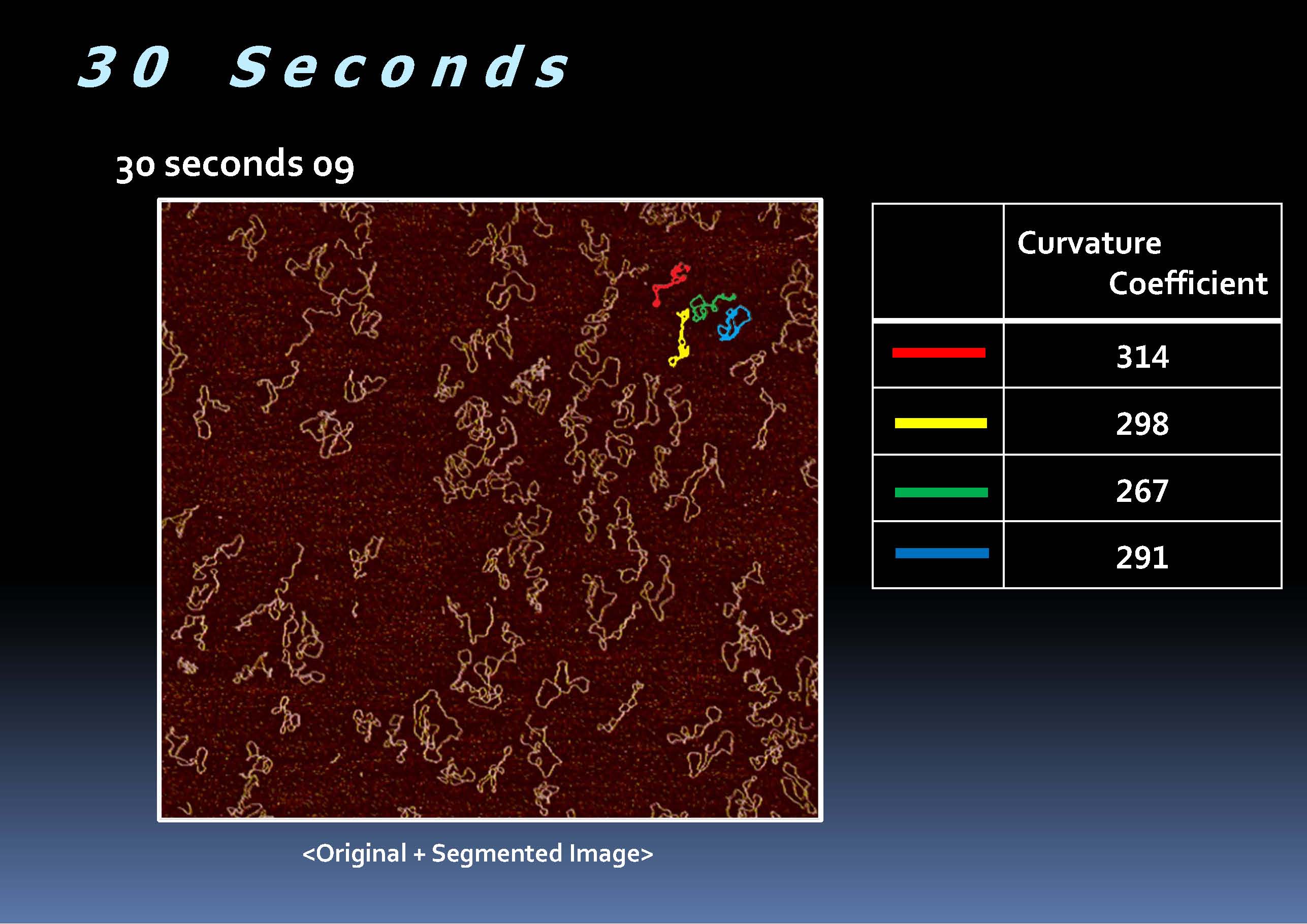

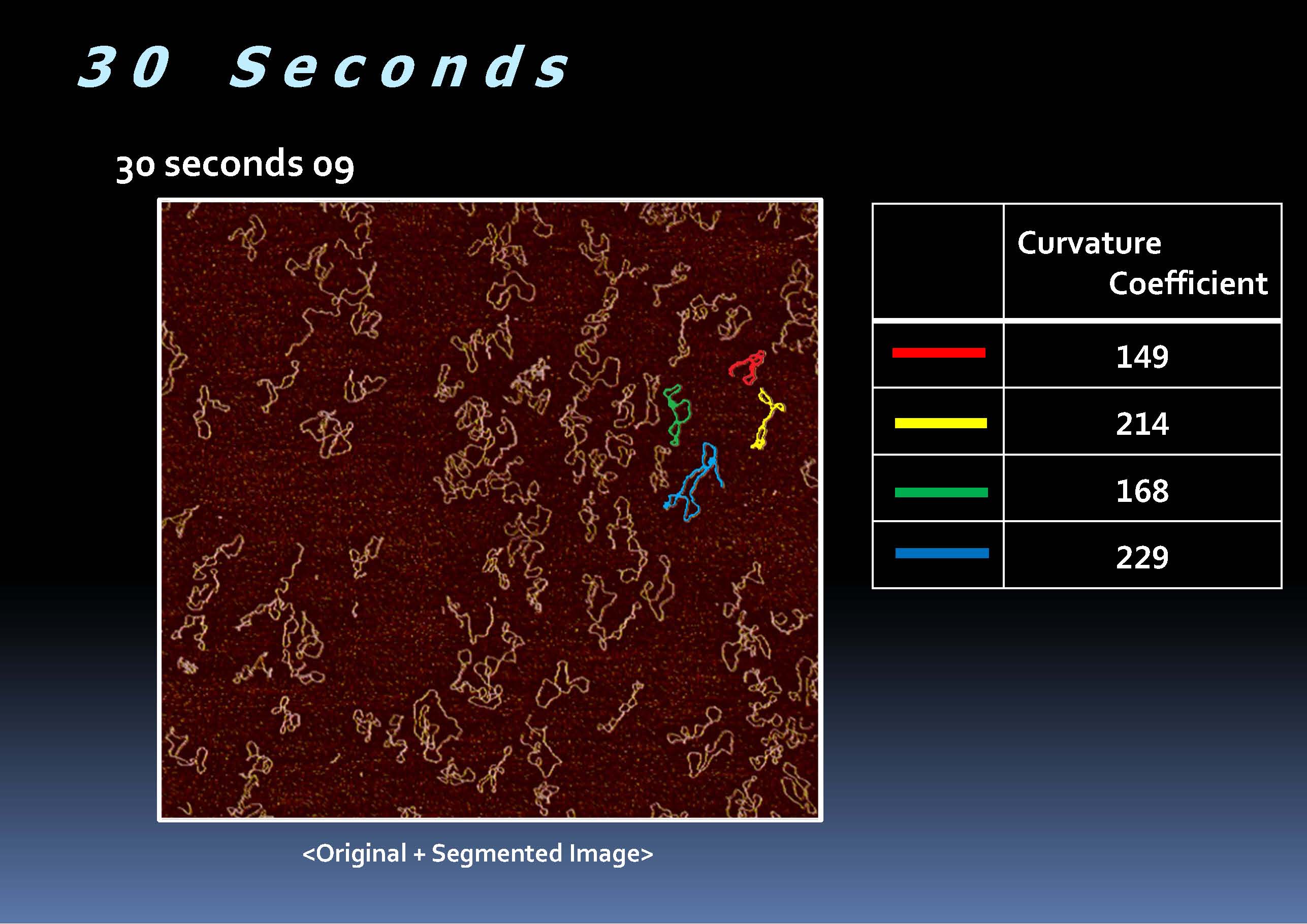

Supplement: Supplementary Information [file srep09846-s2.doc]
